# RBM45 Preferential Binding to m^6^A: Simulations Suggest Synergy of RRM3 and Other Domains

**DOI:** 10.1021/acs.jpcb.6c01856

**Published:** 2026-05-15

**Authors:** Raeyeon Park, Lydia M. Contreras, Phanourios Tamamis

**Affiliations:** † Artie McFerrin Department of Chemical Engineering, 14736 Texas A&M University, College Station, Texas 77843, United States; ‡ McKetta Department of Chemical Engineering, The University of Texas at Austin, Austin, Texas 78712-1589, United States; § Department of Materials Science & Engineering, Texas A&M University, College Station, Texas 77843, United States

## Abstract

RNA-binding motif protein 45 (RBM45) is an RNA-binding protein crucial for brain development and plays a key role in RNA metabolism and disease. Its RNA recognition motif (RRM) domains can recognize GAC-containing motifs, with or without N6-methyladenosine (m^6^A) modification. While its RRM domains individually do not preferentially recognize m^6^A, RBM45 preferentially recognizes m^6^A motifs over unmodified motifs. In this study, we used molecular dynamics (MD) simulations to investigate the binding of a series of RNAs in complex with different RRM domains individually and in complex with RRM3 in the context of the full-length protein. Our study complies with previous experiments and provides in-depth biophysical insights into the binding of unmodified and m^6^A-modified GACG, GACU, and GACA RNA motifs in complex with all RRMs individually. While GACA and GACU bind unfavorably to the individual RRM3 domain, we suggest that they bind favorably to RRM3 in the context of the full-length protein. Importantly, in the context of the entire protein, we present for the first time how RRM3, in combination with additional residues in different RRM domains and linker domains, can synergistically cooperate for m^6^A preferential binding over adenine in the context of GACA and GACU motifs, uncovering the preferential binding of full-length RBM45 for m^6^A observed in previous experiments. The presence of the m^6^A methyl group provides further stability to the RNA, as well as strengthens the particular interactions between RNA and protein, while contributing to additional stabilization of the C-terminal domain with the linker domain between RRM2 and homo-oligomer assembly (HOA). Our simulations highlight the role of various other domains beyond RRMs (i.e., the C-terminal, HOA, and linkers between different RRMs) in the interaction of RBM45 with RNA, particularly m^6^A-modified RNA. Overall, our study elucidated how an RRM class of proteins can preferentially bind m^6^A in the context of its common motif written by methyltransferases. We consider that our study presents the first mechanistic insights into how an RRM domain binds directly and preferentially recognizes m^6^A over adenine in synergy with other domains, which include intrinsically disordered regions.

## Graphical abstract

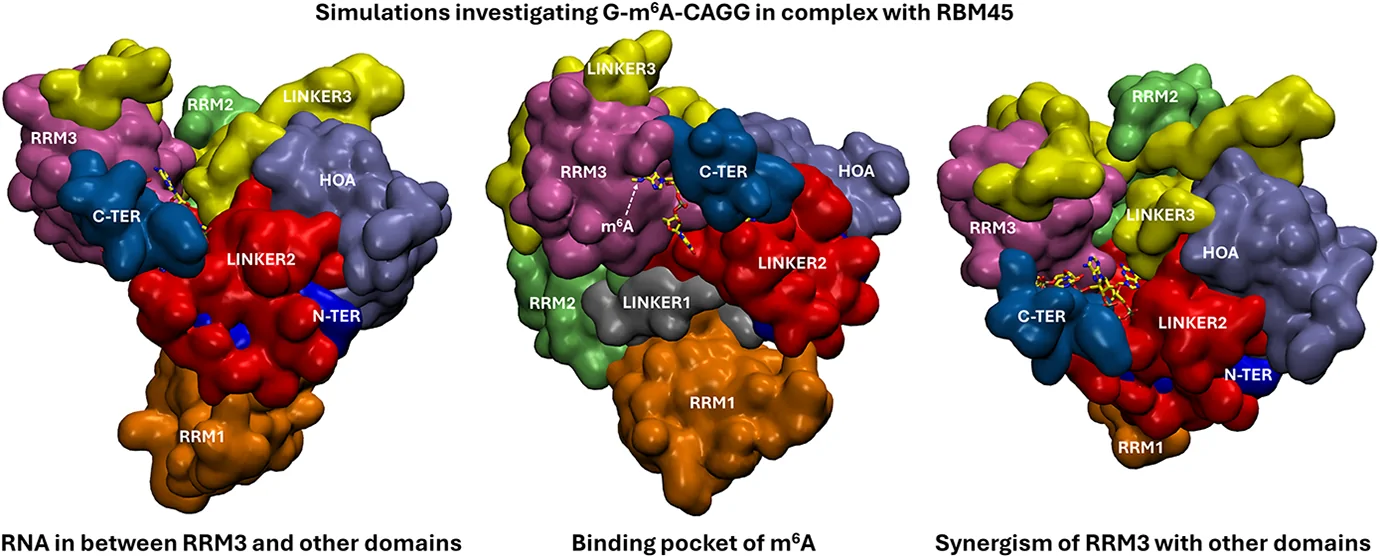

## Introduction

RNA-binding motif protein 45 (RBM45) is an RNA-binding protein (RBP) with important roles in post-transcriptional regulation, including RNA stability,^1,2^ splicing,^3,4^ and localization.^5^ The RNA-binding motif (RBM) protein family, which includes RBM45, represents a subgroup of RBPs that contain RNA recognition motifs (RRMs).^6^ RBM45 comprises three RNA-recognition motif (RRM) domains, two at the N-terminus (RRM1 and RRM2, comprising residues 16–106 and 121–195, respectively) and one at the C-terminus (RRM3, comprising residues 392-463), with a pseudo-RRM domain between RRM2 and RRM3,^7^ referred to as homo-oligomer assembly (HOA, comprising residues 248–324). These domains are connected through linkers, intrinsically disordered domains in the unbound protein structure, while additionally, both termini are also disordered.^8,9^ Generally, RBM45 primarily localizes to the nucleus due to the C-terminal nuclear localization sequence (NLS), but RBM45 can shuttle between the nucleus and cytoplasm.^5,10^

RBM45 is associated with neural development; its expression level is high in the fetal brain and declines as development advances.^11,12^ RBM45 self-associates and regulates mRNA processing, suggesting that the protein may be a component of one or more membraneless nuclear organelles that regulate mRNA splicing and processing, such as nuclear speckles or Cajal bodies.^13^ Abnormalities in RBM45 are associated with neurodegenerative diseases, such as amyotrophic lateral sclerosis (ALS) and frontotemporal lobar dementia (FTLD). In these conditions, RBM45 aggregates in cytoplasmic inclusions, colocalizing with TDP-43,^7,10,14^ another ALS-linked RBP.^7,15^ The RBM45 HOA domain mediates association with proteins TDP-43 and FUS,^10^ as well as HNRNPA1 and HNRNPA2B1.^16^

Choi et al. identified GAC or bipartite GAC sequences as the top-enriched motifs in RBM45 peak regions in both mHippoE-2 and HEK293T cells,^4^ in line with in vitro selection-based studies of RBM45 binding sequences.^17,18^ Additionally, RBM45 has been shown to bind to a couple of enhancer regions within the pre-mRNA of the Parvovirus B19 (including the Intron Splicing Enhancer 3 (ISE3) region), regulating the production of the virus key protein for infection.^19^ Importantly, the RBM45 region of 121 to 318 residue moieties, comprising the RRM2 domain and the flexible linker between RRM2 and the HOA domain, has been shown to be key for binding of RBM45 with ISE3, a fragment containing the GAC motif. RBM45 interacted with the RNA octanucleotide GGGACGGU, which also shares a GAC motif.^19^ Notably, RBM45 regions that include domains with intrinsic disorder could play a key role in facilitating binding to RNA sequences with GAC motifs.

GAC is also the predominant minimal consensus sequence for m^6^A,^20,21^ and m^6^A modification is catalyzed by methyltransferases METTL3 and METTL14 ^22^ and is enriched in ″DRACH″ or ″RRACH″ motifs^7,20,23–26^ (D = A/G/U, R = A/G, H = A/C/U). Choi et al. observed the enrichment of the m^6^A consensus near the RBM45 CLIP peak summits in both mouse and human cells, and highlighted that RBM45 preferentially binds to m^6^A-containing RNA. Choi et al. suggested that the binding of RBM45 to a subset of target RNAs is mediated at least in part by m^6^A and demonstrated that RBM45 can bind to both methylated and unmethylated transcripts, but that the presence of m^6^A enhances its RNA recognition.^4^ While deletion of any single RNA-binding domain (RBD) alone or any two RRMs together does not prevent m^6^A binding, removal of both the HOA and RRM3 domains compromises RNA recognition and preferential binding of RBM45 to m^6^A^4^.

While Choi et al.^4^ showed that RBM45, as a full-length protein, has approximately 3-fold preferential binding of m^6^A over adenine in the context of a large RNA oligo encompassing the GACU motif, two other studies showed that RRM1-RRM2 ^27^ and RRM3 ^7^ of RBM45 do not have preferential binding of m^6^A using sequence motifs comprising GACG and G-m^6^A-CG motifs. Thereby, Chen et al. provided two possible reasons for this: either the HOA domain, not included in their biochemical and structural studies, affects the binding of RRM3 to m^6^A, or N6 methylation of adenine may destabilize the Watson–Crick base pairing, affecting the secondary structure of RNA and predisposing the N6 methylated GACG motifs to adopt a single-stranded conformation.^7^ Thus, while RRM3 is highly selective for GACG sequences, RRM1–RRM2 is not, and they bind GACG sequences comparably to GACA and GACU.^7,27^

Three classes of m^6^A reader proteins have been previously reported.^28^ While the binding of m^6^A to YTH readers is well understood, involving a YTH domain to directly bind the m^6^A base (Class I of Zhou et al.^28^), the binding of m^6^A to many other proteins, particularly RRM-containing proteins, is only partially understood. A few RRM-containing proteins have been investigated for their capacity to bind m^6^A, primarily HNRNPG, HNRNPC, and HNRNPA2B1. An indirect switch m^6^A reading mechanism was depicted for HNRNPC and HNRNPG, in which the m^6^A-mediated destabilization of an RNA hairpin exposes a single-stranded HNRNPC binding motif^29,30^ (Class II of Zhou et al.^28^). In this case, m^6^A modification of RNA destabilizes Watson–Crick base-pairing and increases the accessibility of a single-stranded RNA motif, which is recognized by the m^6^A reader protein. As for HNRNPA2B1, while one study proposed HNRNPA2B1 to be a nuclear reader of the m^6^A mark, suggesting that HNRNPA2B1 can preferentially associate with m^6^A-containing RNA in vitro as partly mediated by the RRM1 motif,^31^ a subsequent study suggested an indirect switch mechanism.^32^ Our studies have recently suggested that HNRNPA2B1 is not a (selective) binder but has a high affinity for m^6^A in a sequence-dependent manner.^33^ This can be attributed to the strong interactions conferred by AGG and UAG motifs rather than adenine or m^6^A in the GGACU motif, which are predicted to interact weakly, intercalating between the two RRMs or positioned outward.^33^ An additional Class III, according to Zhou et al.,^28^ comprises cases in which RBDs and their flanking regions work together to selectively bind m^6^A-containing transcripts. This class was suggested for IGF2BPs, for which the KH3-4 didomain alone was insufficient for selective binding to m^6^A-containing RNAs; the authors suggested that regions flanking the KH domains could also contribute to m^6^A selectivity.^34^ Nevertheless, there is need to provide structure-based mechanistic insights into RBPs in class III, demonstrating the selectivity of m^6^A over adenine.

Interestingly, in comparison to many other RRM-containing proteins, RBM45 represents an exceptional and intriguing case of m^6^A binding by RRM domains. Its RRM1-2 (RRM1 and RRM2), as well as RRM3, have approximately similar high-affinity binding for unmodified and m^6^A-modified RNAs comprising GACG, while RRM1–RRM2 (but not RRM3) have approximately similar high-affinity binding for unmodified and m^6^A-modified RNAs comprising GACA and GACU.^7,27^ Considering that GACA and GACU overlap with the ″DRACH″ or ″RRACH″ motifs, where m^6^A is typically found, RBM45 represents an exemplary case to shed light on m^6^A recognition by RRMs. Overall, the mechanism through which RBM45, in its entirety as a full-length protein, preferentially binds to m^6^A-containing motifs over unmodified GACA and GACU motifs is still unclear. Furthermore, the mechanism by which RBM45 RRM1–RRM2 interacts with GACG, GACA, and GACU, favorably, while RRM3 has sequence selectivity for GACG compared to GACA and GACU,^7,27^ is also unclear. Our study utilized computational methods, predominantly molecular dynamics (MD) simulations and biophysical analysis, to investigate m^6^A binding to RBM45 RRM1–RRM2, and RRM3 individually, as well as to the RRM3 domain in the context of the full-length protein to decouple the role of each RRM in RNA binding. Our results present for the first time the mechanisms of m^6^A binding to RRM1–RRM2 and RRM3 individually. Additionally, no preferential binding was observed when investigating each of the RRMs individually, in agreement with previous studies.^4,7,27^ We additionally show how GACG motifs are more favorable to GACU and GACA motifs for RRM3 binding. Importantly, we present for the first time how, in the context of the full-length RBM45 protein, RRM3 can synergistically cooperate with other domains to directly bind m^6^A preferentially compared to adenine.

## Methods

### RNAs in Complex with RRM1–RRM2 and RRM3 Domains

Molecular modeling of the RRM1–RRM2 domains of RBM45 in complex with RNA was performed based on the crystal structure of RRM1 and RRM2 of RBM45.^27^ Notably, in the X-ray structure (7CSZ^27^), only two 5-nucleotide DNA segments, each containing a GAC motif, were observed (an 11-nucleotide CGACGGGACGC DNA containing two GAC motifs was used for cocrystallization). The two DNAs were resolved to be bound in an antiparallel manner to the positively charged patches of RBM45 RRM1–RRM2.^27^ The distance between each end of the two DNAs did not allow for direct linking^27^; however, interestingly, according to the authors, the 5′ ends of the RRM1- and RRM2-bound DNAs were proximal to the 3′ ends of the RRM1- and RRM2-bound DNAs in the symmetrical molecule, respectively, suggesting that a DNA containing two GAC motifs might mediate crystal packing.^27^ In our study, we used the experimentally resolved structure PDB: 7CSZ^27^ as a starting point to model 5′-GACGGGACGC-3′ and 5′-GGACGGGACG-3′ binding to RRM1 and RRM2, respectively. The former and latter correspond to the experimentally resolved fragments in complex with RRM1 and RRM2, respectively, from the original 11-nucleotide used in the X-ray studies.^27^ Thus, RNA complexes with both RRM1 and RRM2 were considered concurrently in the complex, in accordance with the presence of two strands in the experimentally resolved structure,^27^ and are referred to as RRM1-RRM2. Notably, our studies enabled us to computationally decompose and investigate independently RNA binding to RRM1 and RRM2. The initial conformation was used as a basis to model and simulate RRM1-RRM2 in complex with a series of RNA 10-mers to study the effect of different canonical and noncanonical (m^6^A) substitutions in the context of RRM1-RRM2 binding (Table 1, Entries 1–11); triplicate runs were performed. The sequence of the experimentally resolved fragment (Entry 1) was used as a basis to model the rest of the RNA sequences investigated. The RNA–protein complexes investigated, along with the RNAs of interest for analysis, are described analytically in Table 1. Molecular modeling of the RRM3 domain of RBM45 in complex with RNA was performed based on the crystal structure of the RRM3 of RBM45 in complex with a 6-mer DNA 5′-GACGCA-3′ (PDB: 8WQ5).^7^ This was used as a basis to model RRM3 in complex with a series of RNA 6-mer, to study the effect of different canonical and noncanonical (m^6^A) substitutions in the context of RRM3 binding (Table 1, Entries 12–17). In this case, the sequence of the experimentally resolved fragment was different from the sequence of the motifs for which affinities were measured in previous experiments.^7^ Initial refinement of the complex was performed with RRM3 in complex with the 5′-GACGGA-3′ RNA sequence (comprising G at position 5), with 2 ns equilibration, followed by three production runs of 200 ns each. The minimum binding free energy snapshot was identified and extracted as the refined structure and used as a basis to investigate RRM3 in complex with a series of 6-mer RNA sequences (Table 1, Entries 12–17); this snapshot was also used in our clustering analysis as the reference conformation for comparison purposes. In this case, sextuplicate runs were performed. The final selection of sequences under investigation comprises guanine instead of adenine at the last nucleotide position, motivated by guanine being more populated than adenine in the studies of Choi et al.^4^ Also, four of the sequences selected for simulations shared motifs with sequences for which their binding affinities had been experimentally determined (GGACGG, GG-m^6^A-CGG, GGACAG, GGACUG^7^). In summary, RNA strands containing GACG motifs were investigated in complex with RRM1–RRM2 and RRM3 based on experimental studies providing affinities for GACG-containing sequences,^7,27^ while RNA strands containing GACA and GACU motifs were investigated based on a consensus of canonical and noncanonical (m^6^A) motifs that can be recognized by RBM45 ^4^ (Table 1).

**Table 1:** RNA Sequences Simulated in Complex with Different RRM Domains of RBM45^a^

	**RNA in complex with RRM1**	**RNA in complex with RRM2**
entry 1	5′-GACGGGACGC-3′	5′-GGACGGGACG-3′
entry 2	5′-G-**m** ^**6**^ **A**-CGGGACGC-3′	5′-GGACGGGACG-3′
entry 3	5′-GACAGGACGC-3′	5′-GGACGGGACG-3′
entry 4	5′-G-**m** ^**6**^ **A**-CAGGACGC-3′	5′-GGACGGGACG-3′
entry 5	5′-GACUGGACGC-3′	5′-GGACGGGACG-3′
entry 6	5′-G-**m** ^**6**^ **A**-CUGGACGC-3′	5′-GGACGGGACG-3′
entry 7	5′-GACGGGACGC-3′	5′-GG-**m** ^**6**^ **A**-CGGGACG-3′
entry 8	5′-GACGGGACGC-3′	5′-GGACAGGACG-3′
entry 9	5′-GACGGGACGC-3′	5′-GG-**m** ^**6**^ **A**-CAGGACG-3′
entry 10	5′-GACGGGACGC-3′	5′-GGACUGGACG-3′
entry 11	5′-GACGGGACGC-3′	5′-GG-**m** ^**6**^ **A**-CUGGACG-3′
	**RNA in complex with RRM3**	
entry 12	5′-GACGGG-3′	
entry 13	5′-G-**m** ^**6**^ **A**-CGGG-3′	
entry 14	5′-GACAGG-3′	
entry 15	5′-G-**m** ^**6**^ **A**-CAGG-3′	
entry 16	5′-GACUGG-3′	
entry 17	5′-G-**m** ^**6**^ **A**-CUGG-3′	
	**RNA in complex with RRM3:full-length RBM45**	
entry 18	5′-GACAGG-3′	
entry 19	5′-G-**m** ^**6**^ **A**-CAGG-3′	
entry 20	5′-GACUGG-3′	
entry 21	5′-G-**m** ^**6**^ **A**-CUGG-3′	

a For RRM1–RRM2 complexes (Entries 1–11), two RNA strands were included: one in complex with RRM1 and the other with RRM2. In Entry 1, both RNAs in complex with RRM1 and RRM2 were considered as sequences of interest for investigation and analysis: the former for RNA in complex with RRM1 and the latter for RNA in complex with RRM2. In Entries 2–6, the RNA of interest for investigation and analysis is the one bound to RRM1, while in Entries 7–11, the RNA of interest for investigation and analysis is the one bound to RRM2.

### RNAs in Complex with RRM3 in the Context of the Full-Length RBM45 Protein

Choi et al. suggested that the C-terminal RBDs of RBM45, comprising the HOA and RRM3 domains, can work cooperatively to recognize m^6^A^4^. Our studies investigating binding to RRM domains individually complied with the fact that neither RRM1–RRM2 nor the RRM3 of RBM45 individually preferentially bind m^6^A.^7,27^ Thus, we investigate RNA binding to RRM3 in the context of the full-length protein. The RNA complex with RRM3 in the context of the full-length protein RBM45 was initially modeled as follows. A 6-mer RNA 5′-G-m^6^A-CAGG-3′ bound to RRM3 was modeled using the RRM3 of RBM45 in complex with a 6-mer DNA 5′-GACGCAG-3′ (PDB: 8WQ5).^7^ The protein-domain moiety 392-464 from PDB: 8WQ5 ^7^ was subsequently superimposed on the AlphaFold Database model (AF-Q8IUH3-F1-v4)^8,9^ for RBM45 using VMD;^35^ during superposition, nucleic acid binding to RRM3 was preserved as in PDB: 8WQ5.^7^ This resulted in the first initial model of RNA in complex with RRM3 in the context of the full-length protein, representing a chimeric model comprising the modeled structure from AlphaFold Database^8,9^ and PDB: 8WQ5 ^7^. The initial refinement of the complex was performed using energy minimization and equilibration, followed by three production runs of 200 ns each. The last structure of the run with the lowest average binding free energy was identified as the refined structure and was used as a basis to investigate RBM45 (RRM3) in complex with 5′-G-m^6^A-CAGG-3′ (Table 1, Entry 19) and 5′-GACAGG-3′ (Table 1, Entry 18) in sextuplicate runs. Additionally, the same last structure was used to further refine 5′-G-m^6^A-CUGG-3′, using equilibration, followed by three production runs of 200 ns each. The new last structure of the run with the lowest average binding free energy was identified as the refined structure for 5′-G-m^6^A-CUGG-3′ and used as a basis to investigate RBM45 (RRM3) in complex with 5′-G-m^6^A-CUGG-3′ (Table 1, Entry 21) and 5′-GACUGG-3′ (Table 1, Entry 20) in triplicate runs. For these runs, each system was simulated for 300 ns, providing the modeled and refined systems with ample time, upon modeling and refinement, to study the effect of different canonical and noncanonical (m^6^A) substituted RNAs in complex with the full-length RBM45 protein; in this case, the last 200 ns of simulation was used for structural and energetic analysis, thus, the first 100 ns were considered as an additional equilibration time, in line with RMSD calculations.

### Molecular Dynamics Simulations

All-atom explicit solvent MD simulations were performed in triplicate (or sextuplicate in the case of RBM45 (RRM3) in complex with 5′-G-m^6^A-CAGG-3′ and 5′-GACAGG-3′) to investigate the binding properties of different RNA strands in complex with different RRM domains of RBM45. OpenMM^36^ was used to perform simulations with the CHARMM36m force field,^37^ with parameters for RNA^38–41^ and RNA modifications^42^ provided by the CHARMM-GUI Solution Builder.^43–45^ Simulation input files were generated using CHARMM-GUI Solution Builder,^43–45^ with minor adjustments made to the default settings of the simulation given by CHARMM-GUI to optimize the equilibration and production stage run time. The equilibration stage run time was extended to 2 ns, while the production stage run time was 200 ns (with the exception of systems comprising full-length proteins, see below). Each system was solvated in a water box with a 12 Å edge distance, and 0.15 M KCl ions were added to the system, while extra ions were added accordingly for neutralization. The equilibration stage employed a constant volume, with heavy constraints on the protein and RNA backbone of 400 kJ/(mol*nm^2^) and light constraints of 40 kJ/(mol*nm^2^) on the side chains of protein and RNA. The production stage employed a constant pressure of 1 atm, with no constraints applied to any atoms during the production stage. In all the simulations, the temperature was maintained at 300 K using a Langevin thermostat. The Lennard-Jones (LJ) 6–12 (i.e., van der Waals) interactions were smoothed over the range of 10 to 12 Å using the force-based switching function, and the particle mesh Ewald (PME) method was used for the long-range electrostatic interactions, and residues were protonated at pH 7.4. For each simulation run, the simulation snapshots were extracted from the production stage every 1 ns. Thus, 200 snapshots were analyzed for each simulation run, and a total of 600 snapshots were analyzed for each complex, unless otherwise specified. Simulations of different RNAs in complex with the full-length RBM45 protein were performed for 300 ns, with the last 200 ns extracted for analysis, corresponding to 200 snapshots.

### Energy Calculations and Hydrogen Bond Occupancies

Upon completion of the simulations, the produced snapshots were used for energetic analysis. Vinardo^46^ was used to calculate the binding free energy of protein–RNA complexes, with pdbqt files prepared using OpenBabel.^47^ Vinardo scoring function^46^ was shown to perform better compared to other methods, including MCSS, DVRF20, Vina, ITscorePR, MM-GBMV, and MM-GBSW.^48^ Additionally, VINARDO performed slightly better than MM-GBMV­(SA) when compared to experimentally measured binding free energies in an analogous protein–RNA complex, involving the study of HNRNPA2B1 in complex with m^6^A-modified and unmodified RNAs.^33^

Additionally, we calculated the interaction free energy between residue-nucleotide pairs using in-house CHARMM^49^ scripts employing the GBMV^50^ implicit solvent model method II, as provided by CHARMM-GUI with SA = 0.00542 (kcal * mol^–1^ * Å^–2^),^51–54^ and the SB parameter canceled out in the non-polar calculations of the bound versus unbound states. The setup of the calculations followed previous studies in our lab,^55–60^ and the interaction free energy of the residue-nucleotide pairs was decomposed into polar and non-polar contributions. The process was automated using in-house Fortran and CHARMM programs. For these calculations, 10 snapshots were extracted and analyzed from the production stage of each simulation run at 20 ns intervals; a total of 30 snapshots were analyzed per complex. The Δ*G* interaction free energy was averaged across the extracted snapshots and decomposed into polar and non-polar components to identify the specific interactions contributing to the stabilization of each complex. The polar components included electrostatic and polar solvation interaction free energies, while the non-polar components included van der Waals and non-polar solvation interaction free energies. Additionally, the average interaction free energy per nucleotide was calculated by summing all the residue–nucleotide pairwise interaction free energies per nucleotide, each for polar and non-polar interactions. Hydrogen bond occupancies were calculated using VMD hydrogen bond tools, a distance cutoff of 3.5 Å, and an angle cutoff of 90°, while VMD was additionally used for molecular graphics representations.^35^ Polar interactions between the negatively charged RNA phosphate groups and positively charged RBM45 residues with interaction free energy values lower than −4 kcal/mol were classified as coulombic interactions, whereas interactions with interaction free energy values between −4 and −1.5 kcal/mol were classified as weaker coulombic interactions. The interactions between RNA nucleotides and protein residues discussed in the Results section are based on trajectory-averaged residue-nucleotide interaction free energies and hydrogen bond pairs. The corresponding interaction free energies and occupancies together indicate the strength and frequency of these interactions throughout the simulations.

### RMSD Calculations and Clustering Analysis

RMSD calculations were performed using Wordom.^61,62^ For systems involving RRM1–RRM2 and RRM3 as individual domains, RMSD calculations were performed for the 1–200 ns per run, which corresponded to the entire trajectory, with respect to the average structure per run. For simulations investigating RNAs in complex with RRM3 in the context of the full-length RBM45 protein, RMSD calculations were performed for the 101–300 ns time range, with respect to the average structure per run, for the particular 101–300 ns time duration. Prior to conducting the RMSD calculations, trajectories were structurally aligned to the backbone atoms of the RRM domain of interest and the corresponding binding RNA backbone atoms, with the exception of systems investigating the full-length protein, for which different alignments were considered (Table S1). Backbone atoms were defined as C, CA, N, and O for protein, and P, O1P, O2P, O3′, C3′, C4′, C5′, and O5′ for RNA. For simulations investigating RNAs in complex with RRM3 in the context of the full-length RBM45 protein, additional RMSD calculations were performed for the entire 1–300 ns time range, with respect to the refined structure, which was used as a common starting point for the four systems investigated.

RMSD-based hierarchical clustering was performed using Wordom^61,62^ and a 3.5 Å distance cutoff. Clustering was performed on simulation snapshots of the backbone of RNAs only, taken from simulations of 5′-GACGGA-3′ RNA in complex with RRM3. Clustering of RNA conformations was performed to shed light on the high degree of structural variability of RNA conformers in this complex. The trajectories of the six systems (Table 1, Entries 12–17), each in sextuplicate with 200 ns each, were combined and structurally aligned based on the protein and RNA backbone prior to clustering. Clustering was performed for conformations extracted every 20 ns, thus 120 snapshots for each system, and 720 snapshots in total across six different systems were analyzed. We identified the clusters and the percentage contribution for each of the six systems within each cluster (Table 1, Entries 12–17).

## Results and Discussion

### Simulations of GACG and G-m^6^A-CG in Complex with RRM1–RRM2 and RRM3 Complied with Previously Published Experimental Results

Our study investigated the binding of 5′-GACGGGACGC-3′ and 5′-GGACGGGACG-3′ in complex with RRM1 and RRM2, respectively, with both RRM1 and RRM2 considered concurrently in complex with two RNAs (Entry 1, Table 1), and including all residues resolved in the X-ray studies of Chen et al.^27^ Both RNA strands and RRM domains were predominantly stable within all simulations, as reflected by the RMSD calculations (Table S1A, S1B), except for a single run of the G-m^6^A-CG motif in complex with RRM1 for which additional details are provided below. The binding free energies of the GACG (Entry 1, Table 1) and G-m^6^A-CG (Entry 2 and Entry 7, Table 1) motifs in complex with the RRM1–RRM2 domains of RBM45 were similar, with the unmodified RNA being slightly more favorable than the m^6^A-modified RNA (Figure 1A–B). This is in line with previous experiments showing an overall slightly more favorable (∼0.3 kcal/mol) binding of unmodified RNA to the m^6^A-modified RNA with RRM1–RRM2.^27^ Our study decoupled the binding effects of RRM1 and RRM2 in two ways: (1) The slight favorability of unmodified RNA over m^6^A-modified RNA in the context of GACG motifs could likely be driven mostly by the RRM2 domain, the preference for unmodified vs m^6^A-modified RNA was more favorable for RRM2 (∼0.5 kcal/mol) compared to RRM1 (∼0.1 kcal/mol), at least for GACG motifs. In fact, the experimental binding free energy difference deducted from experimentally measured affinities^27^ is merely an average of the two corresponding computationally predicted values for RRM1 and RRM2, despite the large standard deviations of our computational predictions. (2) The RRM1 domain could possess slightly stronger binding to RRM2, reflected by its lower binding free energy, for both unmodified (GACG) and m^6^A-modified sequences (G-m^6^A-CG) (Figure 1A–B).

**Figure 1:**
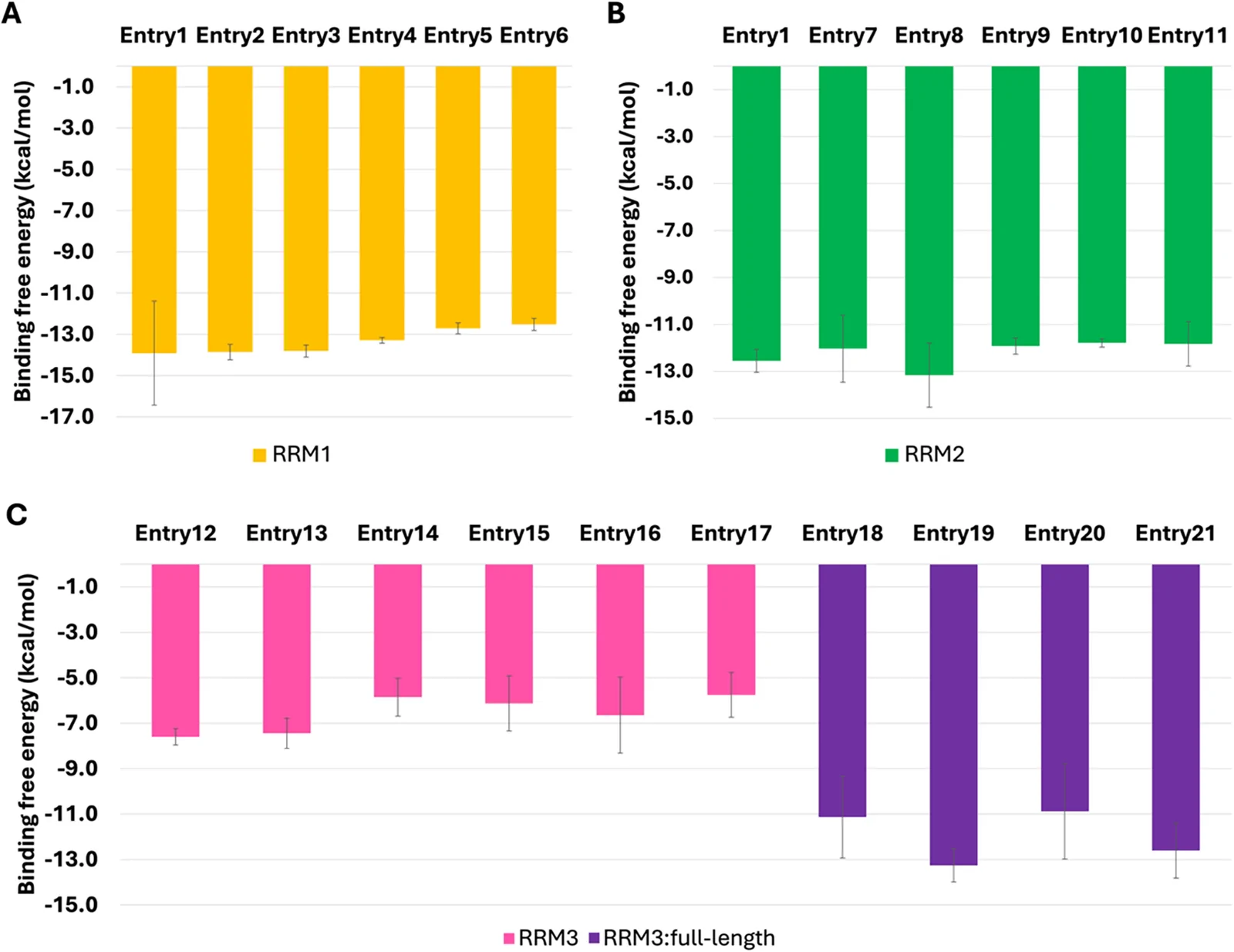
Computationally calculated binding free energies for different RNA sequences in complex with each RRM domain using the extracted simulation snapshots. (A) Binding free energies of unmodified and m^6^A- modified RNA motifs in complex with RRM1 are represented by dark orange bars: GACG (Entry 1), G-m^6^A-CG (Entry 2), GACA (Entry 3), G-m^6^A-CA (Entry 4), GACU (Entry 5), and G-m^6^A-CU (Entry 6). (B) Binding free energies of unmodified and m^6^A-modified RNA motifs in complex with RRM2 are represented by dark green bars: GACG (Entry 1), G-m^6^A-CG (Entry 7), GACA (Entry 8), G-m^6^A-CA (Entry 9), GACU (Entry 10), and G-m^6^A-CU (Entry 11). (C) Binding free energies of unmodified and m^6^A-modified RNA motifs in complex with RRM3 are represented by pink bars: GACG (Entry 12), G-m^6^A-CG (Entry 13), GACA (Entry 14), G-m^6^A-CA (Entry 15), GACU (Entry 16), and G-m^6^A-CU (Entry 17). Binding free energies of unmodified and m^6^A-modified RNA motifs in complex with RRM3 in the context of thefull-length RBM45 protein are represented by purple bars: GACA (Entry 18), G-m^6^A-CA (Entry 19), GACU (Entry 20), and G-m^6^A-CU (Entry 21). The entire nucleotide sequences for each of the entries above are provided in Table 1.

Our study also investigated RNA binding of 5′-GACGGG-3′ (Entry 12, Table 1) with RRM3, including protein residues resolved from the X-ray studies performed by Chen et al.^7^ Similar to the RRM1–RRM2 complex, the RNA strand in complex with the RRM3 domain was predominantly stable within the simulations, as reflected by the RMSD calculations (Tables S1A, S1B). Our results comply with those of Chen et al.,^7^ depicting a similar binding free energy of GACG (Entry 12, Table 1) and G-m^6^A-CG (Entry 13, Table 1) motif-containing sequences in complex with RRM3, and slightly more favorable for the unmodified RNA (Figure 1A–B). For the unmodified sequences in complex with RRM1-RRM2 as well as RRM3, the interactions observed within our simulations also complied with experiments.^7,27^ Notably, within the simulations, the binding properties of adenine to the three RRM domains, individually and in the context of the GAC motif, shared some similarities, including extensive π–π interactions with aromatic residues,^63^ hydrogen bond interactions of its N1 with Q105 E191, and D463 for RRM1, RRM2, and RRM3, respectively (Figures 2, Figures S1–S3).

**Figure 2:**
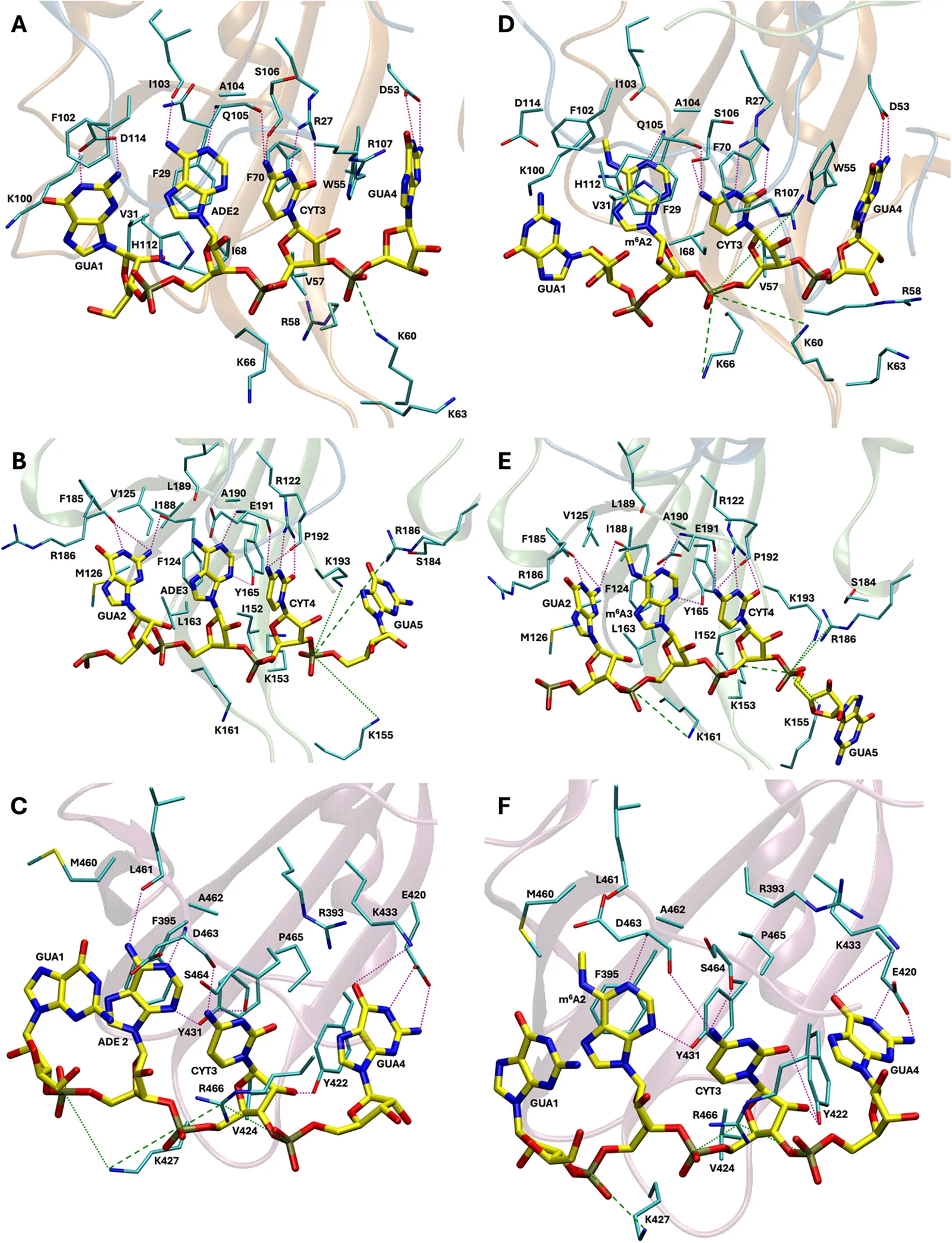
Simulation snapshots of RBM45 of GACG and G-m^6^A-CA motifs bound to the RRM1, RRM2, and RRM3 domains. Interactions between selected nucleotides of the motifs and nearby protein residues in the simulation snapshots are shown. Hydrogen bonds are shown by purple dashed lines, coulombic interactions are shown by green dashed lines; weaker coulombic interactions are shown with less dense green dashed lines. Particular weak coulombic interactions are not indicated by dashed lines for clarity. A full list of hydrogen bonds and coulombic interactions is reported in Tables S2A, S3A, and S4A. (A–C) RBM45 RRM1, RRM2, and RRM3 in complex with 5′-GACGGGACGC-3′ (Entry 1), 5′-GGACGGGACG-3′ (Entry 1), and 5′-GACGGG-3′ (Entry 12), respectively. (D–F) RBM45 RRM1, RRM2, and RRM3 in complex with 5′-G-m^6^A-CGGGACGC-3′ (Entry 2), 5′-GG-m^6^A-CGGGACG-3′ (Entry 7), and 5′-GACGGG-3′ (Entry 13), respectively. Domains are shown in a new cartoon representation, with RRM1 shown in orange, RRM2 shown in green, RRM3 shown in magenta, and the linker between RRM1 and RRM2 shown in pale blue.

Within the simulation of 5′-GACGGGACGC-3′ (Entry 1, Table 1) in complex with RRM1, guanine at position 1 was rather flexible, and in particular cases, it interacted with residues F29, V31, I68, K100, F102, and D114 (Figure 2A, Table S2A, Figure S1). The nucleobase of adenine at position 2 was within the RRM1 binding pocket, comprising residues R27, F29, I68, F70, F102, I103, A104, Q105, R107, and H112 (Figure 2, Table S2A, Figure S1). The nucleobase of cytosine at position 3 was within the RRM1 binding pocket, comprising residues R27, W55, V57, F70, Q105, S106, and R107 (Figure 2, Table S2A, Figure S1), while its backbone phosphate formed coulombic interactions with R27 and R107. The nucleobase of guanine at position 4 was within the RRM1 binding pocket comprising residues R27, D53, W55, and R107, while its backbone phosphate formed coulombic interactions with R27 and R107, and a weaker coulombic interaction with K60 (Figure 2, Table S2A, Figure S1). A complete list of hydrogen bonds and coulombic interactions, both associated with polar interactions, formed in the aforementioned complexes is shown in Table S2A. The favorable residue–nucleotide pairwise interaction free energies are presented in Figure S1.

In the simulation of 5′-GGACGGGACG-3′ (Entry 1, Table 1) in complex with RRM2, the nucleobase of guanine at position 2 was within the RRM2 binding pocket, comprising residues F124, V125, M126, L163, F185, R186, and I188, while its backbone phosphate formed a coulombic interaction with R186 (Figure 2B, Table S3A, Figure S2). The nucleobase of adenine at position 3 was within the RRM2 binding pocket, comprising residues F124, Y165, I188, L189, A190, E191, and K193 (Figure 2B, Table S3A, Figure S2). The nucleobase of cytosine at position 4 was within the RRM2 binding pocket, comprising residues R122, I152, K155, Y165, E191, P192, K193, and N194, while its backbone phosphate formed coulombic interactions with R122 and K193, and a weaker coulombic interaction with K155 (Figure 2B, Table S3A, Figure S2). Due to its positioning between the two homodimers, guanine at position 5 formed interactions with both RRM2 domains. Its nucleobase was within RRM2 of the second homodimer binding pocket, comprising residues I152, K155, and K193, while its backbone phosphate formed coulombic interactions with K155 and K193 (Figure 2B, Table S3A, Figure S2), as well as RRM2 of the first homodimer comprising S184 and R186. The backbone phosphate of this nucleotide formed a coulombic interaction with R186 (Figure 2B, Table S3A, Figure S2). A complete list of hydrogen bonds and coulombic interactions, both associated with polar interactions, formed in the aforementioned complexes is shown in Table S3A, and the favorable residue–nucleotide pairwise interaction free energies are presented in Figure S2.

Within the simulation of 5′-GACGGG-3′ (Entry 12, Table 1) in complex with RRM3, guanine at position 1 was rather flexible, and in particular cases, it interacted mostly with residues F395, M460, L461, D463, and R466. The nucleotides at positions 2, 3, and 4 were overall less flexible. The nucleobase of adenine 2 was primarily within the RRM3 binding pocket, comprising residues F395, K427, Y431, A462, N463, and R466, while its backbone phosphate formed coulombic interactions with K427 and R466 (Figure 2C, Table S4A, Figure S3). The nucleobase of cytosine 3 was primarily within the RRM3 binding pocket, comprising residues R393, Y422, V424, K427, Y431, D463, S464, P465, and R466, while its backbone phosphate formed weaker coulombic interactions with R393, K427, and R466 (Figure 2C, Table S4A, Figure S3). The nucleobase of Guanine 4 was primarily within the RRM3 binding pocket, comprising residues R393, E420, Y422, K433, and R466, while its backbone phosphate formed a coulombic interaction with R466, as well as weaker coulombic interactions with R393 and K433 (Figure 2C, Table S4A, Figure S3). A complete list of hydrogen bonds and coulombic interactions, both associated with polar interactions, formed in the aforementioned complexes is shown in Table S4A, and the favorable residue–nucleotide pairwise interaction free energies are presented in Figure S3.

To our knowledge, our study presents the first analysis of the interactions formed between m^6^A-containing RNA and RBM45. The introduction of m^6^A in place of adenine in the context of the GACG motif resulted in m^6^A maintaining its orientation and binding pocket in relevance to adenine in all cases: 5′-G-m^6^A-CGGGACGC-3′ in complex with RRM1 (Entry 2, Table 1), 5′-GG-m^6^A-CGGGACG-3′ in complex with RRM2 (Entry 7, Table 1), and 5′-G-m^6^A-CGGG-3′ in complex with RRM3 (Entry 13, Table 1). The methyl group of m^6^A was positioned in proximity to F102 (the non-polar part of) Q105 in RRM1 and F124 (the non-polar part of) E191 in RRM2, as well as M460, and (the non-polar part of) D463 in RRM3 (Figure 2D,2E,2F). The introduction of a methyl group in m^6^A results in minor differences compared to adenine.

In the RRM1 complex, m^6^A modification resulted in weakening of polar interactions between m^6^A at position 2 and H112, G4:R107, and G5:R107 (Figure 2A,D, Table 2, Figure S4B). On the contrary, particular polar interactions, including m^6^A with R107, C3:R27, C3:K60, C3:R107, G4:D53, G4:R58, G4:K60, and G4:K63 (Figure 2A,D, Table 2, Figure S4B), were overall improved. In one of three simulation runs, the 5′ and 3′ termini came into closer proximity, resulting in a slight change in the binding pocket of guanine at positions 4 and 5. As a result, in this particular run, polar interactions between G4:D53, G4:R58, and G4:K63 were stronger compared to those between 5′-GACGGGACGC-3′.

**Table 2:** Summary of Intermolecular Polar and Non-Polar Interaction Free Energies between the Selected Nucleotides of G-A/m^6^A-CG and RBM45 Residues of RRM1, RRM2, and RRM3^a^

**nucleotide:RBM45 residues (polar and non-polar interaction free energies)**	
**5′-GACGGGACGC-3′ (entry 1)**	**5′-G-m** ^**6**^ **A-CGGGACGC-3′ (entry 2)**
Ade2:R107 (−1.0, −0.3)	m^6^A 2:R107 (−2.2, −0.7)
Ade2:H112 (−1.3, −1.8)	m^6^A 2:H112 (0.1, −1.6)
Cyt3:R27 (−14.4, −0.9)	Cyt3:R27 (−24.5, 0.1)
Cyt3:K60 (−1.2, −0.2)	Cyt3:K60 (−2.5, −0.6)
Cyt3:R107 (−6.3, −5.7)	Cyt3:R107 (−9.7, −5.6)
Gua4:D53 (−12.9, 0.1)	Gua4:D53 (−17.0, 0.1)
Gua4:K60 (−3.6, −0.5)	Gua4:K60 (−6.6, −0.9)
Gua4:K63 (−0.2, 0.0)	Gua4:K63 (−2.6, −0.3)
Gua4:R107 (−13.3, −2.9)	Gua4:R107 (−9.6, −2.0)
Gua5:R107 (−9.9, −0.3)	Gua5:R107 (−3.5, −0.3)
**5′-GGACGGGACG-3′ (entry 1)**	**5′-GG-m** ^**6**^ **A-CGGGACG-3′ (entry 7)**
Gua2:F124 (−4.2, −5.2)	Gua2:F124 (−3.3, −4.0)
Gua2:I188 (−0.7, −3.2)	Gua2:I188 (−0.4, −2.2)
Ade3:K161 (−0.9, −0.3)	m^6^A 3:K161 (−1.9, −0.6)
Ade3:L189 (−1.8, −1.2)	m^6^A 3:L189 (−0.6, −0.9)
Ade3:E191 (−2.6, −3.6)	m^6^A 3:E191 (−2.5, −2.5)
Cyt4:K193 (−7.9, −3.9)	Cyt4:K193 (−5.3, −3.9)
Gua5:K153 (0, −0.9)	Gua5:K153 (−1.8, −1.2)
Gua5:S184 (−2.1, −1.2)	Gua5:S184 (−0.7, −0.8)
**5′-GACGGG-3′ (entry 12)**	**5′-G-m** ^**6**^ **A-CGGG-3′ (entry 13)**
Ade2:K427 (−4.2, −0.6)	m^6^A 2:K427 (−3.8, −0.2)
Ade2:Y431 (−5.1, −1.7)	m^6^A 2:Y431 (−0.2, −5.4)
Ade2:R466 (−6.0, −0.7)	m^6^A 2:R466 (−7.1, −6.0)
Cyt3:R393 (−4.9, −0.3)	Cyt3:R393 (−1.3, −0.2)
Cyt3:Y422 (−5.5, −1.2)	Cyt3:Y422 (−0.9, −7.0)
Cyt3:K427 (−1.6, −0.8)	Cyt3:K427 (−3.8, −0.2)
Cyt3:R466 (−3.9, −3.9)	Cyt3:R466 (−16.0, −0.6)
Gua4:R393 (−2.7, −0.4)	Gua4:R393 (−2.1, −0.1)
Gua4:E420 (−16.6, −0.1)	Gua4:E420 (−1.7, −0.5)
Gua4:K433 (−2.6, −1.3)	Gua4:K433 (−3.6, −0.5)

a The intermolecular polar and non-polar interaction free energies correspond to the average values (kcal/mol).

In the RRM2 complex, m^6^A modification resulted in weakening the polar interactions between m^6^A at position 3 and L189, and C4:K193, G5:S184 (first homodimer), as well as the non-polar interactions between G2:F124, I188, and m^6^A with E191 (Figure 2B,E, Table 2, Figure S5B). On the contrary, particular polar interactions, including m^6^A with K161 and G5:K153, were improved, but these do not appear to outweigh the improved interactions formed by 5′-GGACGGGACG-3′ (Figure 2B,E, Table 2, Figure S5B).

In the RRM3 complex, m^6^A substitution resulted in weakening of polar interactions between m^6^A at position 2 with K427, Y431, C3:R393, G4:R393 and G4:E420 (Figure 2C,F, Table 4, Figure S6B). On the contrary, particular polar interactions, including m^6^A at position 2 with R466, C3:K427, C3:R466, G4:K433, and R466, and the non-polar interaction between C3:R466, were improved, which do not appear to outbalance the improved interactions formed by 5′-GACGGG-3′ (Figure 2C,F, Table S4A, Figure S6B). Importantly, in both unmodified (GACG) and m^6^A-modified (G-m^6^A-CG) RNA sequences in complex with RRM3, guanine at position 4 was well stabilized by hydrogen bonds with E420 and K433 (Figure 2C,F, Table S4A, Figure S6B).

The aforementioned analysis is based on the average values of the triplicate simulations. A complete list of hydrogen bonds and coulombic interactions, both associated with polar interactions, formed in the aforementioned complexes, is shown in Tables S2A, S3A, and S4A. The strength of favorable (Δ*G* < 0) residue–nucleotide pairwise interaction free energies, decomposed into polar and non-polar components, are shown in Figures S1–S3, S4A, S5A, and S6A. Also, a comparison between the unmodified and m^6^A-modified sequences is shown in Figures S4B, S5B, and S6B.

### Analysis of GACA, G-m^6^A-CA, GACU, and G-m^6^A-CU in Complex with RRM1–RRM2 Showed No Preferential Binding of m^6^A Relative to Adenine

The aforementioned analysis provided insights into the role of m^6^A in addition to adenine within the context of GACG motifs, which were the focus of previous experimental resolution studies,^7,27^ in complex with RRM1–RRM2 and RRM3, individually. Subsequently, our investigation focused primarily on RNA strands comprising GACA and GACU motifs, which are biologically relevant both in terms of comprising motifs of m^6^A that are written by methyltransferases^22^ and corresponding to the top-enriched motifs in RBM45 peak regions in both mHippoE-2 and HEK293T cells.^4^ Therefore, we investigated RNAs comprising GACA, G-m^6^A-CA, GACU, and G-m^6^A-CU sequence motifs in complex with RRM1–RRM2 (entries 3–11). In all simulations, RNA strands and RRM domains were predominantly stable, as reflected by the RMSD calculations (Table S1A). In the context of both GACA and GACU, m^6^A-modified sequences interacted with the RRM1–RRM2 domains on par or slightly less favorable compared to unmodified sequences, irrespective of an adenine or uridine at position 4 for RRM1–RRM2 (Figure 1A–B). Similar to what was mentioned above for GACG-containing motifs, the slight favorability of unmodified RNA over m^6^A-modified RNA in the context of GACA motifs could likely be driven by the RRM2 domain. Additionally, the presence of adenine at position 4 resulted in more favorable binding than uridine for RRM1–RRM2, for both unmodified and m^6^A-modified sequences. Notably, both the absence of experimentally resolved structures for GACA, G-m^6^A-CA, GACU, and G-m^6^A-CU sequence motifs in complex with RRM1–RRM2 of RBM45, as well as the biological relevance of these motifs,^4^ highlights the importance of providing a detailed description of interactions and differences, which is provided below.

#### Binding to G-m^6^A-CA Was Not Preferential Relative to GACA for RRM1

Simulations of 5′-GACAGGACGC-3′ in complex with RRM1 (Entry 3, Table 1) showed that guanine at position 1 was rather flexible, and in particular cases, it interacted mostly with residues D463 and R466 (Figure 3A, Table S2B, Figure S7). The nucleobase of adenine at position 2 was within the RRM1 binding pocket comprising residues F29, I68, F70, F102, I103, A104, and Q105 (Figure 3A, Table S2B, Figure S7). The nucleobase of cytosine at position 3 was within the RRM1 binding pocket comprising residues R27, W55, V57, F70, Q105, S106, and H112, while its backbone phosphate formed coulombic interactions with R27, R107, and a weaker coulombic interaction with K60 (Figure 3A, Table S2B, Figure S7). The nucleobase of adenine at position 4 was within the RRM1 binding pocket comprising residues W55 and R107, while its backbone phosphate formed a coulombic interaction with R107 and a weaker coulombic interaction with K60 (Figure 3A, Table S2B, Figure S7).

**Figure 3:**
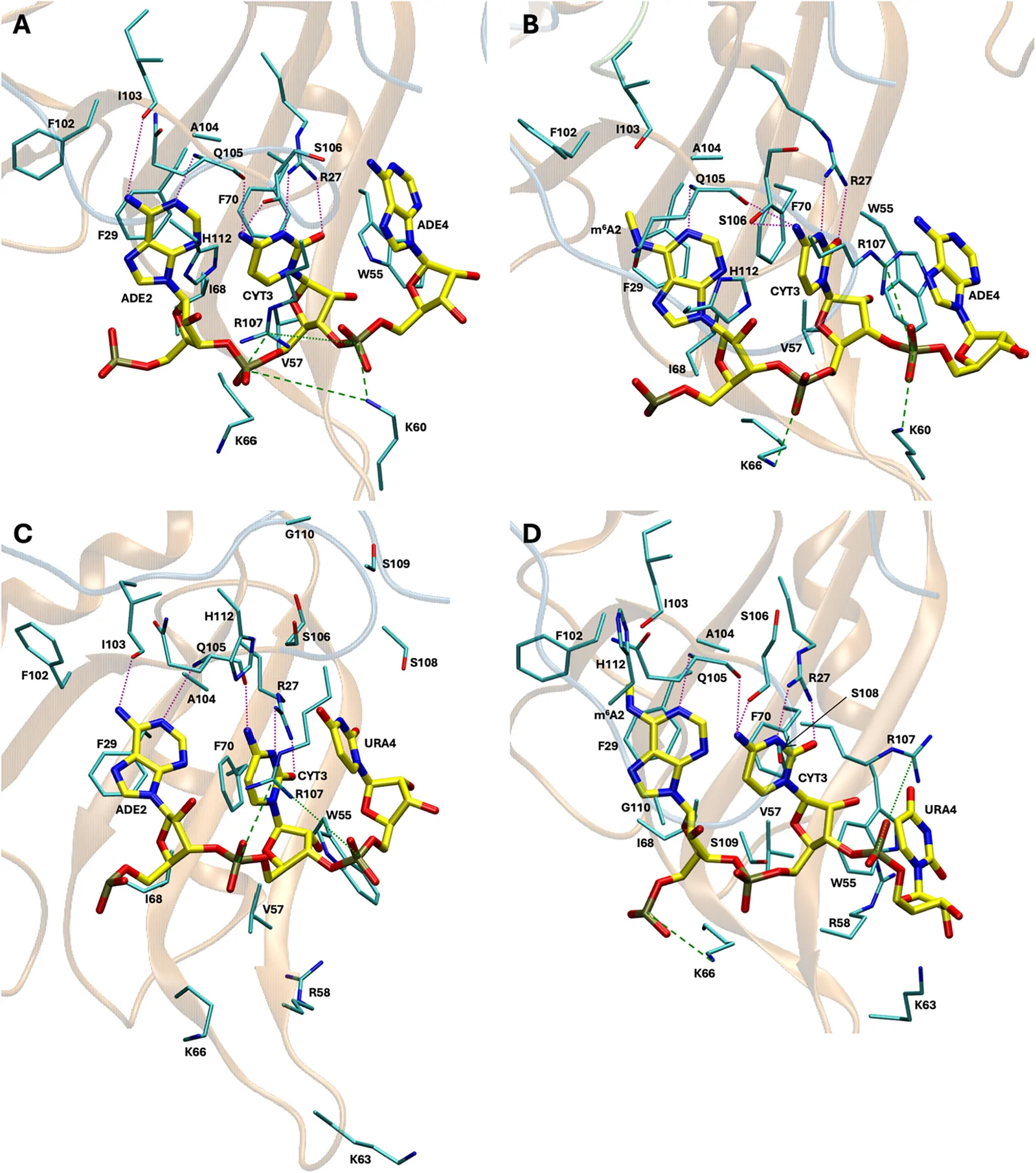
Simulation snapshots of RBM45 of the GACA, G-m^6^A-CA, GACU, and G-m^6^A-CU motifs bound to the RRM1 domain. Interactions between the selected nucleotides of the motifs and nearby protein residues in the simulation snapshots are shown. Hydrogen bonds are shown by purple dashed lines, coulombic interactions are shown by green dashed lines; weaker coulombic interactions are shown by less dense green dashed lines. Particular weak coulombic interactions are not indicated by dashed lines for clarity. A full list of hydrogen bonds and coulombic interactions is reported in Table S2B. (A–B) RBM45 RRM1 in complex with 5′-GACAGGACGC-3′ (Entry 3) and 5′-G-m^6^A-CAGGACGC-3′ (Entry 4), respectively. (C–D) RBM45 RRM1 in complex with 5′-GACUGGACGC-3′ (Entry 5) and 5′-G-m^6^A-CUGGACGC-3′ (Entry 6), respectively. Domains are shown in a new cartoon representation, with RRM1 shown in orange, RRM2 shown in green, and the linker between RRM1 and RRM2 shown in pale blue.

The introduction of m^6^A at the second position, 5′-G-m^6^A-CAGGACGC-3′ (Entry 4, Table 1) resulted in few differences between the two systems, providing insights into why adenine was slightly more favorable than m^6^A. The methyl group of m^6^A was in closer proximity to F102 (the non-polar part of) Q105 in RRM1 (Figure 3B). While in 5′-G-m^6^A-CAGGACGC-3′, there were some improved polar interactions, primarily between m^6^A at position 2 with K66, H112, and C3:R27, and C3:K66, these do not appear to outweigh the improved interactions formed by 5′-GACAGGACGC-3′ (Figure 3A,B, Table 3, Table S2B, and Figure S8). A comparison of the strength of the residue–nucleotide interaction free energies between the two complexes is shown in (Figure S8B), where the binding pockets and corresponding interactions between the two systems were highly similar to each other, except for a few minor differences noted above (Figure 3A,B). This analysis uncovered interactions associated with the slight favorability of adenine compared to m^6^A when comparing GACA (Entry 3, Table 1) and G-m^6^A-CA (Entry 4, Table 1) motifs, as depicted in our binding free energy calculations (Figure 1A).

**Table 3:** Summary of Intermolecular Polar and Non-Polar Interaction Free Energies between the Selected Nucleotides of the G-A/m^6^A-C-A/U and RBM45 Residues of RRM1 and RRM2 Domains^a^

**nucleotide:RBM45 residues (polar and non-polar interaction free energies)**	
**5′-GACAGGACGC-3′ (entry 3)**	**5′-G-m** ^**6**^ **A-CAGGACGC-3′ (entry 4)**
Ade2:K66 (−1.1, −0.2)	m^6^A 2:K66 (−2.1, −0.7)
Ade2:H112 (−0.2, −1.6)	m^6^A 2:H112 (−2.3, −2.2)
Cyt3:R27 (−11.7, −0.6)	Cyt3:R27 (−20.7, 0.2)
Cyt3:K66 (−0.8, −0.3)	Cyt3:K66 (−1.9, −0.3)
**5′-GACUGGACGC-3′ (entry 5)**	**5′-G-m** ^**6**^ **A-CUGGACGC-3′ (entry 6)**
Ade2:K66 (−1.2, −0.3)	m^6^A 2:K66 (−3.2, −0.3)
Ura4:R107 (−9.4, −4.9)	Ura4:R107 (−1.4, −1.1)
**5′-GGACAGGACG-3′ (entry 8)**	**5′-GG-m** ^**6**^ **A-CAGGACG-3′ (entry 9)**
Gua2:F124 (−4.2, −4.9)	Gua2:F124 (−2.5, −3.5)
Gua2:M126 (−0.5, −4.8)	Gua2:M126 (−0.3, −3.2)
Gua2:R186 (−10.2, −3.3)	Gua2:R186 (−6.6, −3.2)
Gua2:I188 (−0.7, −3.1)	Gua2:I188 (−0.3, −1.7)
Cyt4:R122 (−14.8, −0.5)	Cyt4:R122 (−11.5, −0.3)
Ade5:K153 (−1.5, −2.9)	Ade5:K153 (−1.3, −1.2)
**5′-GGACUGGACG-3′ (entry 10)**	**5′-GG-m** ^**6**^ **A-CUGGACG-3′ (entry 11)**
Gua2:R186 (−10.1, −3.1)	Gua2:R186 (−10.1, −3.4)
Ade3:K161 (−1.0, −0.4)	m^6^A 3:K161 (−2.9, −0.9)
Cyt4:R122 (−21.4, 0.3)	Cyt4:R122 (−21.4, −0.6)
Cyt4:K155 (−1.4, −0.5)	Cyt4:K155 (−2.6, −0.9)
Ura5:K155 (−4.4, −0.7)	Ura5:K155 (−12.1, −2.4)

a The intermolecular polar and non-polar interaction free energies correspond to the average values (kcal/mol).

#### Binding to GACU was Slightly Less Favorable Compared to GACA, and Binding to G-m^6^A-CU Was not Preferential Relative to GACU for RRM1

Our investigation subsequently focused on the role of adenine and uridine at position 4 by comparing 5′-GACUGGACGC-3′ (Entry 5, Table 1) to 5′-GACAGGACGC-3′ (Entry 3, Table 1). The binding free energy of 5′-GACUGGACGC-3′ was slightly less favorable compared to that of 5′-GACAGGACGC-3′ in complex with RRM1 (Figures 1A, 3A,C, S7, and S9). Additionally, the per-nucleotide interaction free energy of adenine at position 4 was more favorable compared to that of uridine (Figure S10A, S10B). This can be attributed to weakened polar interactions between U4:K60 and R107 (Figure 3A,C, Tables S2, Figures S7, and S9), as well as weakened non-polar interactions between U4:W55 (Figure 3A,C, Table S2B, Figures S7 and S9). This appears to give rise to the slightly unfavorable binding free energy of GACU (Entry 5, Table 1) and GACA (Entry 3, Table 1) motifs to RRM1 (Figure 1A).

The introduction of m^6^A at the second position in 5′-G-m^6^A-CUGGACGC-3′ compared to 5′-GACUGGACGC-3′ (Figures 3C,D, S9, and S10) resulted in weakened polar and non-polar interactions formed by m^6^A at position 2 and I103, C3:Q105, and U4:R107 (Figure 3C,D, Table 3, Table S2B, and Figure S11). Nonetheless, in 5′-G-m^6^A-CUGGACGC-3′, other polar interactions were improved primarily between m^6^A at position 2 and K66, C3:S109, C3:G110, and U4:K63. These do not appear to outbalance the improved interactions formed by 5′-GACUGGACGC-3′ (Figure 3C,D, Table 3, Table S2B, and Figure S11). This analysis uncovered interactions associated with adenine that were approximately on par with m^6^A when comparing GACU (Entry 5, Table 1) and G-m^6^A-CU (Entry 6, Table 1) motifs, as depicted in our binding free energy calculations (Figure 1A). Analogously to the unmodified sequences (Figure S10A and S10B), for modified sequences, the per-nucleotide interaction free energy of adenine at position 4 was more favorable than that of uridine (Figure S10C and S10D).

The aforementioned analysis is based on the average values of the triplicate simulations. A complete list of hydrogen bonds and coulombic interactions, both associated with polar interactions, formed in the aforementioned complexes is shown in Table S2B. The strength of favorable (Δ*G* < 0) residue–nucleotide pairwise interaction free energies, decomposed into polar and non-polar components, are shown in Figures S7, S8A, S9, and S11A, while per-nucleotide interaction free energies are shown in Figure S10. Also, a comparison between the unmodified and m^6^A-modified sequences is shown in Figures S8B and S11B.

#### Binding to G-m^6^A-CA Was Not Preferential Relative to GACA for RRM2

Our investigation subsequently focused on studying the binding pocket within RRM2 in complex with 5′-GGACAGGACG-3′ (Entry 8, Table 1). The nucleobase of adenine at position 3 was within the RRM2 binding pocket, comprising residues F124, Y165, I188, L189, A190, and E191 (Figure 4A, Table S3B, Figure S12). The nucleobase of cytosine at position 4 was within the RRM2 binding pocket, comprising residues R122, L152, Y165, E191, P192, and K193, while its backbone phosphate formed a coulombic interaction with K193 (Figure 4A, Table S3B, Figure S12). The nucleobase of adenine at position 5 was within the RRM2 binding pocket of the second homodimer, comprising residues I152, K153, N154, and K161, while its backbone phosphate formed coulombic interactions with K155 and K193 (Figure 4A, Table S3B, Figure S12). Additionally, adenine at position 5 formed coulombic interactions through its backbone with R186 of the RRM2 of the first homodimer (Figure 4A, Table S3B, Figure S12). The introduction of m^6^A modification at the third position of the 5′-GGACAGGACG-3′ resulted in a few differences between the two systems’ binding pockets, including weakening of polar interactions between m^6^A at position 3 with L189, C4:R122, C4: K193, and A5:K193, as well as weakening of non-polar interactions between the apolar moieties of A5:K153 (Figure 4A,B, Table 3, Table S3B and Figure S13). Overall, the orientation and binding pocket of m^6^A were reminiscent of adenine, with the methyl group interacting with F124 (the non-polar part of) E191 in RRM2 (Figure 4A,B, Table 3, Table S3B, and Figure S13). This analysis uncovered interactions associated with the slight favorability of adenine compared to m^6^A when comparing the GACA (Entry 8, Table 1) and G-m^6^A-CA (Entry 9, Table 1) motifs, as depicted in our binding free energy calculations (Figure 1B).

**Figure 4:**
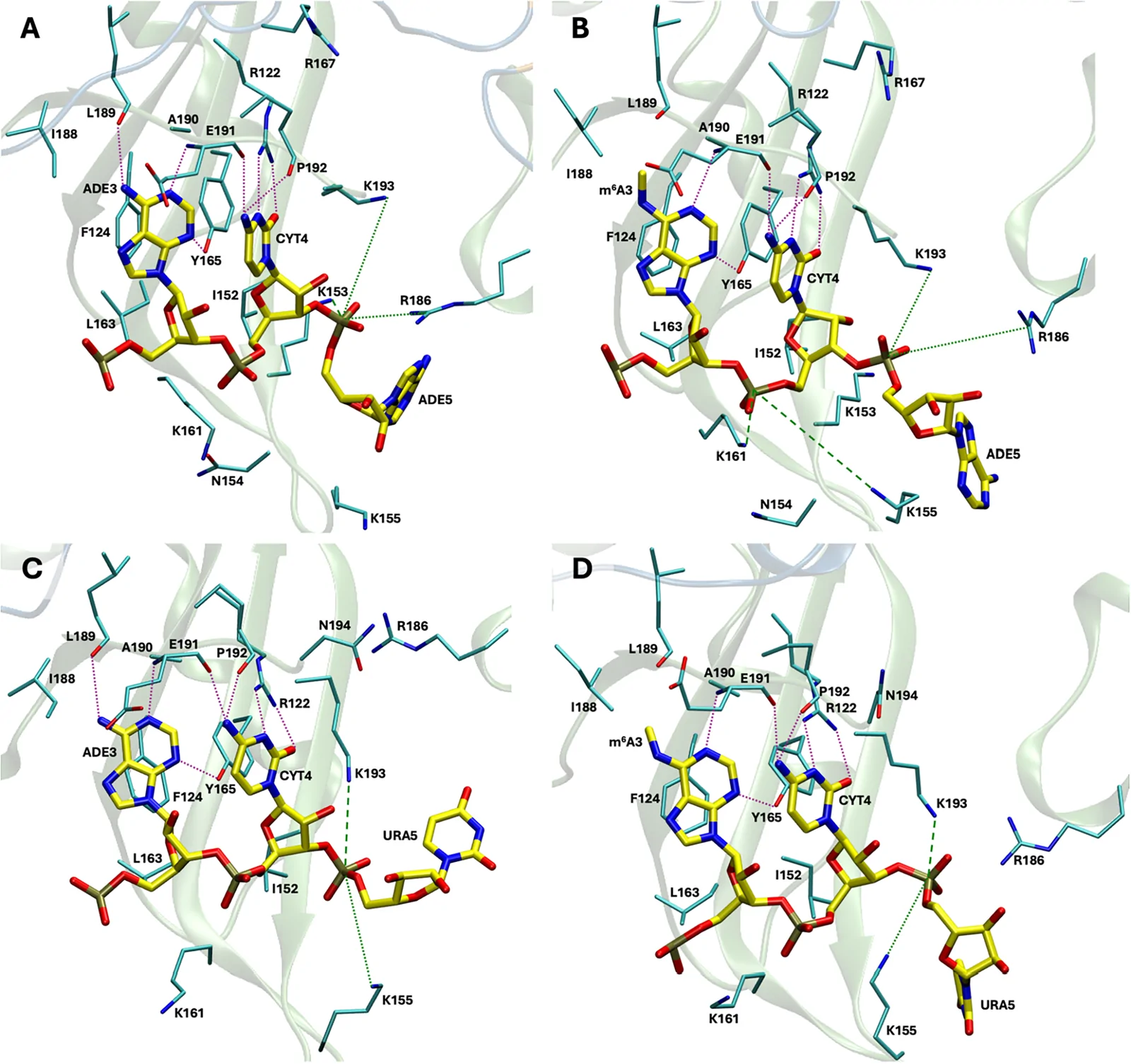
Simulation snapshots of RBM45 of GACA, G-m^6^A-CA, GACU, and G-m^6^A-CU motifs bound to the RRM2 domain. Interactions between the selected nucleotides of the motifs and nearby protein residues in the simulation snapshots are shown. Hydrogen bonds are shown by purple dashed lines, coulombic interactions are shown by green dashed lines, and weaker coulombic interactions are shown by less dense green dashed lines. Particular weak coulombic interactions are not indicated by dashed lines for clarity. A full list of hydrogen bonds and coulombic interactions is reported in Table S3B. (A–B) RBM45 RRM2 in complex with 5′-GGACAGGACG-3′ (Entry 8) and 5′-GG-m^6^A-CAGGACG-3′ (Entry 9), respectively. (C–D) RBM45 RRM2 in complex with 5′-GGACUGGACG-3′ (Entry 10) and 5′-GG-m^6^A-CUGGACG-3′ (Entry 11), respectively. Domains are shown in a new cartoon representation, with RRM2 shown in green, RRM1 shown in orange, and the linker between RRM1 and RRM2 shown in pale blue.

#### Binding to GACU Was Slightly Less Favorable Compared to GACA, and G-m^6^A-CU Binding Was Not Preferential Relative to GACU for RRM2

The binding of 5′-GGACUGGACG-3′ (Entry 10, Table 1) was investigated to compare the roles of adenine and uridine at position 5. The binding of 5′-GGACUGGACG-3′ was similar to that of 5′-GGACAGGACG-3′ (Figures 4A,C, S12, and S14). The binding free energy of 5′-GGACUGGACG-3′ was less favorable compared to that of 5′-GGACAGGACG-3′ (Figure 1B), while additionally, the interaction free energy per nucleotide of uridine at position 5 was less favorable compared to that of adenine (Figure S15A and S15B). This can be attributed to differences, including weaker non-polar interactions between U5:K153 and U5:K155, and polar interactions between U5:K153, U5:K161, and U5:K193 (Figures 4A,C, S12, and Figure S14) of the first homodimer. The introduction of m^6^A modification at the third position of the 5′-GGACUGGACG-3′ sequence resulted in slightly different interactions within the binding pocket between the two systems. This slight difference can be attributed to the weakened polar interactions formed between A3:L189 (Figure 4C,D, Table 3, Table S3B, and Figure S16). While m^6^A also appeared to form improved polar interactions primarily between m^6^A at position 3 with K161, C4:K155, C4:K161, and U5:K155 (Figure 4C,D, Table 3, Table S3B, and Figure S16), these do not appear to outweigh the improved interactions formed by adenine. A comparison of the two complexes is shown with ΔΔ*G* values (Figure S16), where the binding pockets of the two systems are highly similar to each other (Figure 4C,D). This analysis uncovered interactions associated with the slight favorability of adenine compared to m^6^A when comparing GACU (Entry 10, Table 1) and G-m^6^A-CU (Entry 11, Table 1), Table 1 motifs, as depicted in our binding free energy calculations (Figure 1B). Analogously to the unmodified sequences (Figure S15A and S15B), for modified sequences, the per-nucleotide interaction free energy of adenine at position 4 was more favorable compared to that of uridine (Figure S15C and S15D).

The aforementioned analysis is based on the average values of the triplicate simulations. A complete list of hydrogen bonds and coulombic interactions, both associated with polar interactions, formed in the aforementioned complexes, is shown in Table S3B. The strength of favorable (Δ*G* < 0) residue–nucleotide pairwise interaction free energies, decomposed into polar and non-polar components, are shown in Figures S12, S13A, S14, and S16A, while per-nucleotide interaction free energies are shown in Figure S10. Also, a comparison between unmodified and m^6^A-modified sequences is shown in Figures S13B and S16B.

### GACA, G-m^6^A-CA, GACU, and G-m^6^A-CU in Complex with RRM3 Could Not Tolerate A/U at Position 4 Because of Significantly Unfavorable Interactions

We investigated the binding properties of six RNA sequences in complex with RRM3:5′-GACGGG-3′ (Entry 12, Table 1), 5′-G-m^6^A-CGGG-3′ (Entry 13, Table 1), 5′-GACAGG-3′ (Entry 14, Table 1), 5′-G-m^6^A-CAGG-3′ (Entry 15, Table 1), 5′-GACUGG-3′ (Entry 16, Table 1), and 5′-G-m^6^A-CUGG-3′ (Entry 17, Table 1). The first two (Entries 12 and 13, Table 1), as well as 5′-GACAGG-3′ (Entry 14, Table 1) and 5′-GACUGG-3′ (Entry 16, Table 1), were investigated in accordance with the studies of Chen et al.,^7^ in which the authors experimentally measured the affinities of sequences that also shared GACG, G-m^6^A-CG, GACG, G-m^6^A-CA, GACU, and G-m^6^A-CU motif-containing sequences in complex with RRM3. Our simulations in this section investigated RNA binding to the individual RRM3 domain, including protein residues resolved from the X-ray studies performed by Chen et al.^7^ The particular sequences investigated by us enabled a comparison with experimental studies,^7^ and biophysical understanding through the quantification of residue-nucleotide pairwise interaction free energies from our simulations. Furthermore, the last four sequences (Entry 14–17, Table 1) were investigated due to the abundance of such sequence motifs in pull-down assays by Choi et al.^4^ associated with RBM45 recognition properties; particularly, the m^6^A modification can be written by methyltransferases METTL3-METTL14 ^22^ within GAC­(A/U) motifs. Importantly, these studies were performed to broaden our understanding of the findings provided by experimental studies^4,7^ on the lack of preferential binding of m^6^A over adenine by the individual RRM3 domain, outside the context of the full-length RBM45 protein.

5′-GACGGG-3′ (Entry 12, Table 1) was slightly more favorable (∼0.2 kcal/mol) than 5′-G-m^6^A-CGGG-3′ (Entry 13, Table 1), which, despite the large standard deviation in the computed values, complies with the corresponding experimental binding free energy difference (∼0.2 kcal/mol) deducted from experimentally measured affinities of GACG and G-m^6^A-CG containing sequences.^7^ In contrast to 5′-GACGGG-3′ (Entry 12, Table 1) and 5′-G-m^6^A-CGGG-3′ (Entry 13, Table 1), which possessed overall favorable binding to the individual RRM3 domain, the binding of 5′-GACAGG-3′ (Entry 14, Table 1), 5′-G-m^6^A-CAGG-3′ (Entry 15, Table 1), 5′-GACUGG-3′ (Entry 16, Table 1), as well as 5′-G-m^6^A-CUGG-3′ (Entry 17, Table 1), was significantly less favorable (Figure 1C). These findings are consistent (e.g., follow the same trend) with experimental studies performed for sequences containing GACA and GACU motifs.^7^ The experimental binding free energy difference deducted from experimentally measured affinities^7^ for GACU vs GACG and for GACA vs GACG containing sequences are ∼0.3 and ∼0.7 kcal/mol, respectively, while our corresponding computationally predicted values are ∼1.0 and ∼1.8 kcal/mol, respectively; thus, our computations overestimate the differences deducted in experiments.

Overall, our studies showed that guanine at the fourth position resulted in significantly more favorable binding free energy to either uridine or adenine, with uridine being slightly more favorable than adenine. Interestingly, this additionally complies with experiments showing that the favorability of guanine is significantly higher than that of uridine and adenine, with adenine being the least favorable.^7^ In accordance with this, the interaction free energy per nucleotide of guanine at position 4 is significantly more favorable compared to that of uridine or adenine (Figure S17A, S17C, S17E). Substitution of adenine at position 4 resulted in weakening of polar interactions between C3:Y422, A4:R393, A4:E420, and A4:K433, as well as weakening of non-polar interactions between A4:Y422 (Figures 2C, 5A,B, Table 4, Table S4B, Figure S18A). Substitution of uridine at position 4 resulted in weakening of the polar interactions between C3:Y422, U4:R393, U4:E420, and U4:K433, as well as weakening of the non-polar interactions between U4:Y422 (Figures 2C, 5C,D, Table 4, Table S4B, Figure S18B). The favorability of guanine compared to adenine or uridine at position 4 was primarily attributed to the hydrogen bond formed between E420 and stacking with Y422, which was weakened or lost in particular runs investigating adenine or uridine substituted at this position. These findings are supported by the polar and non-polar interaction free energy calculations (Figure 2C, 5A,C, Table S4B, Figures S3, S18A, S18B). Compared to adenine and uridine, the polar interaction between A4:K433 was lost, making adenine at position 4 worse than uridine at position 4 (Figure 5A,D, Table 4, Table S4B, Figure S18). The overall weakening of interactions between adenine and uridine at position 4 with the protein is shown in Figure 5, where panels A and C show hydrogen bonds that were weakened or lost in the context of a stable conformation, and panels B and D show hydrogen bonds that were weakened or lost when the conformation started to change toward destabilization. Polar interactions between A4:R393, A4:E420, and A4:K433 were weakened when comparing 5′-G-m^6^A-CAGG-3′ to 5′-G-m^6^A-CGGG-3′ (Figures 2F, 5E, Table 4, Table S4B, Figures S6A and S19). Additionally, polar interactions between U4:R393, U4:E420, and U4:K433 were weakened when comparing 5′-G m^6^A-CUGG-3′ to 5′-G-m^6^A-CGGG-3′ (Figures 2F, 5F, Table 4, Table S4, Figures S6A and S20). The interaction free energy per nucleotide of uridine or adenine at position 4 is more unfavorable compared to that of guanine (Figures S17B, S17D, S17F) in accordance with their binding free energies (Figure 1C). Overall, we observed a significant tendency for destabilization when uridine or adenine was present at position 4 compared to guanine, irrespective of the presence of adenine or m^6^A at position 2. This was due to the inadequate ability of uridine or adenine at position 4 to “anchor” sufficiently well within the particular binding pocket of the RRM3 domain. Destabilization, reflecting a dynamical behavior, is also correlated with higher conformational variability, as reflected by the RMSD calculations (Table S1B).

**Table 4:** Summary of Intermolecular Polar and Non-Polar Interaction Free Energies between the Selected Nucleotides G-A/m^6^A-C-A/U and RBM45 Associated with Simulations at which RNA Binds to Individual RRM3 Domain (Entries 12–17), and at which the RNA Binds to RRM3 in the Context of the Full-Length Protein, and at Which Additional Residues of Other Domains Participate in Binding (Entries 18–21)^a^

**nucleotide:RBM45 residues (polar and non-polar interaction free energies)**	
**5′-GACGGG-3′ (entry 12)**	**5′-GACAGG-3′ (entry 14)**
Cyt3:Y422 (−5.5, −1.2)	Cyt3:Y422 (−2.6, −0.7)
Gua4:R393 (−2.7, −0.4)	Ade4:R393 (−0.2, −0.1)
Gua4:E420 (−16.6, −0.1)	Ade4:E420 (−0.1, −0.2)
Gua4:Y422 (−0.9, −8.4)	Ade4:Y422 (−0.0, −1.1)
Gua4:K433 (−2.6, −1.3)	Ade4:K433 (−0.2, −0.2)
**5′-GACGGG-3′ (entry 12)**	**5′-GACUGG-3′ (entry 16)**
Cyt3:Y422 (−5.5, −1.2)	Cyt3:Y422 (−2.1, −0.6)
Gua4:R393 (−2.7, −0.4)	Ura4:R393 (−0.3, −0.1)
Gua4:E420 (−16.6, −0.1)	Ura4:E420 (−0.2, −0.2)
Gua4:K433 (−2.6, −1.3)	Ura4:K433 (−1.5, −0.4)
**5′-G-m** ^**6**^ **A-CGGG-3′ (entry 13)**	**5′-G-m** ^**6**^ **A-CAGG-3′ (entry 15)**
Gua4:R393 (−2.1, −0.1)	Ade4:R393 (−0.5, −0.2)
Gua4:E420 (−1.7, −0.5)	Ade4:E420 (−3.0, −0.2)
Gua4:K433 (−3.6, −0.5)	Ade4:K433 (0.4, −0.7)
**5′-G-m** ^**6**^ **A-CGGG-3′ (entry 13)**	**5′-G-m** ^**6**^ **A-CUGG-3′ (entry 17)**
Gua4:R393 (−2.1, −0.1)	Ura4:R393 (−0.4, −0.1)
Gua4:E420 (−1.7, −0.5)	Ura4:E420 (0.5, −0.3)
Gua4:K433 (−3.6, −0.5)	Ura4:K433 (−3.0, −0.4)
**5′-GACAGG-3′ (entry 18)**	**5′-G-m** ^**6**^ **A-CAGG-3′ (entry 19)**
Gua1:R466 (−4.7, −2.7)	Gua1:R466 (−6.2, −1.9)
Gua1:N470 (−1.3, −0.9)	Gua1:N470 (−1.6, −1.5)
Gua1:R472 (−1.4, −0.4)	Gua1:R472 (−8.2, −5.6)
Gua1:Q473 (0.1, −0.1)	Gua1:Q473 (−1.5, −1.6)
Gua1:R474 (−0.8, −0.2)	Gua1:R474 (−3.3, −1.1)
Ade2:R393 (−5.8, −0.6)	m^6^A 2:R393 (−1.5, −0.2)
Ade2:F395 (−0.3, −5.0)	m^6^A 2:F395 (−0.5, −4.4)
Ade2:V424 (−0.1, −0.4)	m^6^A 2:V424 (−0.2, −0.9)
Ade2:K427 (−2.6, −0.5)	m^6^A 2:K427 (−6.4, −0.9)
Ade2:Y431 (−1.1, −1.5)	m^6^A 2:Y431 (−2.6, −1.4)
Ade2:M460 (0.4, −1.0)	m^6^A 2:M460 (0.2, −1.0)
Ade2:D463 (−0.2, −3.0)	m^6^A 2:D463 (−0.2, −1.3)
Ade2:R466 (−13.9, −4.8)	m^6^A 2:R466 (−5.5, −2.4)
Cyt3:K471 (−3.4, −1.2)	Cyt3:K471 (−5.7, −0.6)
Cyt3:R472 (−4.2, −0.7)	Cyt3:R472 (−10.5, −2.6)
Cyt3:R474 (−2.9, −0.7)	Cyt3:R474 (−4.2, −0.7)
Ade4:R472 (−1.7, −0.7)	Ade4:R472 (−9.8, −1.0)
Ade4:R474 (−13.3, −0.9)	Ade4:R474 (−21.0, −2.3)
Gua5:R472 (−0.7, −1.1)	Gua5:R472 (−2.1, −0.5)
Gua5:R474 (−10.0, −5.1)	Gua5:R474 (−15.5, −9.5)
Gua5:T475 (−5.1, −1.9)	Gua5:T475 (−8.3, −3.6)
Gua6:E198 (−5.9, −0.9)	Gua6:E198 (−12.4, −0.7)
Gua6:Q202 (−2.5, −3.0)	Gua6:Q202 (−4.4, −3.5)
**5′-GACUGG-3′ (entry 20)**	**5′-G-m** ^**6**^ **A-CUGG-3′ (entry 21)**
Gua1:K427 (−1.1, −0.6)	Gua1:K427 (−5.7, −1.6)
Gua1:R466 (−1.1, −1.5)	Gua1:R466 (−4.2, −1.7)
Ade2:F395 (−0.1, −5.2)	m^6^A 2:F395 (−0.9, −6.6)
Ade2:V424 (−0.7, −1.5)	m^6^A 2:V424 (−0.4, −1.8)
Ade2:K427 (−5.6, −1.2)	m^6^A 2:K427 (−8.1, −1.6)
Ade2:M460 (0.3, −0.7)	m^6^A 2:M460 (0.3, −1.3)
Ade2:D463 (−1.0, −2.6)	m^6^A 2:D463 (−2.5, −1.8)
Ura4:S425 (−0.8, −1.4)	Ura4:S425 (−2.7, −1.3)
Ura4:R471 (−1.2, −0.7)	Ura4:R471 (−2.4, −0.7)
Ura4:R474 (−14.4, −2.3)	Ura4:R474 (−19.5, −2.2)
Gua5:R472 (−0.2, −0.4)	Gua5:R472 (−2.3, −1.2)
Gua5:R474 (−13.2, −5.4)	Gua5:R474 (−20.9, −10.5)
Gua5:R475 (−3.0, −1.6)	Gua5:R475 (−9.1, −3.9)
Gua5:Y476 (0.7, −1.8)	Gua5:Y476 (−2.0, −2.5)
Gua6:R198 (−7.1, −0.9)	Gua6:R198 (−9.6, −0.9)
Gua6:Q202 (−3.8, −3.8)	Gua6:Q202 (−4.4, −3.8)

a The intermolecular polar and non-polar interaction free energies correspond to the average values (kcal/mol).

**Figure 5:**
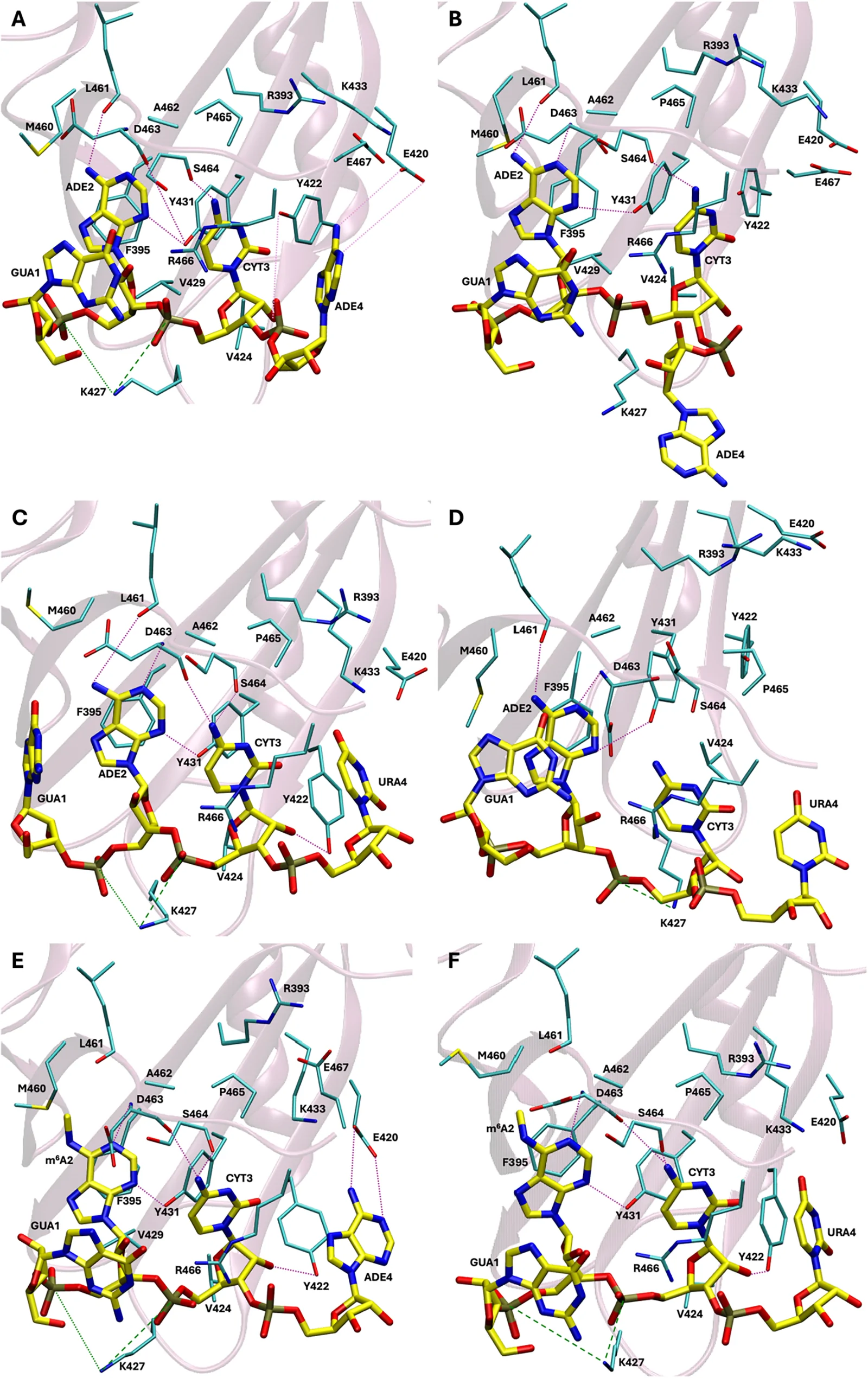
Simulation snapshots of RBM45 of GACA, G-m^6^A-CA, GACU, and G-m^6^A-CU motifs bound to the RRM3 domain. Interactions between the selected nucleotides of the motifs and nearby protein residues in the simulation snapshots are shown. Hydrogen bonds are shown by purple dashed lines; hydrogen bonds that occur within the simulation but are not present in the selected snapshot are shown by pink dashed lines. coulombic interactions are shown by green dashed lines, and weaker coulombic interactions are shown by less dense green dashed lines. Particular weak coulombic interactions are not indicated by dashed lines for clarity. A full list of hydrogen bonds and coulombic interactions is reported in Table S4B. (A–B) RBM45 in complex with 5′-GACAGG-3′ (Entry 14). (A) represents the system at the beginning of the simulation trajectory, where the RNA strand is well bound to the binding pocket. (B) represents the system after 17 ns, where the RNA strand starts to deviate from the binding pocket in a particular trajectory. (C–D) RBM45 in complex with 5′-GACUGG-3′ (Entry 16). (C) represents the system at the beginning of the simulation trajectory, where the RNA strand is well bound to the binding pocket. (D) represents the system after 7 ns, where the RNA strand starts to deviate from the binding pocket in a particular trajectory. (E–F) RBM45 in complex with 5′-G-m^6^A-CAGG-3′ (Entry 15) and 5′-G-m^6^A-CUGG-3′ (Entry 17), respectively. RRM3 is shown in magenta as a new cartoon representation.

Due to the high conformational variability observed in the simulations of GACA and GACU motifs with or without m^6^A modifications, we used clustering analysis to delineate similarities and differences between the conformational states of all strands (GACG, GACA, and GACU motifs with or without m^6^A modifications) simulated in complex with the RRM3 domain. In this analysis, the backbone atoms of different RNA strands were combined to identify how different RNA strands can be structurally clustered (together in the same cluster or differently in different clusters (Figure S21). Clusters 1, 2, 5, 6, 7, 8, and 9 involve binding conformations of the RNA backbone (considering nucleotides 2–5) within ∼2.0–3.8 Å (Figure S21B, S21C) of the initial conformation, referred to as the refined structure. These conformations were adopted by all RNA sequences investigated in complex with RRM3. On the contrary, clusters 3, 4, 10, and 11 involve binding conformations that are more distant from the initial conformation, ∼5.2–19 Å (Figure S21B, S21D), and were loosely bound. These were mostly adopted by both unmodified and m^6^A-modified 5′-GACAGG-3′ and 5′-GACUGG-3′. Larger RMSD values were recorded for RNA strands for unmodified and m^6^A-modified 5′-GACAGG-3′ and 5′-GACUGG-3′ (Entries 14–17, Table 1) compared to unmodified and m^6^A-modified 5′-GACGGG-3′ (Entries 12 and 13, Table 1), reflecting their tendency to adopt different loosely bound conformations (Table S1B). Also, the overall average and the standard deviation RMSD values for RRM3 were notably larger for unmodified and m^6^A-modified GACA and GACU-containing sequences compared to GACG. Additionally, these correspond to generally larger RMSD values for unmodified and m^6^A-modified GACG, GACA, and GACU-containing sequences binding RRM1–RRM2 (Tables S1–S3). This is associated with the higher instability of GACA and GACU (unmodified and m^6^A-modified) containing sequences binding to RRM3 (Figure 5), which is also reflected by their corresponding less favorable binding free energies (Figure 1C). These could be, at least in part, attributed to the less favorable interactions of uridine and adenine at position 4, both in unmodified and m^6^A-modified RNAs, with RRM3. Overall, these findings highlight the challenges to study the m^6^A binding pocket within the RRM3 domain in the absence of the full-length RBM45 protein, and highlight the importance of studying the binding of RNA to RRM3 in the context of full-length RBM45, where other domains may potentially enhance RNA stabilization.

The aforementioned analysis is based on the average values of the triplicate simulations. A complete list of hydrogen bonds and coulombic interactions, both associated with polar interactions, formed in the aforementioned complexes, is shown in Table S4B. The strengths of favorable (Δ*G* < 0) residue–nucleotide pairwise interaction free energies, decomposed into polar and non-polar components, are shown in Figures S18A, S19A, S20A, while the nucleotide interaction free energies are shown in Figure S17. Also, a comparison between the unmodified and m^6^A-modified sequences is shown in Figures S18B, S19B and S20B.

### GACA, G-m^6^A-CA, GACU, and G-m^6^A-CU in Complex with RRM3 in the Context of the Full-Length RBM45 Protein Show Preferential Binding of m^6^A over Adenine

Prompted by our results and those of Choi et al., which highlight the importance of RRM3, suggest that the C-terminal RBDs of RBM45, comprising the HOA and RRM3 domains, can work cooperatively to recognize m^6^A.^4^ We focused on investigating RNA binding to RRM3 in the context of the full-length protein. In our studies above, the concurrent inclusion of both RRM1 and RRM2 conferred a high degree of stability to RNA in complex with either of the two. However, the absence of any other domains in the case of RRM3 could significantly affect the binding and stability of the RNA. Thus, motivated by our findings, we hypothesized that investigating RNA binding to RRM3 in the context of additional domains present in the entire protein could facilitate and improve the binding of RNA containing GACA and GACU motifs and potentially help us elucidate the preferential binding of modified over unmodified sequences.

#### Binding of RNA to RRM3 in the Context of the Full-Length RBM45 Protein Was Accompanied by Synergism of Other Domains

The initial simulation structure of RNA in complex with RRM3 in the context of the full-length RBM45 protein (representing a chimeric construct; see the Methods section) prior to simulation refinement is presented in Figure 6A. The refined structure used as a starting point in our simulations is presented in Figure 6B, and a simulation snapshot extracted from the run with the lowest average binding free energy starting from the refined structure is presented in Figure 6C. Noteworthy differences were observed between Figure 6A,B, suggesting that RNA may partially contribute to the order and arrangement of the RRMs and the HOA domain. During the entire duration of the simulations, the conformations of the four RNA–protein complexes remained within ∼3–6 Å during the last 200 ns with respect to the refined structure, which was used as a common starting point (Figure S22A–S22D). Of particular interest was that the RNA bound to RRM3 was in proximity to the preceding residues stemming from the HOA domain, while succeeding residues beyond RRM3 in the C-terminal domain interacted with both RNA and HOA. This is reflected in the RMSD calculations in Table S1C. Two types of RMSD calculations were performed: in the first calculation, each trajectory was aligned with respect to the backbone of RRM3 and RNA, while in the second calculation, each trajectory was aligned with respect to the backbone of the full-length protein and RNA. Both were performed with respect to the corresponding average simulation conformations. The results of the first calculation showed a relatively high level of structural integrity of RRM3 and the bound RNA, in contrast to the case in which RNA was simulated in complex with the individual RRM3 domain. This indicates that RNA remains more stably bound to RRM3 when the full-length protein is included for both unmodified and modified RNA sequences. This was an outcome of the fact that nucleotides 4, 5, and 6 improve binding by adjusting toward more stable binding by interacting with additional domains beyond RRM3. The results of the second calculation showed a relatively high stability of the entire simulated complex comprising RNA and the full-length protein for both unmodified and modified RNA sequences within the last 200 ns of each trajectory, which was considered for further investigation. Both N-terminal and C-terminal domains show relatively higher deviations compared to the other RRM domains and linkers. Interestingly, the C-terminal domain was associated with higher stability for both m^6^A-containing complexes (Entries 19 and 21, Table 1) and the corresponding unmodified complexes (Entries 18 and 20, Table 1). This was also reflected in the lower RMSD values calculated for the RNA strand in the same calculation, and was an outcome of the fact that m^6^A contributed to enhanced stabilization (Table S1C) of interactions formed between nucleotides 4, 5, and 6 with the C-terminal domain and the linkers, as well as improved stabilization of an intraprotein interaction formed by these domains, which is described in more detail below.

**Figure 6:**
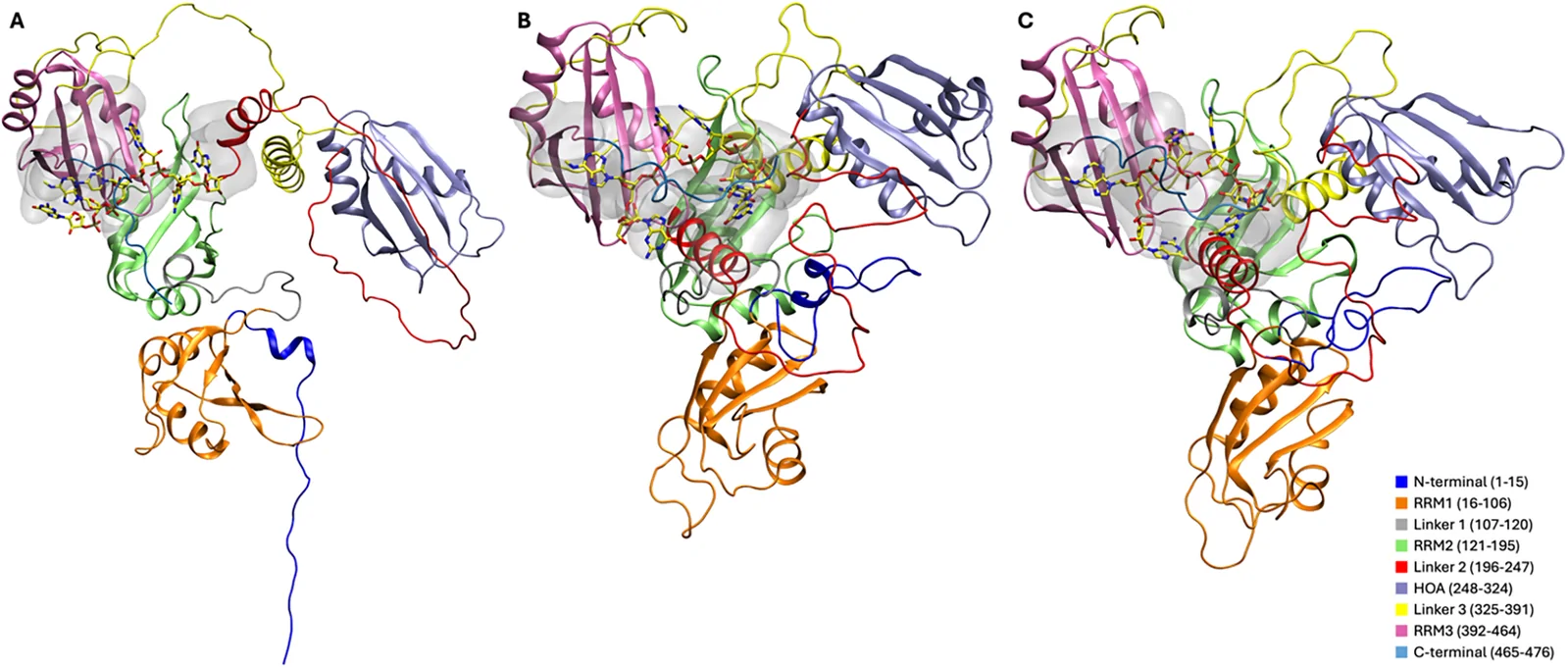
Structures of 5′-G-m^6^A-CAGG-3′ in complex with RRM3 in the context of the full-length RBM45 protein. The surfaces of the protein residues within 3.5 Å of the RNA strand are shown. (A) Chimeric construct of the AlphaFold Database model^8,9^ and PDB: 8WQ5 ^7^ after energy minimization and prior to simulation. (B) The minimum binding free energy snapshot identified from the refinement simulation runs is defined as the refined structure. (C) Simulation snapshot extracted from the run with the lowest average binding free energy starting from a refined structure. Domains are shown in a new cartoon representation, and the domain-color correspondence is shown below panel C.

Unlike guanine at position 1 in complex with the individual RRM3 domain, the first guanine in complex with RRM3 in the context of the full-length RBM45 protein was still intercalated between different residues, but showed improved interactions overall with residues nearby in all unmodified and m^6^A-modified GACA and GACU motifs. The second and third nucleotides in all unmodified and m^6^A-modified GACA and GACU motifs behaved in a similar manner and maintained their original binding pockets and interactions with RRM3, which were reminiscent of those depicted above for GACG motifs in complex with the individual RRM3 domain. This can be attributed to the stable binding of adenine or uridine at position 4 of GACA or GACU in the context of the full-length protein, in contrast to the unstable binding observed in the context of an individual RRM3 domain. With the presence of extra residues beyond RRM3 in the full-length protein, an adjustment of adenine and uridine at position 4 and primarily guanine at positions 5 and 6 was observed, leading to additional interactions with the C-terminal domain, as well as with the linkers between RRM2-HOA and HOA-RRM3.

#### Simulations Suggest Preferential Binding of G-m^6^A-C­(A/U) over GAC­(A/U) in the Context of the Full-Length RBM45 Protein

In simulations of 5′-G-m^6^A-CAGG-3′ in complex with RRM3 in the context of the full-length RBM45 protein (Entry 19, Table 1), the nucleobase of m^6^A at position 2 was primarily within the RRM3 binding pocket, comprising residues R393, F395, V429, Y431, M460, A462, and D463, while its backbone phosphate group formed coulombic interactions with K427 as well as with R466 and R471 from the C-terminal domain (Figure 7A, Table S5, Figure S23A). The nucleobase of cytosine at position 3 was within the RRM3 binding pocket, comprising residues R393, Y422, V424, Y431, and P465, while its backbone phosphate group formed weaker coulombic interactions with R393 and K427, as well as R466, K471, R472, and R474 from the C-terminal domain (Figure 7A, Table S5, Figure S23A). The nucleobase of adenine at position 4 was within the RRM3 binding pocket, comprising residues Y422, V424, and S425, while its backbone phosphate group formed coulombic interactions with R472 and R474, as well as a weaker coulombic interaction with K471 from the C-terminal domain (Figure 7B, Table S5, Figure S23A). The nucleobase of guanine at position 5 was within a binding pocket, comprising residues S425 of RRM3, R209, Q210, and G242 of the linker between RRM2-HOA (196–247) and Q473, T475, and Y476 of the C-terminal domain, while its backbone phosphate group formed coulombic interactions with R241 of the linker between RRM2-HOA and R472, R474 from the C-terminal domain (Figure 7B, Table S5, Figure S23A). The nucleobase of guanine at position 6 primarily interacted with nearby residues E198, Q202, G242, Q243, A245, and I246 of the linker between RRM2-HOA and D323 of the HOA, T329, D330, and R333 of the linker between HOA-RRM3, as well as S425 and G426 of RRM3. Its backbone phosphate group formed weaker coulombic interactions with R209 of the linker between RRM2-HOA and R333 of the linker between HOA-RRM3 (Figure 7B, Table S5, Figure S23A). The stabilization of the entire RNA in the presence of m^6^A was also complemented by further formation of intraprotein interactions involving the C-terminus and the linker between RRM2-HOA. In particular instances, the positively charged R241 tended to face the negatively charged Y476 at the C-terminal domain, providing additional stability between the linker, RRM2-HOA, and the C-terminal domain (Figure S24).

**Figure 7:**
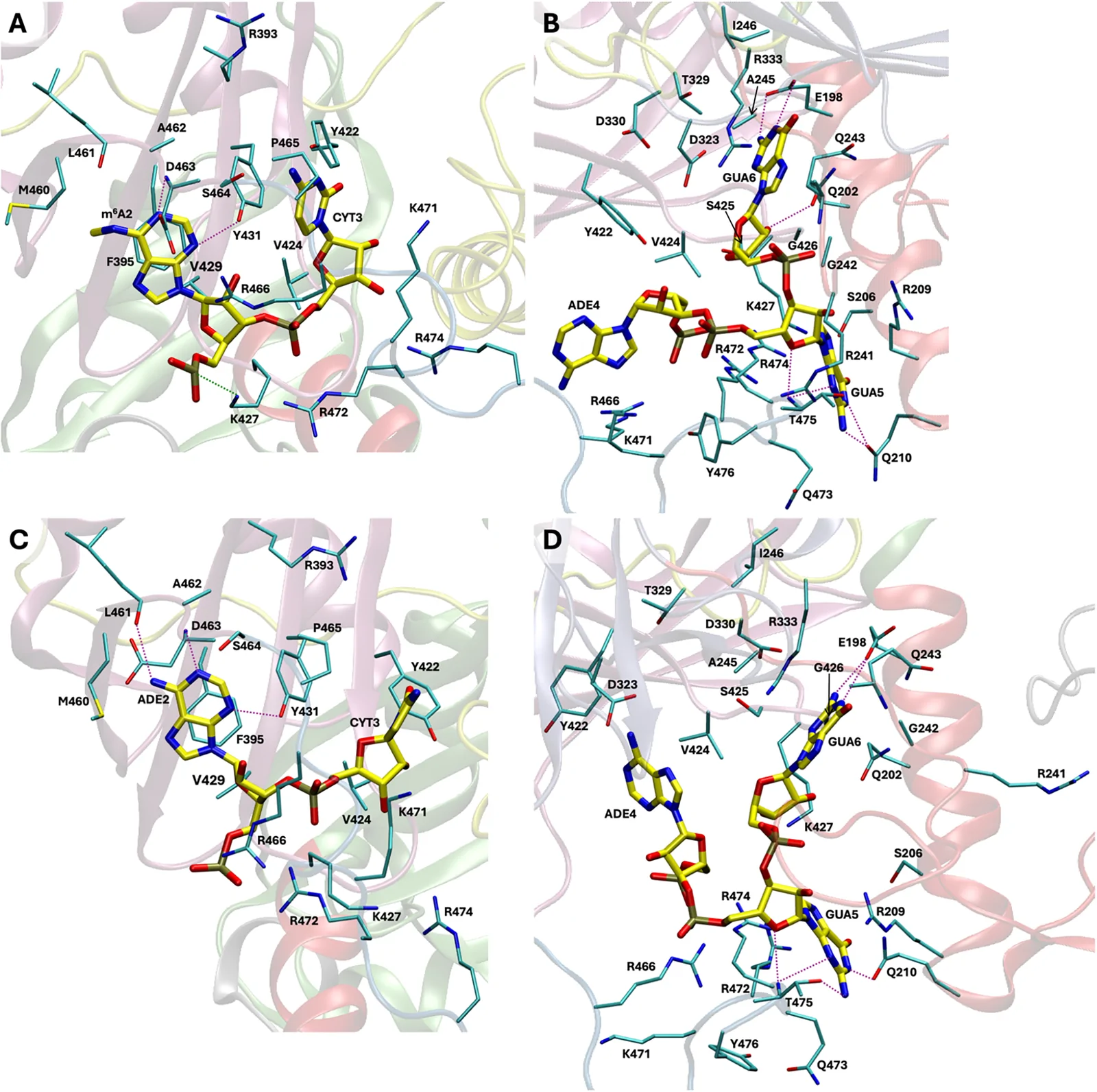
Simulation snapshots of RBM45, where the RNA strand is bound to the RRM3 domain in the context of the full-length RBM45 protein. Interactions between the selected nucleotides of the motifs and nearby protein residues in the simulation snapshots are shown. Hydrogen bonds are shown by purple dashed lines, coulombic interactions are shown by green dashed lines, and weaker coulombic interactions are shown by less dense green dashed lines. Particular weak coulombic interactions are omitted from the figures for clarity. A full list of hydrogen bonds and coulombic interactions is reported in Table S5. (A–B) 5′-G-m^6^A-CAGG-3′ (Entry 19) in complex with RBM45. (A) Interactions between nucleotides 2 and 3 and nearby protein residues. (B) Interactions between nucleotides 4 and 6 and nearby protein residues are shown. (C–D) 5′-GACAGG-3′ (Entry 18) in complex with RBM45. (C) Interactions between nucleotides 2 and 3 and nearby protein residues. (D) Interactions between nucleotides 4 and 6 and nearby protein residues. Domains are shown in a new cartoon representation, and the domain-color correspondence is the same as that presented in Figure 6.

5′-G-m^6^A-CAGG-3′ and 5′-GACAGG-3′ shared similar binding modes in complex with RRM3 in the context of the full-length RBM45 within the simulations (Figure 7, Table S5, Figures S23 and S25). Interestingly, in addition to the interactions formed with RRM3, in both cases, RNAs interacted with residues of the linker between RRM2-HOA (196–247), the end of HOA, and the linker between HOA and RRM3 (325–391), as well as the C-terminal domain. The first three nucleotides of 5′-G-m^6^A-CAGG-3′ and 5′-GACAGG-3′ interacted with both RRM3 and the C-terminal domains, while the last three nucleotides interacted with RRM3 and the C-terminal domain, as well as the linker between RRM2-HOA (196–247), HOA, and the linker between HOA-RRM3 (325–391) (Figure 7, Table S5, Figures S23 and S25). Despite the similarities between the two complexes, the two systems differed in key aspects. As a result, the binding free energy for 5′-G-m^6^A-CAGG-3′ (Entry 19, Table 1) was notably more favorable than that for 5′-GACAGG-3′ (Entry 18, Table 1) (Figure 1C). The six runs performed per system allowed us to verify a statistically significant difference between the two (*p* = 0.03) according to an unpaired two-tailed Welch’s *t* test (*n* = 6 per group) calculated using the average values of the sextuplicate runs. Specifically, when comparing 5′-G-m^6^A-CAGG-3′ and 5′-GACAGG-3′, m^6^A possessed stronger polar interactions with K427 and Y431; the former was associated with an improved coulombic interaction between the backbone phosphate of m^6^A, while the latter was associated with a higher occupancy hydrogen bond (Figure 7A,C, Table 4, Table S5, Figures S23 and S25). While polar interactions of m^6^A at position 2 were weakened with R393, L461, A462, R466, K471, and R474, compared to adenine at position 2, the aforementioned improved interactions of m^6^A at position 2 of 5′-G-m^6^A-CAGG-3′ came along with additional improved interactions of guanine at position 1, including more favorable polar interactions with the C-terminal R466 and N470, and occasionally with R472, Q473, and R474. The interactions involving arginine residues comprise coulombic interactions (Figure 7A,7C, Table 4, Table S5, Figures S23 and S25). Cytosine at position 3 also possessed a combination of improved polar interactions with K471, R472, and R474, non-polar interactions with Y422 (Figure 7A,C, Table 4, Table S5, Figures S23 and S25), and weakened interactions with R393, Y422, K427, and R466. Notably, the per-nucleotide interaction free energies for m^6^A/adenine at position 2 and cytosine at position 3 were less favorable in G-m^6^A-CA over GACA. Yet, they were more favorable for guanine at positions 1, 5, and 6, and to a lesser extent, adenine at position 4 (Figure S26A and S26B), which led to better structural stabilization of the RNA (Table S1C). Adenine at position 4 formed stronger polar interactions with S425, R472, and R474, and non-polar interactions with the apolar moiety of R474. Importantly, guanine at position 5 also formed stronger polar interactions with S425, R472, R474, and T475, as well as non-polar interactions with the apolar moieties of Q473, R474, and T475 in 5′-G-m^6^A-CAGG-3′ compared to 5′-GACAGG-3′ (Figure 7B,D, Table 4, Table S5, Figures S23 and S25). Guanine at position 6 formed stronger polar interactions with E198, Q202, Q243, A245, I246, D323, T329, and S425, as well as improved non-polar interactions with the apolar moieties of Q423 and R333 in 5′-G-m^6^A-CAGG-3′ compared to 5′-GACAGG-3′ (Figure 7B,D, Table 4, Table S5, Figures S23 and S25). Interactions formed by the last three nucleotides, primarily by the fifth and sixth nucleotides, with the C-terminal domain of the RBM45 protein or the linker between RRM2-HOA were overall more favorable in the case of m^6^A-modified RNA. The stabilization of the entire RNA–protein complex (Table S1C) was complemented by the formation, in particular instances, of an intraprotein coulombic interaction between C-terminal residue Y476 and R241, a residue within the linker between RRM2-HOA. The two residues were overall more frequently in closer proximity in the simulations of 5′-G-m^6^A-CAGG-3′ (Entry 19, Table 1) compared to 5′-GACAGG-3′ (Entry 18, Table 1) (Figure S24). The improved stabilization was also reflected in the RMSD calculations, where the average RMSD values of HOA and RRM3 domains and linkers between RRM2-HOA and HOA-RRM3 were overall lower with the presence of m^6^A (Table S1C)

The binding free energy of 5′-GACUGG-3′ (Entry 20, Table 1) was on par with 5′-GACAGG-3′ (Entry 18, Table 1) in complex with RRM3 in the context of the full-length RBM45 protein (Figure 1C), with the per-nucleotide interaction free energy of uridine being less favorable than that of adenine at position 4 (Figure S25B, 25D). The binding of 5′-GACUGG-3′ was similar to that of 5′-GACAGG-3′ (Figures S23B and S27, Table S5), with some differences including weaker polar interactions between U4:S425, R472, and R474 (Figures S23B and S27, Table 4, Table S5). The binding free energy for 5′-G-m^6^A-CUGG-3′ (Entry 21, Table 1) was notably more favorable than that of 5′-GACUGG-3′ (Entry 20, Table 1), which was in line with the preferential binding observed in experiments,^4^ and was analogous to the notably more favorable binding of 5′-G-m^6^A-CAGG-3′ to 5′-GACAGG-3′ described above.

The preferential binding of 5′-G-m^6^A-CUGG-3′ compared to its unmodified sequence can be attributed to the analogous factors noted above in the context of 5′-G-m^6^A-CAGG-3′ to 5′-GACAGG-3′. A comparison of per-nucleotide interactions of m^6^A with adenine at position 2 shows that in the case of G-m^6^A-CU, m^6^A possessed improved interactions over adenine to some extent, as reflected by per-nucleotide interaction free energies. Additionally, guanine at positions 1 and 5, and, to a lesser extent, uridine at position 4, possessed more favorable interaction free energies in 5′-G-m^6^A-CUGG-3′ compared to 5′-GACUGG-3′ (Figures S26C, S26D), which led to slightly better structural stabilization (Table S1C). Analogously to the comparison above for G-m^6^A-CA and GACA, m^6^A possessed improved non-polar interactions with F395 and the apolar moieties of R466 in 5′-G-m^6^A-CUGG-3′ compared to 5′-GACUGG-3′ (Figure 8A,C, Table 4, Table S5, Figures S28 and S29), while m^6^A formed stronger polar interactions with K427 and D463 (Figure 8A,C, Table 4, Table S5, Figures S28 and S29) compared to adenine. The improved interactions formed by m^6^A compared to adenine at position 2 of 5′-G-m^6^A-CUGG-3′ were in conjunction with the improved overall binding of guanine at position 1, including more favorable polar interactions with D203, K427, N428, and R466 (Table 4, Table S5, Figures S28 and S29). Cytosine at position 3 formed stronger polar interactions with Y422, Y431, K471, and R474, while it formed weaker non-polar interactions with the non-polar moiety of R472. Uridine at position 4 formed stronger polar and non-polar interactions with S425, K471, and R474, while it formed weaker polar interactions with K427, R472, and R474 in 5′-G-m^6^A-CUGG-3′ compared to 5′-GACUGG-3′. Importantly, guanine at position 5 also formed stronger polar interactions with Q210, R241, R472, R474, T475, and Y476, and non-polar interactions with R474 and T475, which outweighed weakened polar interactions with Y422 and S425 in 5′-G-m^6^A-CUGG-3′ compared to 5′-GACUGG-3′ (Figure 8B,D, Table 4, Table S5, Figures S28 and S29). Guanine at position 6 formed stronger polar interactions with E198 and Q202, as well as weakened polar interactions with R209 and R333 (Figure 8B,D, Table 4, Table S5, Figures S28 and S29).

**Figure 8:**
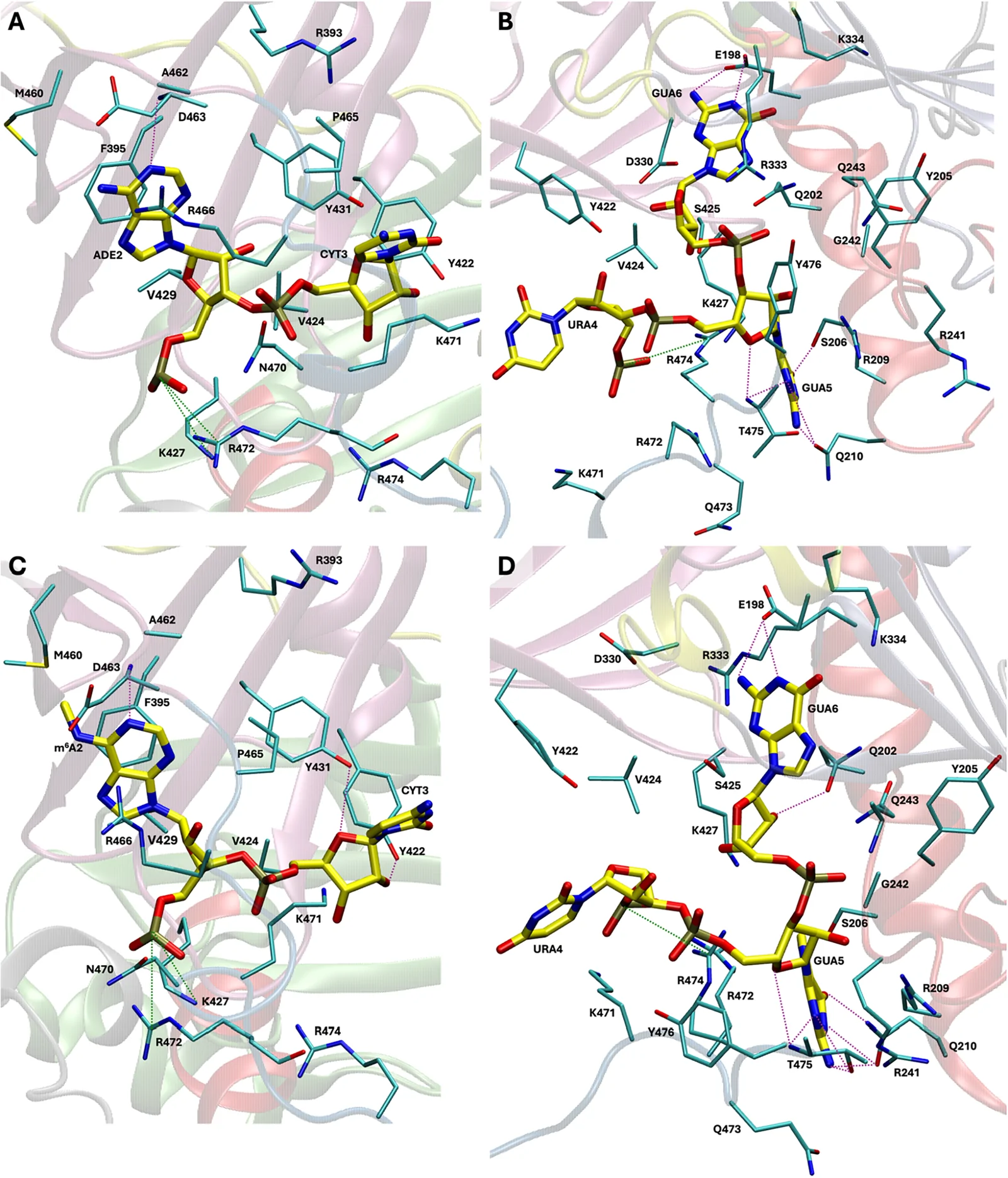
Simulation snapshots of RBM45, where the RNA strand is bound to the RRM3 domain in the context of the full-length RBM45 protein. Hydrogen bonds are shown by purple dashed lines, coulombic interactions are shown by green dashed lines, and weaker coulombic interactions are shown by less dense green dashed lines. Particular weak coulombic interactions are omitted from the figures for clarity. A full list of hydrogen bonds and coulombic interactions is reported in Table S5. (A–B) 5′-GACUGG-3′ (Entry 20) in complex with RBM45. (A) Interactions between nucleotides 2 and 3 and nearby protein residues. (B) Interactions between nucleotides 4 and 6 and nearby protein residues. (C–D) 5′-G-m^6^A-CUGG-3′ (Entry 21) in complex with RBM45. (C) Interactions between nucleotides 2 and 3 and nearby protein residues. (D) Interactions between nucleotides 4 and 6 and nearby protein residues. Domains are shown in a new cartoon representation, and the domain-color correspondence is the same as that presented in Figure 6.

Similar to simulations of 5′-G-m^6^A-CAGG-3′ (Entry 19, Table 1) and 5′-GACAGG-3′ (Entry 18, Table 1), the RNA interacted with the residues of the linker between RRM2-HOA (196–247), the end of HOA, and the linker between HOA-RRM3 (325–391), as well as the C-terminal domain in the simulations of 5′-G-m^6^A-CUGG-3′ (Entry 21, Table 1) and 5′-GACUGG-3′ (Entry 20, Table 1) in complex with RRM3 in the context of full-length RBM45. RNA contributed to the interactions between the C-terminal domain and the linker between RRM2-HOA (196–247). Particularly, the positively charged R241 was overall more frequently in closer proximity to the negatively charged Y476 at the C-terminal domain (Figure S30) in the case of 5′-G-m^6^A-CUGG-3′ (Entry 21, Table 1), providing additional stability between HOA and the C-terminal domain. In contrast to 5′-G-m^6^A-CAGG-3′ versus 5′-GACAGG-3′, in 5′-G-m^6^A-CUGG-3′, the interactions between m^6^A at position 2 were more favorable than those between adenine; thus, the methyl group provides primarily more favorable interactions (Figure S26) and, to a lesser extent, improved stability of the RNA strand compared to adenine in 5′-GACUGG-3′ (Table S1C). The improved interactions of m^6^A at position 2 of 5′-G-m^6^A-CUGG-3′ occurred in conjunction with the improvement of interactions formed by nucleotides 1, 5, and to a lesser extent, 4. This was accompanied by nucleotides 4, 5, and 6 adjusting toward the C-terminal domain and linkers between RRM2-HOA and HOA-RRM3, and was also complemented by the augmented formation of an intraprotein interaction among the C-terminal residue Y476 and R241, a residue within the linker between RRM2-HOA. Similar to what was discussed above for G-m^6^A-CA, the improved stabilization is also reflected in the RMSD calculations, where the average RMSD values of the HOA and RRM3 domains and linkers between RRM2-HOA and HOA-RRM3 are overall lower in the presence of m^6^A (Table S1C).

Overall, our simulations depict that both unmodified and m^6^A-modified oligos, adenine, or uridine nucleotides at position 4 no longer interact unfavorably with RRM3, as is the case when only RRM3 is present. This can be attributed to the improvement of specific interactions formed by m^6^A over adenine. The methyl group of m^6^A did not necessarily contribute directly to improved interactions in G-m^6^A-CA (Entry 19, Table 1) and G-m^6^A-CU (Entry 21, Table 1) compared to the GACA (Entry 18, Table 1) and GACU (Entry 20, Table 1) motifs in the context of the full-length protein. To examine the role of the extra methyl group in the stability of m^6^A vs adenine in the pocket, we calculated the interatomic distance between m^6^A/A (C4) and residue F395 (CG) within the simulation trajectories. According to visual inspection, the proximity of the nucleobase to F395 was associated with its stability in the pocket. Our results showed higher stability of the second nucleobase in both G-m^6^A-CA (5.2 ± 1.6 Å) and G-m^6^A-CU (4.4 ± 0.0 Å) motifs in complex with RRM3 in the context of the full-length protein compared to GACA (6.1 ± 3.0 Å) and GACU runs (5.3 ± 1.6 Å); the numbers in parentheses correspond to the average and standard deviation values of distances. The standard deviations reflect the fact that in minor cases, m^6^A or adenine became less stable or came out of the pocket, mostly in unmodified versus corresponding modified sequences (Figures S31 and S32), which can also be seen in the provided PDB structures (see below). In the modified cases, stability was directly accompanied by the extra methyl group of m^6^A being in proximity to the hydrophobic M460 and aromatic F395, providing evidence of how the methyl group directly facilitated improved stability of its nucleobase. A schematic molecular graphics representation summarizing the proposed mechanism, leading to G-m^6^A-CAGG preferential binding over adenine for GACAGG, is shown in Figure 9. The figure demonstrates the domain architecture and focuses on the key synergistic contacts from RRM3 and non-RRM3 domains stabilizing nucleotides, the role of the m^6^A methyl group in proximity to F395 and M460, as well as the intraprotein interactions among the C-terminal residue Y476 and R241, a residue within the linker between RRM2-HOA.

**Figure 9:**
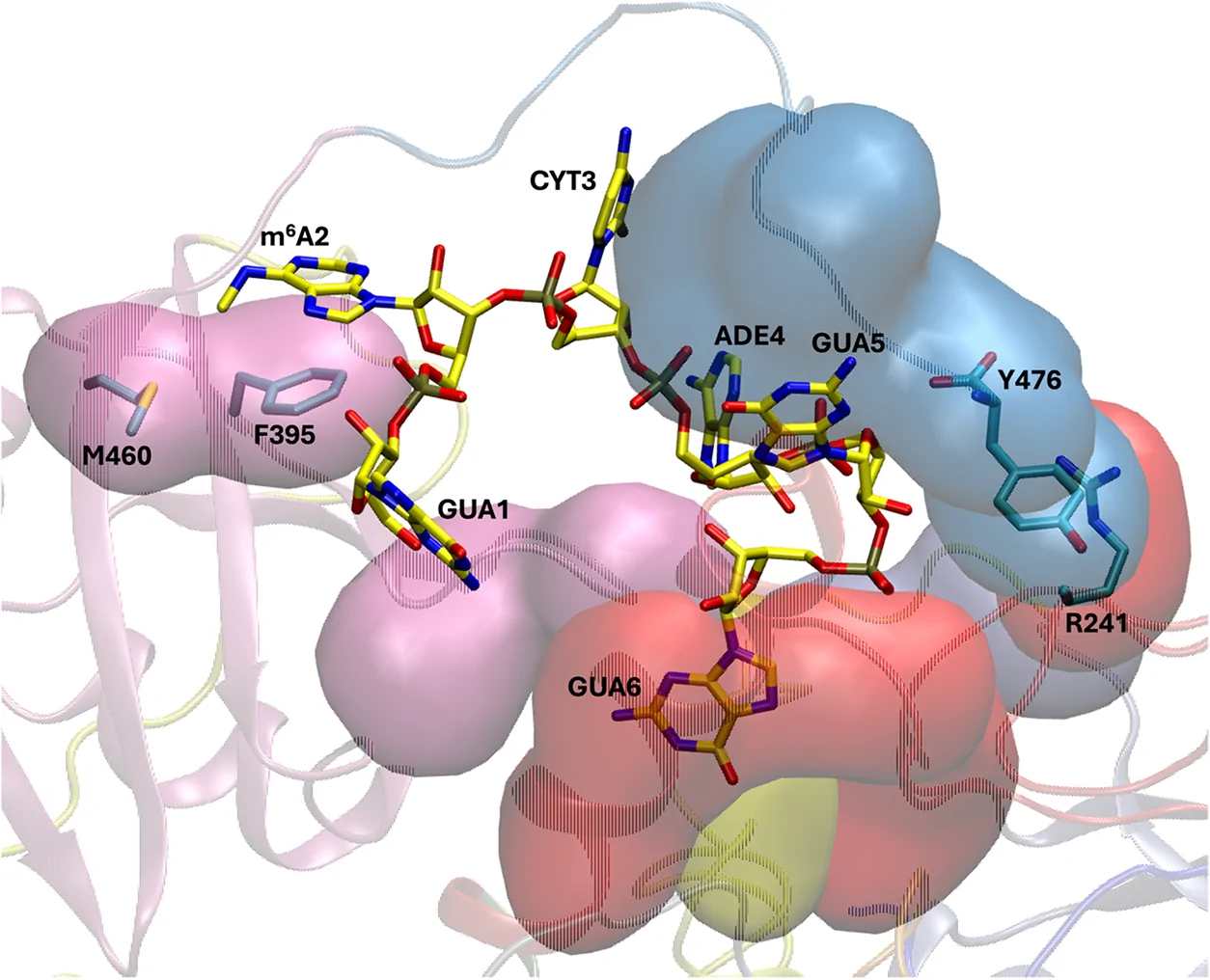
Simulation snapshot of 5′-G-m^6^A-CAGG-3′ (Entry 19) in complex with RRM3 in the context of full-length RBM45, highlighting the aspects that contribute to  m^6^A preferential binding. M460 and F395 are positioned in proximity to the methyl group of m^6^A, while Y476 and R241 were overall more frequently in closer proximity compared to the simulations of the unmodified motif 5′-GACAGG-3′ (Entry 18). The aforementioned residues are shown in licorice and transparent surface representations. Additionally, protein residues that exhibited stronger interaction free energy in pairs with nucleotides 4, 5, and 6, compared to the unmodified motif runs, are shown in transparent surface representation and are colored by the domain; the domain-color correspondence is the same as that presented in Figure 6. As for adenine at position 4, stronger interactions were observed with S425 (RRM3) and R472 and R474 (C-terminal). As for guanine at position 5, stronger interactions were observed with S425 (RRM3) and R472, Q473, R474, and T475 (C-terminus). As for guanine at position 6, stronger interactions were observed with E198, Q202, Q243, A245, and I246 (RRM2–HOA linker), D323, and T329 (HOA–RRM3 linker), and S425 (RRM3). The sum of the average polar and non-polar components was considered a criterion to identify pairs with stronger interaction free energies than those of the unmodified motif runs.

The aforementioned analysis is based on the average values of sextuplicate (G-m^6^A-CA, GACA) or triplicate (G-m^6^A-CU, GACU) simulation runs. A complete list of hydrogen bonds and coulombic interactions, both associated with polar interactions, formed in the aforementioned complexes is shown in Table S5. The strength of favorable (Δ*G* < 0) residue–nucleotide pairwise interaction free energies, decomposed into polar and non-polar components, are shown in Figures S23, S27, and S28, while per-nucleotide interaction free energies are shown in Figure S26. Also, a comparison between the unmodified and m^6^A-modified sequences is shown in Figures S25 and S29. Additionally, simulation snapshots corresponding to 60 snapshots, extracted every 20 ns from six trajectories of 5′-G-m^6^A-CAGG-3′, 5′-GACAGG-3′, and 30 snapshots, extracted every 20 ns from three trajectories of 5′-G-m^6^A-CUGG-3′, and 5′-GACUGG-3′, in complex with RRM3 in the context of the full-length protein, are provided in PDB format. In the PDBs, snapshots 1–10, 11–20, 21–30, 31–40, 41–50, and 51–60 correspond to the first, second, third, fourth, fifth, and sixth replicates, respectively, with 5′-G-m^6^A-CUGG-3′ and 5′-GACUGG-3′ comprising only three replicates.

## Conclusions

RBM45 has been observed to bind preferentially to m^6^A-containing RNAs,^4,7^ and thus, it could potentially mediate the regulation of RNA splicing and/or postsplicing events by m^6^A modification.^7^ Nevertheless, a single RRM domain does not exhibit any preference for m^6^A modification of short RNA fragments *in vitro*;^7,27^ this is in stark contrast to the well-studied YTH binding proteins, which contain specific m^6^A-binding domains.^64^ Proteins with three or more RRM domains show a great variety in domain conformation, arrangement, and interaction between RRMs.^65^ Here, we used a combination of computational studies, primarily MD simulations, to study the recognition properties of RBM45 by RNAs. Computational studies have increasingly been used to study RNA conformations and RNA interactions with proteins or other molecules, including investigations of RNA modifications.^33,57,58,60,64,66–75^

Our computational studies provided insights into the binding mechanism of different RRM domains of RBM45 with a series of RNA sequences, with or without m^6^A, and highlighted the importance of investigating the full-length RBM45 protein. We comply with previous findings that m^6^A and adenine are approximately equally favorable binders to RRM1–RRM2 ^27^ as well as RRM3,^7^ individually, in the context of GACG motifs. Regarding RRM1–RRM2, our simulations suggest that the slight favorability of unmodified RNA over m^6^A-modified RNA in the context of GACG motifs could likely be driven mostly by the RRM2 domain. For the RRM3 domain, our simulations are not only in agreement with ref^7^ but, importantly, they show for the first time why GACG is more favorable to GACU and even more favorable to GACA, identifying which interactions lead to destabilization of uridine and adenine at position 4 in complex with RRM3. This enabled us to provide, to the best of our knowledge, the first atomistic structures of RNA, comprising GACA and GACU motifs, in complex with RRM3 in the context of full-length RBM45, and uncover the preferential binding properties of m^6^A compared to adenine. In our simulations, we observed that regions serving as linkers between the RRM domains and the HOA domains could adopt stable conformations in the presence of RNA. This can be attributed to the fact that the linkers and C-terminal domains directly interact with RNA strands. Nucleotide 4 and primarily nucleotides at positions 5 and 6 participate in the interaction with the C-terminal domain, as well as the linker between RRM2-HOA and HOA-RRM3 in G-m^6^A-CA, GACA, G-m^6^A-CU, and GACU-containing sequences. Thus, we suggest that the unfavorable nature of GACA and GACU for RRM3 does not hold when RRM3 is in the context of the full-length protein. Our simulations suggest that protein residues succeeding and proceeding beyond RRM3 can synergistically cooperate for m^6^A preferential binding over adenine. The presence of the methyl group of m^6^A provides further stability to its nucleobase and improves particular interactions in m^6^A (i.e., with residues K427 and Y431 in G-m^6^A-CA and with residues F395, K427, D463, and R466 in G-m^6^A-CU) compared to adenine. Apart from the further stability provided by the methyl group in both G-m^6^A-CU and G-m^6^A-CA, the interactions of m^6^A in G-m^6^A-CU are improved compared to those of adenine in G-m^6^A-CU, but not necessarily in G-m^6^A-CA. Importantly, in both cases, these occur in conjunction with improvement of interactions formed by nucleotides 1, 5, and, to a lesser extent, 4 (as well as 6 for G-m^6^A-CA over GACA). Notably, this occurred in tandem with an adjustment of nucleotides 4, 5, and 6 toward the C-terminal domain, as well as the linkers between RRM2-HOA and HOA-RRM3, and an enhanced interdomain interaction between the C-terminal and linker between RRM2-HOA. This highlights the role of other domains beyond RRM3, i.e., the C-terminal, HOA, and linkers, in the interaction of RBM45 with RNA, particularly m^6^A-modified RNA. Thus, RNA, and particularly m^6^A-modified RNA, binding could play a key role in complex conformation and interdomain arrangement. Overall, these factors contribute to the higher affinity of RBM45 for G-m^6^A-CA and G-m^6^A-CU compared to GACA and GACU, respectively.

Our proposed detailed mechanism of m^6^A preferential binding examines in detail the role of residues beyond RRM3, including the HOA domain, C-terminal domain, and linkers. This also supports the key role of RBM45 linkers and the C-terminal domain, which are intrinsically disordered in the absence of RNA, in the binding of RNA comprising GACA/GACU motifs, which conforms with previous findings.^19^ Our simulations depict that they become less flexible in the presence of RNA, and thus, they could possess a key role in binding GACA/GACU-containing RNAs. Importantly, we further suggest how RNAs containing methylation could be associated with even more improved binding compared to unmodified RNAs. This highlights how conformational dynamics and interactions involving linkers, which are intrinsically disordered domains of RBM45, can influence the protein’s capacity for GACA/GACU binding and preferential binding of m^6^A over adenine. Therefore, our study suggests an additional possible important role of such regions beyond their role in homo-oligomerization mediating self-association^76^ and association with other RBPs.^6,10^ Furthermore, our study “dissected” m^6^A binding to different RBM45 domains RRM1-RRM2 and RRM3 individually.

Our findings, along with those of previous studies,^4,7,27^ suggest that unmodified and m^6^A-modified GACA and GACU motifs can be recognized by RRM1–RRM2 and RRM3. What distinguishes RBM45 as a preferential binder for m^6^A-containing motifs could potentially be attributed to its RRM3 domain working synergistically with residues before and after RRM3 due to the packing of RNA in complex with the full-length protein. The higher binding free energy favorability of m^6^A over adenine in the complex with RRM3 in the context of the full-length RBM45 protein could outweigh the slightly lower binding free energy favorability of m^6^A over adenine in the complex with RRM1-RRM2. In this context, we consider that overall, a methylated RNA strand will have significantly lower binding free energy in complex with RRM3 and perhaps slightly higher binding free energy in complex with RRM1-RRM2. Thus, the preferential binding of 462 nM vs 1371 nM, as reported for an RNA strand comprising the G-m^6^A-CU versus GACU motif in complex with full-length RBM45, as reported by Choi et al.,^4^ reflects a favorability of ∼ 0.6 kcal/mol. This favorability could be considered a combination of the higher favorability of G-m^6^A-CU versus GACU for RRM3 (∼1.7 kcal/mol) and the slightly higher favorability of GACU versus G-m^6^A-CU for RRM1 (∼0.2 kcal/mol). It is important to note that G-m^6^A-CU and GACU-containing motifs were predicted to be high-affinity binders for RRM1, RRM2, as well as RRM3 in the context of the full-length protein. Thus, their binding to different RRMs of RBM45 could likely occur and should be considered collectively. Yet, the binding favorability of methylated over unmethylated motifs is suggested to be attributed to their binding to RRM3 in synergism with other domains.

Overall, our study not only complies with previous findings by Chen et al.^7,27^ and Choi et al.^4^ but also provides an understanding of the mechanism underlying previous findings by filling gaps and uncovering m^6^A preferential binding properties by RBM45. This can pave the way for future studies focusing on the role of RRM3 and additional domains involved in the preferential binding mechanism, investigating its significance in biological and disease-related axes. Additionally, given RBM45 implication in neurodegenerative diseases,^4,5,10,11,14,16^ and at the same time the key role of m^6^A in such diseases (reviewed in ref 77), understanding the m^6^A preferential binding properties to RBM45 should be considered a key step for further future studies of the connection between the two in biology and health, or exploiting the delicate recognition of m^6^A by RRMs for the design of systems for precision biomedical applications.^78^

Elucidating m^6^A preferential binding to RBM45 could be considered a key step toward understanding the m^6^A binding properties of RRM-containing proteins in general. Some members of the HNRNP family containing RRM domains act as m^6^A reader proteins; HNRNPC^79^ and HNRNPG^29^ act as m^6^A-switch readers.^28^ As for m^6^A recognition by HNRNPA2B1,^31,32^ we performed a combination of computational and experimental studies on HNRNPA2B1, suggesting binding but not selective recognition of m^6^A.^33^ In this context, considering that RBM45 is a preferential binder of m^6^A, along with its high affinity for GAC-containing motifs that coincide with the motif in which m^6^A is written, RBM45 can be considered an exemplary case to elucidate key principles of m^6^A binding to RRMs, and shed light on the direct and preferential binding of m^6^A by RRMs. Our study presents the first, to our knowledge, mechanism by which an RRM domain, in synergy with other RRM domains, binds directly and preferentially recognizes m^6^A over adenine. Considering RBM45 as an m^6^A reader, attributed to its preferential binding to m^6^A compared to adenine, our study suggests that RBM45 could be classified within Class III of m^6^A readers, according to the classification provided by Zhou et al.^28^ Notably, our simulations present structural-based insights into how an RBP, in general, as well as an RRM-containing protein in particular, of this class can preferentially bind m^6^A over adenine. We consider that our study could provide an impetus for elucidating m^6^A preferential binding by proteins of this class, including RRMs and beyond.

## Supplementary Material











## Notes

RMSD calculations and clustering analysis, average interaction free energies of residue-nucleotide pairs, per-nucleotide interaction free energies, and distances as a function of time during different simulation trajectories (Tables and Figures) (PDF)
RBM45-G6CA (PDB)
RBM45-GACA (PDB)
RBM45-G6CU (PDB)
RBM45-GACU (PDB)
