# Supplementary material for: RBM45 Preferential Binding to m6A: Simulations Suggest Synergy of RRM3 and Other Domains
Source: J Phys Chem B. 2026 May 15;130(21):5310–34. doi: 10.1021/acs.jpcb.6c01856 (PMC13224180; doi:10.1021/acs.jpcb.6c01856)

Supporting Information

RBM45 Preferential Binding to m^6A: Simulations Suggest Synergy of RRM3 and Other Domains

Raeyeon Park[a], Lydia M. Contreras[b], Phanourios Tamamis[a,c]*

[a] Artie McFerrin Department of Chemical Engineering, Texas A&M University, College Station, TX, USA

[b] McKetta Department of Chemical Engineering, The University of Texas at Austin, Austin, TX, USA.

[c] Department of Materials Science & Engineering, Texas A&M University, College Station, TX, USA.

*Corresponding Author: Phanourios Tamamis, tamamis@tamu.edu, 979-862-1610

Table S1A. Root mean square deviation (RMSD) for RRM1-RRM2:RNA complexes. Trajectories were aligned to backbone atoms of the RRM domain and the bound RNA. RMSD values (Å) were calculated relative to the average structure of each trajectory.

		RRM1	RNA bound to RRM1			RRM1	RNA bound to RRM1			RRM1	RNA bound to RRM1
Entry 1	run1	1.62	2.37	Entry 3	run1	1.37	1.81	Entry 5	run1	1.92	2.46
	run2	2.24	2.77		run2	1.57	2.50		run2	1.69	2.38
	run3	1.35	1.80		run3	1.64	2.10		run3	1.37	2.38
	average	1.74	2.31		average	1.52	2.14		average	1.66	2.41
	st. deviation	0.45	0.48		st. deviation	0.14	0.34		st. deviation	0.27	0.05
Entry 2	run1	3.87	5.79	Entry 4	run1	1.43	2.14	Entry 6	run1	1.59	3.22
	run2	1.58	2.43		run2	1.96	2.71		run2	1.78	2.47
	run3	2.01	2.76		run3	1.22	2.05		run3	2.02	3.32
	average	2.49	3.66		average	1.53	2.30		average	1.80	3.00
	st. deviation	1.22	1.85		st. deviation	0.38	0.36		st. deviation	0.22	0.47
		RRM2	RNA bound to RRM2			RRM2	RNA bound to RRM2			RRM2	RNA bound to RRM2
Entry 1	run1	1.14	1.73	Entry 8	run1	1.37	2.01	Entry 10	run1	3.04	4.03
	run2	1.21	2.56		run2	1.40	2.23		run2	1.15	2.65
	run3	1.61	2.64		run3	1.32	2.24		run3	1.70	2.85
	average	1.32	2.31		average	1.36	2.16		average	1.96	3.18
	st. deviation	0.25	0.50		st. deviation	0.04	0.13		st. deviation	0.98	0.74
Entry 7	run1	1.60	2.37	Entry 9	run1	1.33	2.92	Entry 11	run1	1.48	3.21
	run2	1.81	3.53		run2	1.42	2.10		run2	1.00	1.50
	run3	1.73	2.64		run3	1.41	2.90		run3	1.59	2.82
	average	1.72	2.85		average	1.39	2.64		average	1.35	2.51
	st. deviation	0.10	0.61		st. deviation	0.05	0.47		st. deviation	0.31	0.89

Table S1B. Root mean square deviation (RMSD) for RRM3:RNA complexes. Trajectories were aligned to backbone atoms of the RRM domain and the bound RNA. RMSD values (Å) were calculated relative to the average structure of each trajectory.

		RRM3	RNA bound to RRM3			RRM3	RNA bound to RRM3			RRM3	RNA bound to RRM3
Entry 12	run1	0.69	2.61	Entry 14	run1	1.11	4.51	Entry 16	run1	0.74	2.92
	run2	0.61	2.11		run2	0.89	3.43		run2	0.77	3.66
	run3	0.71	2.80		run3	0.96	3.88		run3	1.79	6.48
	run4	0.69	3.01		run4	0.98	3.76		run4	0.65	2.68
	run5	0.71	2.59		run5	1.17	5.04		run5	0.87	3.05
	run6	0.72	2.85		run6	2.28	7.53		run6	0.96	3.68
	average	0.69	2.66		average	1.23	4.69		average	0.96	3.74
	st. deviation	0.04	0.31		st. deviation	0.52	1.50		st. deviation	0.42	1.40
Entry 13	run1	0.72	2.82	Entry 15	run1	1.04	4.02	Entry 17	run1	0.96	4.03
	run2	0.95	4.28		run2	0.73	3.67		run2	0.71	2.44
	run3	0.76	3.08		run3	0.62	1.76		run3	1.00	5.54
	run4	0.72	2.82		run4	0.67	3.05		run4	0.77	2.79
	run5	0.79	3.15		run5	2.36	8.63		run5	1.70	6.33
	run6	0.84	2.92		run6	0.92	4.18		run6	0.70	2.42
	average	0.80	3.18		average	1.06	4.22		average	0.97	3.92
	st. deviation	0.09	0.55		st. deviation	0.66	2.33		st. deviation	0.38	1.68

Table S1C. Root mean square deviation (RMSD) for RRM3:full-length RBM45:RNA complexes. Trajectories were 1) aligned to backbone atoms of the RRM domain and the bound RNA (highlighted in dark gray), and 2) aligned to backbone atoms of the entire protein and the bound RNA (highlighted in lighter gray). RMSD values (Å) were calculated relative to the average structure of each trajectory. N-terminal is defined as residues 1-25, linker 1 refers to regions in between RRM1-RRM2 (107-120), linker 2 refers to regions in between RRM2-HOA (196-247), linker 3 refers to regions between HOA-RRM3(325-391) and C-terminal is residues 465-476.

RMSD with respect to average structure		Alignment of RRM3-RNA		Alignment of the full-length Protein-RNA											
		RRM3	RNA bound to RRM3	All	Protein	RNA bound to RRM3	RRM1	RRM2	HOA	RRM3	N-Ter	linker1	linker2	linker3	C-Ter
Entry 18	run1	0.57	2.10	2.14	2.13	2.50	1.71	1.25	2.58	1.32	2.40	1.12	2.96	2.24	2.61
	run2	0.68	2.48	1.86	1.83	2.80	1.58	0.98	1.52	1.28	1.84	0.93	2.38	1.74	5.32
	run3	0.77	2.83	2.25	2.21	3.24	1.82	1.22	1.93	1.71	2.15	1.06	2.05	2.56	6.90
	run4	0.75	2.34	1.73	1.70	2.58	1.49	1.21	1.50	1.70	2.17	0.99	1.74	1.98	2.63
	run5	0.88	2.76	2.44	2.36	4.32	1.84	1.30	2.01	1.93	2.92	1.76	3.19	2.28	6.01
	run6	0.61	1.56	2.77	2.78	2.25	2.02	1.17	1.76	1.58	8.18	1.49	3.16	2.15	3.89
	average	0.71	2.34	2.20	2.17	2.95	1.74	1.19	1.88	1.59	3.28	1.23	2.58	2.16	4.56
	st. deviation	0.11	0.47	0.38	0.39	0.75	0.19	0.11	0.40	0.25	2.43	0.33	0.61	0.28	1.79
Entry 19	run1	0.73	1.96	1.89	1.88	2.19	1.54	1.04	1.51	1.31	2.24	1.00	2.53	2.19	4.32
	run2	0.69	2.58	1.94	1.91	3.02	1.80	1.19	1.88	1.53	2.13	1.10	1.66	2.12	4.61
	run3	0.68	2.08	2.31	2.28	3.39	1.58	1.19	1.94	1.84	1.84	1.06	4.08	2.18	4.26
	run4	0.56	1.07	1.46	1.47	1.26	1.71	0.98	1.69	1.11	1.77	1.06	1.48	1.40	1.49
	run5	0.52	1.00	1.98	1.98	1.73	1.63	1.26	2.27	1.53	2.24	1.25	2.02	2.48	2.89
	run6	0.53	0.97	1.63	1.64	1.24	1.69	1.07	1.90	1.28	1.66	1.06	1.62	1.97	1.64
	average	0.62	1.61	1.87	1.86	2.14	1.66	1.12	1.86	1.43	1.98	1.09	2.23	2.06	3.20
	st. deviation	0.09	0.68	0.29	0.28	0.90	0.10	0.11	0.26	0.26	0.25	0.08	0.98	0.36	1.40
Entry 20	run1	0.70	1.70	2.93	2.95	1.86	2.10	1.56	2.26	1.71	8.75	1.30	2.17	2.48	3.79
	run2	0.65	2.29	2.68	2.67	2.96	1.71	1.28	1.98	1.61	7.94	1.12	1.88	1.95	5.09
	run3	0.77	3.20	2.96	2.91	4.68	2.25	1.29	2.31	2.22	6.98	1.44	2.55	2.49	6.36
	average	0.56	1.74	2.86	2.84	3.17	2.02	1.38	2.18	1.85	7.89	1.29	2.20	2.31	5.08
	st. deviation	0.24	0.91	0.16	0.15	1.42	0.28	0.16	0.18	0.33	0.89	0.16	0.33	0.31	1.29
Entry 21	run1	0.65	1.59	1.75	1.73	2.12	1.73	1.17	1.41	1.50	1.61	1.09	1.90	1.92	4.03
	run2	0.58	1.91	2.55	2.56	2.32	1.96	1.12	1.81	1.51	7.72	1.31	2.62	1.89	2.47
	run3	0.62	2.27	2.60	2.60	2.80	1.88	1.26	2.67	1.50	5.96	1.38	3.25	2.15	2.90

	average	0.57	1.94	2.30	2.30	2.41	1.86	1.18	1.96	1.50	5.10	1.26	2.59	1.98	3.13
	st. deviation	0.18	0.76	0.48	0.49	0.35	0.12	0.07	0.65	0.01	3.14	0.15	0.68	0.14	0.81

Table S2A. Intermolecular polar and nonpolar average interaction free energies (kcal/mol), hydrogen bonds, salt bridges and coulombic interactions, between 5′-GACGGGACGC-3′, 5′-G-m^6A-CGGGACGC-3′ and RBM45-RRM1 residue pairs. Nucleotide-residue pairs that have average interaction free energy lower or equal to -1 kcal/mol for either polar or nonpolar average interaction energy components are presented. Nucleotide-residue pairs that have average interaction free energies higher than -1 kcal/mol may be shown if the corresponding unmodified or m^6A-modified motif has average interaction free energy below -1 kcal/mol for comparison. Analysis for each nucleotide-residue pair per entry is presented individually, and involves nucleotides 1, 2, 3, 4 and 5, in conjunction with the discussion in the main text. **a.** Selected nucleotides of 10-mer oligomers in complex with RBM45-RRM1 interacting with RBM45-RRM1 residues. **b.** Selected RBM45-RRM1 residues interacting with 10-mer oligomers and their corresponding average polar and nonpolar average interaction free energy values (kcal/mol) with each nucleotide. Coulombic interactions (<-4 kcal/mol average polar interaction free energy) between nucleotide-residue pairs are noted with ($^{\#}$), whereas weak coulombic interactions (-1.5≤ kcal/mol, ≥-4 kcal/mol average polar interaction free energy) are noted with ($^{\#\#}$). **c.** Hydrogen bonds between nucleotide-residue atom pairs and their corresponding % occupancy for atom pairs with average % occupancy $\gtrsim$30 %, except in cases where the combined occupancy of multiple atom pairs within the same residue-nucleotide is $\gtrsim$30%.

**Nucleotide** [a]	**RBM45 residues (polar, nonpolar average interaction free energies)** [b]	**Hydrogen bonds (% occupancy)** [c]
5′-GACGGGACGC-3′ (Entry 1)		
Gua1	Phe29 (-0.6, -2.4)<br>Val31 (-0.1, -2.1)<br>Lys66 (-0.3, -0.2)<br>Ile68 (-0.2, -1.4)<br>Lys100 (-2.0$^{\#\#}$, -1.0)<br>Phe102 (-0.6, -4.4)<br>His112 (-0.5, -0.3)<br>Asp114 (-8.7, -0.3)	Gua1 N2:Asp114 OD1 (34.6%)<br>Gua1 N2:Asp114 OD2 (30.1%)<br>Gua1 N1:Asp114 OD2 (29.3%)<br>Gua1 N1:Asp114 OD1 (22.5%)
Ade2	Arg27 (-1.2, -0.6)<br>Phe29 (-0.5, -7.2)<br>Lys66 (-0.7, -0.2)<br>Ile68 (-0.4, -1.2)<br>Phe70 (-0.1, -2.3)<br>Phe102 (0.5, -1.3)<br>Ile103 (-2.9, -0.8)<br>Ala104 (-3.4, -1.9)<br>Gln105 (-4.6, -3.0)<br>Arg107 (-1.0, -0.3)<br>His112 (-1.3, -1.8)	Ade2 N1:Gln105 N (99.7%)<br>Ade2 N6:Ile103 O (84.0%)
Cyt3	Arg27 (-14.4$^{\#}$, -0.9)<br>Trp55 (-0.2, -1.7)<br>Val57 (-0.1, -1.2)<br>Lys60 (-1.2, -0.2)<br>Lys66 (-0.8, -0.3)<br>Phe70 (0.9, -3.6)<br>Gln105 (-1.3, -1.2)<br>Ser106 (-1.0, -1.2)<br>Arg107 (-6.3$^{\#}$, -5.7)<br>His112 (-0.1, -0.4)	Cyt3 N4:Gln105 O (91.3%)<br>Cyt3 O2:Arg27 NH1 (51.6%)<br>Cyt3 N3:Arg27 NH2 (48.2%)<br>Cyt3 O2:Arg27 NH2 (34.9%)<br>Cyt3 N4:Ser106 O (33.8%)

Gua4	Arg27 (-6.1[#], -0.7)<br>Asp53 (-12.9, 0.1)<br>Trp55 (-0.5, -9.3)<br>Val57 (-0.1, -0.7)<br>Arg58 (-0.1, -0.4)<br>Lys60 (-3.6[##], -0.5)<br>Lys63 (-0.2, 0.0)<br>Arg107 (-13.3[#], -2.9)	Gua4 N2:Asp53 OD2 (35.9%)<br>Gua4 N2:Asp53 OD1 (34.8%)<br><br>Gua4 O6:Arg27 NH1(33.3%)<br>Gua4 N1:Asp53 OD2 (27.3%)<br>Gua4 N1:Asp53 OD1 (20.3%)
Gua5	Asp53 (-0.3, -0.4)<br>Arg58 (-0.1, 0.0)<br>Lys60 (-1.9[##], -0.2)<br>Lys63 (-0.2, 0.0)<br>Arg107 (-9.9[#], -0.3)	
5′-G-m^6A-CGGGACGC-3′ (Entry 2)		
Gua1	Phe29 (-0.1, -0.7)<br>Val31 (0.0, -0.9)<br>Lys66 (-1.5[##], -0.8)<br>Ile68 (0.0, -0.9)<br>Lys100 (-0.3, -0.4)<br>Phe102 (0.0, -0.3)<br>His112 (-1.0, -2.0)<br>Asp114 (-1.9, -0.0)	
m^6A2	Arg27 (-1.9[##], -0.6)<br>Phe29 (-1.1, -6.8)<br>Lys66 (-1.1, -0.5)<br>Ile68 (-0.5, -2.2)<br>Phe70 (-0.6, -1.9)<br>Phe102 (0.4, -1.6)<br>Ile103 (-0.6, -0.9)<br>Ala104 (-2.8, -1.9)<br>Gln105 (-4.3, -2.6)<br>Arg107 (-2.2[##], -0.7)<br>His112 (0.1, -1.6)	m^6A2 N1:Gln105 N (94.3%)
Cyt3	Arg27 (-24.5[#], 0.1)<br>Trp55 (-1.1, -1.7)<br>Val57 (-0.5, -2.2)<br>Lys60 (-2.5[##], -0.6)<br>Lys66 (-1.8[##], -0.5)<br>Phe70 (0.9, -4.0)<br>Gln105 (-0.9, -1.2)<br>Ser106 (-1.5, -1.2)<br>Arg107 (-9.7[#], -5.6)<br>His112 (-1.5, -0.4)	Cyt3 O2:Arg27 NH1 (89.3%)<br>Cyt3 N3:Arg27 NH2 (84.5%)<br>Cyt3 N4:Gln105 O (82.4%)<br>Cyt3 O2:Arg27 NH2 (55.4%)<br>Cyt3 N4:Ser106 O (48.8%)
Gua4	Arg27 (-1.2, -0.4)<br>Asp53 (-17.0, 0.1)<br>Trp55 (-1.2, -9.9)	Gua4 N2:Asp53 OD2 (63.2%)<br>Gua4 N2:Asp53 OD1 (54.6%)<br>Gua4 N1:Asp53 OD2 (41.1%)

	Val57 (-0.5, -1.5)<br>Arg58 (-4.4#, -1.6)<br>Lys60 (-6.6#, -0.9)<br>Lys63 (-2.6##, -0.3)<br>Arg107 (-9.6#, -2.0)	Gua4 N1:Asp53 OD1 (39.6%)
Gua5	Asp53 (-3.4, -0.1)<br>Arg58 (-2.7##, -0.6)<br>Lys60 (-4.1#, -1.5)<br>Lys63 (-9.0#, -0.5)<br>Arg107 (-3.5##, -0.3)	Gua5 N2:Asp53 OD1 (14.5%)<br>Gua5 N2:Asp53 OD2 (14.5%)<br>Gua5 N1:Asp53 OD2 (14.0%)<br>Gua5 N1:Asp53 OD1 (10.7%)

Table S2B. Intermolecular polar and nonpolar average interaction free energies (kcal/mol), hydrogen bonds, salt bridges and coulombic interactions, between 5′-GACAGGACGC-3′, 5′-G-m^{6}A-CAGGACGC-3′, 5′-GACUGGACGC-3′, and 5′-G-m^{6}A-CUGGACGC-3′ and RBM45-RRM1 residue pairs. Nucleotide-residue pairs that have average interaction free energy lower or equal to -1 kcal/mol for either polar or nonpolar average interaction free energy components are presented. Nucleotide-residue pairs that have average interaction free energies higher than -1 kcal/mol may be shown if the corresponding unmodified or m^{6}A-modified motif has average interaction free energy below -1 kcal/mol for comparison. Analysis for each nucleotide-residue pair per entry is presented individually, and involves nucleotides 1, 2, 3, 4 and 5, in conjunction with the discussion in the main text. **a.** Selected nucleotides of 10-mer oligomers in complex with RBM45-RRM1 interacting with RBM45-RRM1 residues. **b.** Selected RBM45-RRM1 residues interacting with 10-mer oligomers and their corresponding average polar and nonpolar average interaction free energy values (kcal/mol) with each nucleotide. Coulombic interactions (<-4 kcal/mol average polar interaction free energy) between nucleotide-residue pairs are noted with ([#]), whereas weak coulombic interactions (-1.5≤ kcal/mol, ≥-4 kcal/mol average polar interaction free energy) are noted with ([##]). **c.** Hydrogen bonds between nucleotide-residue atom pairs and their corresponding % occupancy for atom pairs with average % occupancy ≳30 %, except in cases where the combined occupancy of multiple atom pairs within the same residue-nucleotide is ≳30%. The asterisk (*) symbol in hydrogen bonds is used to denote that any of the atoms in the charged, carboxyl or amide, side-chain group can participate in the particular hydrogen-bond formation, and the highest percentage occupancy is reported in this case. All values correspond to averages calculated based on triplicate simulation runs.

**Nucleotide [a]**	**RBM45 residues (polar, nonpolar average interaction free energies) [b]**	**Hydrogen bonds (% occupancy) [c]**
5′-GACAGGACGC-3′ (Entry 3)		
Gua1	Phe29 (-0.7, -2.2)<br>Val31 (-0.2, -2.0)<br>Lys66 (-0.5, -0.2)<br>Ile68 (-0.3, -1.5)<br>Lys100 (-1.5[##], -0.8)<br>Phe102 (-0.9, -3.6)<br>His112 (-0.5, -0.9)<br>Asp114 (-13.7, 0.1)	Gua1 N2:Asp114 OD2 (44.3%)<br>Gua1 N2:Asp114 OD1 (40.8%)<br>Gua1 N1:Asp114 OD1 (38.4%)<br>Gua1 N1:Asp114 OD2 (35.9%)
Ade2	Arg27 (-0.9, -0.5)<br>Phe29 (-0.5, -7.3)<br>Lys66 (-1.1, -0.2)<br>Ile68 (-0.5, -1.6)<br>Phe70 (-0.6, -2.5)<br>Phe102 (0.6, -1.3)<br>Ile103 (-2.5, -1.0)<br>Ala104 (-3.3, -1.8)<br>Gln105 (-4.5, -2.8)<br>Arg107 (-0.8, -0.3)<br>His112 (-0.2, -1.6)	Ade2 N1:Gln105 N (100.0%)<br>Ade2 N6:Ile103 O (78.2%)
Cyt3	Arg27 (-11.7[#], -0.6)<br>Trp55 (-0.2, -1.8)<br>Val57 (-0.2, -1.5)<br>Lys60 (-2.0[##], -0.4)<br>Lys66 (-0.8, -0.3)<br>Phe70 (0.6, -3.8)<br>Gln105 (-0.6, -1.3)	Cyt3 N4:Gln105 O (89.2%)<br>Cyt3 O2:Arg27 NH1 (51.1%)<br>Cyt3 N3:Arg27 NH2(47.4%)<br>Cyt3 N4:Ser106 O (41.1%)<br>Cyt3 O2:Arg27 NH2 (23.6%)<br>Cyt3 N3:Arg27 NH1 (10.0%)

	Ser106 (-1.6, -1.1)<br>Arg107 (-4.0[##], -5.6)<br>His112 (-2.4, -0.3)	
Ade4	Arg27 (-0.2, -0.7)<br>Trp55 (-0.1, -6.2)<br>Lys60 (-2.8[##], -0.2)<br>Arg107 (-14.3[#], -3.2)	
Gua5	Ser20 (-0.0, -1.1)<br>Arg27 (-2.0[##], -0.4)<br>Asp53 (-6.5, -0.1)<br>Trp55 (-0.1, -1.8)<br>Arg58 (-0.5, -1.1)<br>Lys60 (-1.1, -0.8)<br>Lys63 (-1.2, -0.5)<br>Arg107 (-9.4[#], -1.6)	Gua5 N2:ASP53 OD2 (28.3%)<br>Gua5 N1:Asp53 OD2 (22.1%)<br>Gua5 N2:Asp53 OD1 (20.6%)<br>Gua5 N1:Asp53 OD1 (9.8%)
5′-G-m^{6}A-CAGGACGC-3′ (Entry 4)		
Gua1	Phe29 (-0.2, -1.1)<br>Val31 (-0.1, -1.2)<br>Lys66 (-1.8[##], -0.8)<br>Ile68 (-0.2, -1.0)<br>Lys100 (-1.6[##], -0.7)<br>Phe102 (-0.1, -0.8)<br>His112 (-0.8, -1.8)<br>Asp114 (-2.4, -0.3)	Gua1 N2:Asp114 OD1 (12.8%)<br>Gua1 N2:Asp114 OD2 (10.8%)<br>Gua1 N1:Asp114 OD2 (9.2%)
m^{6}A2	Arg27 (-1.4, -0.5)<br>Phe29 (-0.8, -6.4)<br>Lys66 (-2.1[##], -0.7)<br>Ile68 (-0.4, -2.0)<br>Phe70 (-0.7, -2.2)<br>Phe102 (0.4, -1.5)<br>Ile103 (-0.5, -0.7)<br>Ala104 (-2.7, -2.1)<br>Gln105 (-4.0, -2.7)<br>Arg107 (-1.6[##], -0.6)<br>His112 (-2.3, -2.2)	m^{6}A2 N1:Gln105 N (93.7%)
Cyt3	Arg27 (-20.7[#], 0.2)<br>Trp55 (1.3, -1.9)<br>Val57 (-0.3, -1.7)<br>Lys60 (-1.1, -0.5)<br>Lys66 (-1.9[##], -0.3)<br>Phe70 (0.7, -4.1)<br>Gln105 (-1.6, -1.0)<br>Ser106 (-0.9, -1.1)<br>Arg107 (-3.9[##], -6.5)<br>His112 (-1.8, -0.6)	Cyt3 O2:Arg27 NH1 (87.5%)<br>Cyt3 N4:Gln105 O (85.4%)<br>Cyt3 O2:Arg27 NH2 (71.9%)<br>Cyt3 N3:Arg27 NH2 (64.1%)<br>Cyt3 N3:Arg27 NH1 (32.3%)<br>Cyt3 N4:Ser106 O (31.9%)

Ade4	Arg27 (-1.4, -1.6)<br>Trp55 (0.4, -6.6)<br>Lys60 (-1.8[##], -0.5)<br>Arg107 (-13.7[#], -1.2)	
Gua5	Ser20 (-0.6, -1.3)<br>Arg27 (-0.4, -0.2)<br>Asp53 (-6.9, 0.3)<br>Trp55 (0.0, -0.6)<br>Arg58 (-0.1, 0.0)<br>Lys60 (-0.3, -0.1)<br>Lys63 (-0.2, 0.0)<br>Arg107 (-6.4[#], -0.1)	Gua5 N2:Asp53 OD1 (33.0%)<br>Gua5 N2:Asp53 OD2 (28.3%)<br>Gua5 N1:Asp53 OD1 (20.8%)<br>Gua5 N1:Asp53 OD2 (20.8%)
5′-GACUGGACGC-3′ (Entry 5)		
Gua1	Phe29 (-0.8, -2.2)<br>Val31 (-0.1, -2.1)<br>Lys66 (-0.6, -0.4)<br>Ile68 (-0.3, -1.5)<br>Lys100 (-1.1, -1.0)<br>Phe102 (-0.7, -3.3)<br>Gln105 (0.0, -0.2)<br>Arg107 (0.0, -0.1)<br>Ser109 (0.0, 0.0)<br>Gly110 (0.0, 0.0)<br>Ser111 (-0.4, 0.0)<br>His112 (0.0, -0.4)<br>Asp114 (-8.5, -0.2)	Gua1 N2:Asp114 OD2 (32.5%)<br>Gua1 N1:Asp114 OD1 (32.3%)<br>Gua1 N1:Asp114 OD2 (30.6%)<br>Gua1 N2:Asp114 OD1 (29.9%)
Ade2	Arg27 (-1.2, -0.6)<br>Phe29 (-0.7, -7.1)<br>Lys66 (-1.2, -0.3)<br>Ile68 (-0.4, -1.5)<br>Phe70 (-0.5, -2.4)<br>Phe102 (0.5, -1.3)<br>Ile103 (-2.5, -1.0)<br>Ala104 (-3.4, -1.9)<br>Gln105 (-4.2, -2.6)<br>Arg107 (-1.6[##], -0.4)<br>His112 (-0.2, -1.4)	Ade2 N1:Gln105 N (98.5%)<br>Ade2 N6:Ile103 O (74.7%)
Cyt3	Arg27 (-17.1[#], -0.4)<br>Trp55 (-0.5, -1.4)<br>Val57 (-0.1, -1.4)<br>Lys66 (-0.9, -0.4)<br>Phe70 (0.7, -3.8)<br>Gln105 (-1.6, -1.3)<br>Ser106 (-1.0, -1.2)<br>Arg107 (-3.5[##], -5.2)<br>Ser108 (-0.1, -0.3)	Cyt3 N4:Gln105 O (92.7%)<br>Cyt3 O2:Arg27 NH1 (68.5%)<br>Cyt3 N3:Arg27 NH2 (68.0%)<br>Cyt3 O2:Arg27 NH2 (35.8%)<br>Cyt3 N4:Ser106 O (29.4%)

	Ser109 (0.0, -0.2)<br>Gly110 (0.0, -0.1)	
Ura4	Arg27 (-1.3, -1.3)<br>Trp55 (0.1, -3.5)<br>Arg58 (0.0, -0.2)<br>Lys60 (-1.2, -0.4)<br>Lys63 (-0.7, -0.1)<br>Arg107 (-9.4[#], -4.9)	Ura4 O4:Arg27 NH1 (24.6%)<br>Ura4 O4:Arg27 NH2 (9.7%)
Gua5	Ser20 (0.9, -1.2)<br>Asp53 (-0.7, -0.1)<br>Trp55 (0.0, -0.2)<br>Arg107 (-6.7[#], -2.4)	
5′-G-m^{6}A-CUGGACGC-3′ (Entry 6)		
Gua1	Phe29 (0.0, 0.0)<br>Val31 (0.0, -0.2)<br>Lys66 (-1.6[##], -0.5)<br>Ile68 (0.0, -0.5)<br>Lys100 (0.0, 0.0)<br>Phe102 (0.0, 0.0)<br>Gln105 (-1.0, -0.8)<br>Arg107 (-2.8[##], -0.9)<br>Ser109 (-2.2, -1.0)<br>Gly110 (0.0, -1.4)<br>Ser111 (-1.6, -1.9)<br>His112 (-1.2, -2.6)<br>Asp114 (-2.0, -0.6)	
m^{6}A2	Arg27 (-1.7[##], -0.7)<br>Phe29 (-0.7, -5.4)<br>Lys66 (-3.2[##], -0.3)<br>Ile68 (-0.2, -1.5)<br>Phe70 (-0.3, -2.0)<br>Phe102 (0.3, -1.3)<br>Ile103 (-0.7, -0.8)<br>Ala104 (-2.5, -1.7)<br>Gln105 (-3.5, -2.0)<br>Arg107 (-1.4, -1.1)<br>His112 (-0.9, -1.1)	m^{6}A2 N1:Gln105 N (80.0%)
Cyt3	Arg27 (-17.4[#], -0.3)<br>Trp55 (0.5, -2.0)<br>Val57 (-0.1, -1.0)<br>Lys66 (-1.1, -0.1)<br>Phe70 (0.6, -2.8)<br>Gln105 (-0.2, -1.3)<br>Ser106 (-1.7, -1.1)<br>Arg107 (-3.3[##], -3.1)<br>Ser108 (-0.8, -1.2)	Cyt3 N4:Gln105 O (74.5%)<br>Cyt3 N3:Arg27 NH2 (66.9%)<br>Cyt3 O2:Arg27 NH1 (64.6%)<br>Cyt3 N4:Ser106 O (50.6%)<br>Cyt3 O2:Arg27 NH2 (37.6%)

	Ser109 (-1.6, -1.0)<br>Gly110 (-1.1, -0.3)	
Ura4	Arg27 (-2.1[##], -1.0)<br>Trp55 (-0.3, -4.9)<br>Arg58 (-1.0, -0.8)<br>Lys60 (-0.7, 0.0)<br>Lys63 (-2.7[##], -0.2)<br>Arg107 (-5.0[#], -1.9)	Ura4 O4:Arg27 NH1 (19.3%)<br>Ura4 O4:Arg27 NH2 (12.7%)
Gua5	Ser20 (-0.7, -0.7)<br>Asp53 (-4.1, -0.3)<br>Trp55 (-0.5, -1.4)<br>Arg107 (-2.6[##], -0.4)	Gua5 N2:Asp53 OD2 (15.3%)<br>Gua5 N1:Asp53 OD2 (13.5%)<br>Gua5 N2:Asp53 OD1 (13.0%)

Table S3A. Intermolecular polar and nonpolar average interaction free energies (kcal/mol), hydrogen bonds, salt bridges and coulombic interactions, between 5′-GGACGGGACG-3′, 5′-GG-m^{6}A-CGGGACG-3′ and RBM45-RRM2 residue pairs. Nucleotide-residue pairs that have average interaction free energy lower or equal to -1 kcal/mol either polar or nonpolar average interaction free energy components are presented. Nucleotide-residue pairs that have average interaction free energies higher than -1 kcal/mol may be shown if the corresponding unmodified or m^{6}A-modified motif has average interaction free energy below -1 kcal/mol for comparison. Analysis for each nucleotide-residue pair per entry is presented individually, and involves nucleotides 2, 3 4 and 5, in conjunction with the discussion in the main text. **a.** Selected nucleotides of 10-mer oligomers in complex with RBM45-RRM2 interacting with RBM45-RRM2 residues. **b.** Selected RBM45-RRM2 residues interacting with 10-mer oligomers and their corresponding average polar and nonpolar average interaction free energy values (kcal/mol) with each nucleotide. Coulombic interactions (<-4 kcal/mol average polar interaction free energy) between nucleotide-residue pairs are noted with ([#]), whereas weak coulombic interactions (-1.5≤ kcal/mol, ≥-4 kcal/mol average polar interaction free energy) are noted with ([##]). **c.** Hydrogen bonds between nucleotide-residue atom pairs and their corresponding % occupancy for atom pairs with average % occupancy ≳30 %, except in cases where the combined occupancy of multiple atom pairs within the same residue-nucleotide is ≳30%.

**Nucleotide** [a]	**RBM45 residues (polar, nonpolar average interaction free energies)** [b]	**Hydrogen bonds (% occupancy)** [c]
5′-GGACGGGACG-3′ (Entry 1)		
Gua2	Phe124 (-4.2, -5.2)<br>Val125 (0.2, -1.6)<br>Met126 (-0.7, -5.1)<br>Lys161 (-0.6, -0.2)<br>Leu163 (0.6, -2.9)<br>Phe185 (-1.9, -0.6)<br>Arg186 (-9.6[#], -3.5)<br>Ile188 (-0.7, -3.2)	Gua2 N1:Arg186 O (99.3%)<br>Gua2 N2:Phe124 O (88.3%)<br>Gua2 N2:Arg186 O (70.7%)
Ade3	Arg122 (-0.9, -0.9)<br>Phe124 (-0.1, -6.3)<br>Lys161 (-0.9, -0.3)<br>Leu163 (-0.3, -0.8)<br>Tyr165 (-5.3, -2.0)<br>Ile188 (0.5, -1.7)<br>Leu189 (-1.8, -1.2)<br>Ala190 (-3.1, -1.9)<br>Glu191 (-2.6, -3.6)<br>Lys193 (-1.1, -0.2)	Ade3 N1:Glu191 N (100.0%)<br>Ade3 N3:Tyr165 OH (89.2%)<br>Ade3 N6:Leu189 O (68.9%)
Cyt4	Arg122 (-20.5[#], 0.3)<br>Ile152 (0.3, -2.7)<br>Lys155 (-2.1[##], -0.4)<br>Lys161 (-0.9, -0.5)<br>Tyr165 (1.3, -4.5)<br>Glu191 (-1.6, -1.5)<br>Pro192 (-1.6, -1.3)<br>Lys193 (-7.9[#], -3.9)	Cyt4 O2:Arg122 NH1 (98.2%)<br>Cyt4 N3:Arg122 NH2 (96.7%)<br>Cyt4 N4:Glu191 O (93.5%)<br>Cyt4 N4:Pro192 O (72.2%)<br>Cyt4 O2:Arg122 NH2 (41.6%)
Gua5	First homodimer	
	Ser184 (-2.1, -1.2)<br>Arg186 (-3.8[##], -0.6)	

	Second homodimer	
	Ile152 (-0.2, -1.3)<br>Lys153 (0, -0.9)<br>Lys155 (-8.3#, -1.8)<br>Lys193 (-4.6##, -1.2)	
5′-GG-m^{6}A-CGGGACG-3′ (Entry 7)		
Gua2	Phe124 (-3.3, -4.0)<br>Val125 (0.2, -1.3)<br>Met126 (-0.4, -5.5)<br>Lys161 (-1.5##, -0.5)<br>Leu163 (0.3, -3.1)<br>Phe185 (-1.3, -0.5)<br>Arg186 (-9.3#, -3.4)<br>Ile188 (-0.4, -2.2)	Gua2 N1:Arg186 O (76.5%)<br>Gua2 N2:Phe124 O (60.7%)<br>Gua2 N2:Arg186 O (54.4%)
m^{6}A3	Arg122 (-1.1, -0.7)<br>Phe124 (-0.4, -6.3)<br>Lys161 (-1.9##, -0.6)<br>Leu163 (-0.4, -1.3)<br>Tyr165 (-6.0, -1.2)<br>Ile188 (0.5, -1.7)<br>Leu189 (-0.6, -0.9)<br>Ala190 (-2.9, -1.9)<br>Glu191 (-2.5, -2.5)<br>Lys193 (-0.6, -0.2)	m^{6}A3 N1:Glu191 N (95.3%)<br>m^{6}A3 N3:Tyr165 OH (80.0%)
Cyt4	Arg122 (-20.2#, -0.5)<br>Ile152 (0.2, -2.5)<br>Lys155 (-2.7##, -0.4)<br>Lys161 (-1.4, -0.5)<br>Tyr165 (1.3, -3.9)<br>Glu191 (-1.3, -1.3)<br>Pro192 (-1.7, -1.4)<br>Lys193 (-5.3#, -3.9)	Cyt4 O2:Arg122 NH1 (99.2%)<br>Cyt4 N3:Arg122 NH2 (98.3%)<br>Cyt4 N4:Glu191 O (90.2%)<br>Cyt4 N4:Pro192 O (67.2%)<br>Cyt4 O2:Arg122 NH2 (45.4%)
Gua5	First homodimer	
	Ser184 (-0.7, -0.8)<br>Arg186 (-4.3#, -3.6)	
	Second homodimer	
	Ile152 (-0.6, -1.5)<br>Lys153 (-1.8##, -1.2)<br>Lys155 (-7.8#, -2.2)<br>Lys193 (-4.5#, -0.9)	

Table S3B. Intermolecular polar and nonpolar average interaction free energies (kcal/mol), hydrogen bonds, salt bridges and coulombic interactions, between 5′-GGACAGGACG-3′, 5′-GG-m^{6}A-CAGGACG-3′, 5′-GGACUGGACG-3′, and 5′-GG-m^{6}A-CUGGACG-3′ and RBM45-RRM2 residue pairs. Nucleotide-residue pairs that have average interaction free energy lower or equal to -1 kcal/mol either polar or nonpolar average interaction free energy components are presented. Nucleotide-residue pairs that have average interaction free energies higher than -1 kcal/mol may be shown if the corresponding unmodified or m^{6}A-modified motif has average interaction free energy below -1 kcal/mol for comparison. Analysis for each nucleotide-residue pair per entry is presented individually, and involves nucleotides 2, 3 4 and 5, in conjunction with the discussion in the main text. **a.** Selected nucleotides of 10-mer oligomers in complex with RBM45-RRM2 interacting with RBM45-RRM2 residues. **b.** Selected RBM45-RRM2 residues interacting with 10-mer oligomers and their corresponding average polar and nonpolar average interaction free energy values (kcal/mol) with each nucleotide. Coulombic interactions (<-4 kcal/mol average polar interaction free energy) between nucleotide-residue pairs are noted with (#), whereas weak coulombic interactions (-1.5≤ kcal/mol, ≥-4 kcal/mol average polar interaction free energy) are noted with (##). **c.** Hydrogen bonds between nucleotide-residue atom pairs and their corresponding % occupancy for atom pairs with average % occupancy ≳30 %, except in cases where the combined occupancy of multiple atom pairs within the same residue-nucleotide is ≳30%.

Nucleotide [a]	RBM45 residues (polar, nonpolar average interaction free energies) [b]	Hydrogen bonds (% occupancy) [c]
5′-GGACAGGACG-3′ (Entry 8)		
Gua2	Phe124 (-4.2, -4.9)<br>Val125 (0.1, -1.6)<br>Met126 (-0.5, -4.8)<br>Lys155 (-0.1, 0.0)<br>Lys161 (-0.7, -0.3)<br>Leu163 (0.5, -2.7)<br>Phe185 (-1.6, -0.5)<br>Arg186 (-10.2#, -3.3)<br>Ile188 (-0.7, -3.1)<br>Glu191 (0.1, 0.0)<br>Lys193 (0.0, 0.0)	Gua2 N1:Arg186 O (99.7%)<br>Gua2 N2:Phe124 O (84.4%)<br>Gua2 N2:Arg186 O (69.6%)<br>Gua2 N2:Val125 N (33.4%)
Ade3	Phe124 (-0.1, -6.3)<br>Ile152 (0.2, -0.3)<br>Lys155 (-0.3, 0.0)<br>Lys161 (-0.8, -0.3)<br>Leu163 (-0.3, -0.7)<br>Tyr165 (-4.5, -2.0)<br>Ile188 (0.5, -1.5)<br>Leu189 (-1.8, -1.1)<br>Ala190 (-3.2, -1.8)<br>Glu191 (-2.2, -3.4)<br>Lys193 (-0.9, -0.2)	Ade3 N1:Glu191 N (99.2%)<br>Ade3 N3:Tyr165 OH (77.4%)<br>Ade3 N6:Leu189 O (65.1%)
Cyt4	Arg122 (-14.8#, -0.5)<br>Ile152 (0.2, -2.4)<br>Lys155 (-1.0, -0.2)<br>Lys161 (-0.9, -0.4)<br>Tyr165 (0.9, -4.1)<br>Arg167 (-0.3, 0.0)	Cyt4 N4:Glu191 O (94.0%)<br>Cyt4 O2:Arg122 NH1 (67.9%)<br>Cyt4 N3:Arg122 NH2 (67.7%)<br>Cyt4 N4:Pro192 O (63.4%)<br>Cyt4 O2:Arg122 NH2 (27.9%)

	Glu191 (-1.7, -1.6)<br>Pro192 (-1.3, -1.5)<br>Lys193 (-8.4[#], -4.6)	
Ade5	First homodimer	
	Arg186 (-7.4[#], -0.9)	
	Second homodimer	
	Ile152 (-0.3, -1.9)<br>Lys153 (-1.5[##], -2.9)<br>Asn154 (-0.1, -1.1)<br>Lys155 (-4.7[#], -2.7)<br>Lys161 (-1.3, -0.2)<br>Lys193 (-7.7[#], -0.8)	
5′-GG-m^{6}A-CAGGACG-3′ (Entry 9)		
Gua2	Phe124 (-2.5, -3.5)<br>Val125 (0.1, -0.9)<br>Met126 (-0.3, -3.2)<br>Lys155 (-0.4, -0.3)<br>Lys161 (-2.8[##], -0.6)<br>Leu163 (0.3, -2.0)<br>Phe185 (-0.9, -0.4)<br>Arg186 (-6.6[#], -3.2)<br>Ile188 (-0.3, -1.7)<br>Glu191 (-1.2, -0.1)<br>Lys193 (-1.1, -0.1)	Gua2 N1:Arg186 O (60.6%)<br>Gua2 N2:Arg186 O (47.8%)<br>Gua2 N2:Phe124 O (47.4%)
m^{6}A3	Phe124 (-0.6, -6.0)<br>Ile152 (0.1, -1.1)<br>Lys155 (-1.1, -0.2)<br>Lys161 (-2.8[##], -0.6)<br>Leu163 (-0.3, -1.4)<br>Tyr165 (-4.9, -1.4)<br>Ile188 (0.5, -1.6)<br>Leu189 (-0.5, -0.9)<br>Ala190 (-2.8, -1.7)<br>Glu191 (-2.7, -2.7)<br>Lys193 (-7.3[#], -0.8)	m^{6}A3 N1:Glu191 N (93.5%)<br>m^{6}A3 N3:Tyr165 OH (73.0%)
Cyt4	Arg122 (-11.5[#], -0.3)<br>Ile152 (0.2, -2.9)<br>Lys155 (-3.9[##], -0.5)<br>Lys161 (-1.7[##], -0.5)<br>Tyr165 (0.7, -4.4)<br>Arg167 (-2.2[##], -0.3)<br>Glu191 (-0.9, -1.0)<br>Pro192 (-0.9, -0.9)<br>Lys193 (-5.2[#], -3.8)	Cyt4 O2:Arg122 NH1 (62.6%)<br>Cyt4 N3:Arg122 NH2 (61.9%)<br>Cyt4 N4:Glu191 O (57.9%)<br>Cyt4 N4:Pro192 O (44.9%)<br>Cyt4 O2:Arg122 NH2 (30.6%)

Ade5	First homodimer	
	Arg186 (-4.8[#], -1.8)	
	Second homodimer	
	Ile152 (-0.3, -1.2)<br>Lys153 (-1.3, -1.2)<br>Asn154 (0.0, -0.3)<br>Lys155 (-11.8[#], -1.9)<br>Lys161 (-0.4, 0.0)<br>Lys193 (-5.7[#], -1.1)	
5′-GGACUGGACG-3′ (Entry 10)		
Gua2	Phe124 (-3.6, -4.9)<br>Val125 (0.2, -1.5)<br>Met126 (-0.6, -4.6)<br>Lys161 (-0.7, -0.2)<br>Leu163 (0.6, -2.6)<br>Phe185 (-1.6, -0.5)<br>Arg186 (-10.1[#], -3.1)<br>Ile188 (-0.9, -2.8)	Gua2 N1:Arg186 O (94.8%)<br>Gua2 N2:Phe124 O (78.4%)<br>Gua2 N2:Arg186 O (70.9%)
Ade3	Arg122 (-1.0, -0.9)<br>Phe124 (-0.1, -6.2)<br>Ile152 (0.2, -0.3)<br>Lys161 (-1.0, -0.4)<br>Leu163 (-0.2, -0.9)<br>Tyr165 (-5.6, -2.1)<br>Ile188 (0.5, -1.5)<br>Leu189 (-1.6, -1.1)<br>Ala190 (-3.2, -1.8)<br>Glu191 (-2.3, -3.3)	Ade3 N1:Glu191 N (99.2%)<br>Ade3 N3:Tyr165 OH (96.5%)<br>Ade3 N6:Leu189 O (59.6%)
Cyt4	Arg122 (-21.4[#], 0.3)<br>Ile152 (0.4, -2.8)<br>Lys155 (-1.4, -0.5)<br>Lys161 (-0.9, -0.5)<br>Tyr165 (1.6, -4.7)<br>Glu191 (-1.5, -1.3)<br>Pro192 (-1.6, -1.2)<br>Lys193 (-4.9[#], -3.5)<br>Asn194 (-0.2, -1.7)	Cyt4 O2:Arg122 NH1 (100.0%)<br>Cyt4 N3:Arg122 NH2 (99.0%)<br>Cyt4 N4:Glu191 O (95.3%)<br>Cyt4 N4:Pro192 O (64.7%)<br>Cyt4 O2:Arg122 NH2 (43.9%)
Ura5	First homodimer	
	Arg186 (-0.4, -0.4)	
	Second homodimer	
	Arg122 (-0.9,-1.0)<br>Ile152 (-0.3, -1.3)<br>Lys153 (-0.5, -0.4)	

	Lys155 (-4.4[#], -0.7)<br>Lys193 (-3.8[##], -1.0)<br>Asn194 (-0.4, -0.5)	
5′-GG-m^{6}A-CUGGACG-3′ (Entry 11)		
Gua2	Phe124 (-4.2, -4.8)<br>Val125 (0.3, -1.6)<br>Met126 (-0.6, -5.5)<br>Lys161 (-1.1, -0.3)<br>Leu163 (0.2, -3.0)<br>Phe185 (-1.5, -0.6)<br>Arg186 (-10.1[#], -3.4)<br>Ile188 (-0.6, -2.7)	Gua2 N1:Arg186 O (94.2%)<br>Gua2 N2:Phe124 O (75.0%)<br>Gua2 N2:Arg186 O (68.7%)
m^{6}A3	Arg122 (-1.4, -0.7)<br>Phe124 (-0.4, -6.2)<br>Ile152 (-0.0, -1.2)<br>Lys161 (-2.9[#], -0.9)<br>Leu163 (-0.5, -1.4)<br>Tyr165 (-5.9, -1.5)<br>Ile188 (0.5, -1.6)<br>Leu189 (-0.4, -0.6)<br>Ala190 (-2.8, -2.0)<br>Glu191 (-2.7, -2.5)	m^{6}A3 N3:Tyr165 OH (97.8%)<br>m^{6}A3 N1:Glu191 N (97.3%)
Cyt4	Arg122 (-21.4[#], -0.6)<br>Ile152 (0.3, -3.0)<br>Lys155 (-2.6[##], -0.9)<br>Lys161 (-4.3[#], -0.1)<br>Tyr165 (1.4, -4.6)<br>Glu191 (-1.6, -1.3)<br>Pro192 (-1.6, -1.5)<br>Lys193 (-5.2[#], -4.0)<br>Asn194 (0.3, -1.6)	Cyt4 O2:Arg122 NH1 (99.2%)<br>Cyt4 N3:Arg122 NH2 (98.5%)<br>Cyt4 N4:Glu191 O (91.0%)<br>Cyt4 N4:Pro192 O (68.9%)<br>Cyt4 O2:Arg122 NH2 (30.8%)
Ura5	First homodimer	
	Arg186 (-5.4, -0.9)	
	Second homodimer	
	Arg122 (-1.0, -0.4)<br>Ile152 (-0.1, -1.2)<br>Lys153 (0.0, -0.8)<br>Lys155 (-12.1[#], -2.4)<br>Lys193 (-3.8[##], -1.5)<br>Asn194 (-1.0, -0.9)	Ura5 O4′:Lys155 NZ (30.9%)

Table S4A. Intermolecular polar and nonpolar average interaction free energies (kcal/mol), hydrogen bonds, salt bridges and coulombic interactions, between 5′-GACGGG-3′, 5′-G-m^{6}A-CGGG-3′ and RBM45-RRM3 residue pairs. Nucleotide-residue pairs that have average interaction free energy lower or equal to -1 kcal/mol either polar or nonpolar average interaction free energy components are presented. Nucleotide-residue pairs that have average interaction free energies higher than -1 kcal/mol may be shown if the corresponding unmodified or m^{6}A-modified motif has average interaction free energy below -1 kcal/mol for comparison. Analysis for each nucleotide-residue pair per entry is presented individually, and involves nucleotides 1, 2, 3 4 and 5, in conjunction with the discussion in the main text. **a.** Selected nucleotides of 6-mer oligomers in complex with RBM45-RRM3 interacting with RBM45-RRM3 residues. **b.** Selected RBM45-RRM3 residues interacting with 6-mer oligomers and their corresponding average polar and nonpolar average interaction free energy values (kcal/mol) with each nucleotide. Coulombic interactions (<-4 kcal/mol average polar interaction free energy) between nucleotide-residue pairs are noted with ($^{\#}$), whereas weak coulombic interactions (-1.5≤ kcal/mol, ≥-4 kcal/mol average polar interaction free energy) are noted with ($^{\#\#}$). **c.** Hydrogen bonds between nucleotide-residue atom pairs and their corresponding % occupancy for atom pairs with average % occupancy ≳30 %, except in cases where the combined occupancy of multiple atom pairs within the same residue-nucleotide is ≳30%. The asterisk (*) symbol in hydrogen bonds is used to denote that any of the atoms in the charged, carboxyl or amide, side-chain group can participate in the particular hydrogen-bond formation, and the highest percentage occupancy is reported in this case. All values correspond to averages calculated based on triplicate simulation runs.

**Nucleotide** [a]	**RBM45 residues (polar, nonpolar average interaction free energies)** [b]	**Hydrogen bonds (% occupancy)** [c]
5′-GACGGG-3′ (Entry 12)		
Gua1	Phe395 (0.0, -2.2)<br>Lys427 (-0.7, -0.3)<br>Met460 (-0.0, -1.8)<br>Leu461 (-1.4, -0.3)<br>Asp463 (-1.5, -0.6)<br>Arg466 (-0.3, -1.1)	
Ade2	Phe395 (-0.4, -4.3)<br>Lys427 (-4.2$^{\#}$, -0.6)<br>Tyr431 (-5.1, -1.7)<br>*Leu461* (-0.5, -0.6)<br>Ala462 (-2.2, -1.3)<br>Asp463 (-1.3, -1.8)<br>Arg466 (-6.0$^{\#}$, -0.7)	Ade2 N1:Asp463 N (54.4%)<br>Ade2 N3:Tyr431 OH (53.8%)<br>Ade2 N6:Leu461 O (28.5%)
Cyt3	Arg393 (-4.9$^{\#}$, -0.3)<br>Glu420 (0.7, 0.0)<br>Tyr422 (-5.5, -1.2)<br>Val424 (-0.4, -2.1)<br>Lys427 (-1.6$^{\#\#}$, -0.8)<br>Tyr431 (-0.2, -5.3)<br>Lys433 (-0.9, -0.1)<br>Asp463 (-0.8, -1.2)<br>Ser464 (-1.6, -1.0)<br>Pro465 (-0.6, -2.5)<br>Arg466 (-3.9$^{\#\#}$, -3.9)	Cyt3 O2':Tyr422 OH (64.7%)<br>Cyt3 N4:Asp463 O (59.5%)<br>Cyt3 N4:Ser464 O (49.2%)

Gua4	Arg393 (-2.7##, -0.4)<br>Glu420 (-16.6, -0.1)<br>Tyr422 (-0.9, -8.4)<br>Lys433 (-2.6##, -1.3)<br>Arg466 (-6.8#, -0.9)<br>Glu467 (0.1, -0.7)	Gua4 O6:Lys433 NZ (75.9%)<br>Gua4 N1:Glu420 OE1 (52.5%)<br>Gua4 N1:Glu420 OE2 (48.7%)<br>Gua4 N2:Glu420 OE1 (43.6%)<br>Gua4 N2:Glu420 OE2 (42.8%)
Gua5	Arg393 (-0.4, -0.1)<br>Glu420 (-10.3, -0.9)<br>Lys433 (-3.1##, -0.4)<br>Arg466 (-2.0##, -0.5)<br>Glu467 (0.2, -0.3)	Gua5 O6:Lys433 NZ (63.0%)<br>Gua5 N1:Glu420 OE2 (46.8%)<br>Gua5 N1:Glu420 OE1 (45.6%)<br>Gua5 N2:Glu420 OE1 (30.9%)<br>Gua5 N2:Glu420 OE2 (30.1%)
5′-G-m^{6}A-CGGG-3′ (Entry 13)		
Gua1	Phe395 (0.0, -0.9)<br>Lys427 (-3.3##, -0.5)<br>Met460 (0.0, -0.4)<br>Leu461 (0.0, -0.1)<br>Asp463 (-0.2, -0.4)<br>Arg466 (0.0, -0.6)	
m^{6}A2	Phe395 (-0.3, -3.5)<br>Lys427 (-2.5##, -1.2)<br>Tyr431 (-3.0, -1.0)<br>Leu461 (-0.3, -0.5)<br>Ala462 (-1.5, -1.2)<br>Asp463 (-1.9, -1.1)<br>Arg466 (-8.4#, -0.7)	m^{6}A2 N1:Asp463 N (62.2%)<br>m^{6}A2 N3:Tyr431 OH (58.5%)
Cyt3	Arg393 (-1.5##, -0.6)<br>Glu420 (0.7, 0.0)<br>Tyr422 (-4.6, -1.5)<br>Val424 (-0.3, -2.3)<br>Lys427 (-3.8##, -0.2)<br>Tyr431 (-0.2, -5.4)<br>Lys433 (-1.5##, -0.2)<br>Asp463 (-0.9, -0.8)<br>Ser464 (-1.1, -0.9)<br>Pro465 (-0.6, -1.2)<br>Arg466 (-7.1#, -6.0)	Cyt3 O2':Tyr422 OH (41.5%)<br>Cyt3 N4:Asp463 O (39.5%)<br>Cyt3 N4:Ser464 O (37.6%)<br>Cyt3 O2:Tyr422 OH (32.1%)
Gua4	Arg393 (-1.3, -0.2)<br>Glu420 (-12.6, -0.7)<br>Tyr422 (-0.9, -7.0)<br>Lys433 (-6.8#, -0.8)<br>Arg466 (-16.0#, -0.6)<br>Glu467 (0.9, -0.8)	Gua4 O6:Lys433 NZ (67.8%)<br>Gua4 N1:Glu420 OE2 (52.0%)<br>Gua4 N1:Glu420 OE1 (51.5%)<br>Gua4 N2:Glu420 OE2 (46.5%)<br>Gua4 N2:Glu420 OE1 (45.2%)
Gua5	Arg393 (-2.1##, -0.1)<br>Glu420 (-1.7, -0.5)<br>Lys433 (-3.6##, -0.5)	Gua5 O6:Lys433 NZ (31.4%)<br>Gua5 N1:Glu420 OE2 (22.6%)<br>Gua5 N1:Glu420 OE2 (21.3%)

	Arg466 (-5.6#, -4.1)<br>Glu467 (-0.3, -1.6)	Gua5 N2:Glu420 OE1 (13.8%)<br>Gua5 N2:Glu420 OE2 (12.3%)

Table S4B. Intermolecular polar and nonpolar average interaction free energies (kcal/mol), hydrogen bonds, salt bridges and coulombic interactions, between 5′-GACAGG-3′, 5′-G-m^{6}A-CAGG-3′, 5′-GACUGG-3′, and 5′-G-m^{6}A-CUGG-3′ and RBM45-RRM3 residue pairs. Nucleotide-residue pairs that have average interaction free energy lower or equal to -1 kcal/mol either polar or nonpolar average interaction free energy components are presented. Nucleotide-residue pairs that have average interaction free energies higher than -1 kcal/mol may be shown if the corresponding unmodified or m^{6}A-modified motif has average interaction free energy below -1 kcal/mol for comparison. Analysis for each nucleotide-residue pair per entry is presented individually, and involves nucleotides 1, 2, 3 4 and 5, in conjunction with the discussion in the main text. **a.** Selected nucleotides of 6-mer oligomers in complex with RBM45-RRM3 interacting with RBM45-RRM3 residues. **b.** Selected RBM45-RRM3 residues interacting with 6-mer oligomers and their corresponding average polar and nonpolar average interaction free energy values (kcal/mol) with each nucleotide. Coulombic interactions (<-4 kcal/mol average polar interaction free energy) between nucleotide-residue pairs are noted with (#), whereas weak coulombic interactions (-1.5≤ kcal/mol, ≥-4 kcal/mol average polar interaction free energy) are noted with (##). **c.** Hydrogen bonds between nucleotide-residue atom pairs and their corresponding % occupancy. Nucleotide-residue atom pairs that has % occupancy approximate or greater than 30 % are presented. The asterisk (*) symbol in hydrogen bonds is used to denote that any of the atoms in the charged, carboxyl or amide, side-chain group can participate in the particular hydrogen-bond formation, and the highest percentage occupancy is reported in this case. All values correspond to averages calculated based on triplicate simulation runs.

**Nucleotide** [a]	**RBM45 residues (polar, nonpolar average interaction free energies)** [b]	**Hydrogen bonds (% occupancy)** [c]
5′-GACAGG-3′ (Entry 14)		
Gua1	Lys427 (-1.9##, -0.8)<br>Asp463 (-2.9, -0.3)<br>Arg466 (-6.4#, -1.1)	
Ade2	Arg393 (-1.1, -0.7)<br>Phe395 (-0.4, -4.2)<br>Val424 (-0.4, -1.1)<br>Lys427 (-4.9#, -1.2)<br>Val429 (-0.4, -1.2)<br>Tyr431 (-4.6, -2.7)<br>Met460 (0.3, -0.5)<br>Leu461 (-0.5, -0.5)<br>Ala462 (-1.5, -1.5)<br>Asp463 (-2.3, -2.1)<br>Arg466 (-6.7#, -1.4)	Ade2 N1:Asp463 N (64.4%)<br>Ade2 N3:Tyr431 OH (44.4%)<br>Ade2 N6:Leu461 O (29.8%)
Cyt3	Arg393 (-4.4#, -0.5)<br>Tyr422 (-2.6, -0.7)<br>Val424 (-0.2, -1.6)<br>Lys427 (-2.1##, -0.7)<br>Tyr431 (0.2, -3.8)<br>Lys433 (-1.8##, 0.0)<br>Asp463 (-1.2, -1.0)<br>Ser464 (-2.1, -1.0)<br>Pro465 (-1.5, -1.9)<br>Arg466 (-14.5#, -7.7)	Cyt3 N4:Ser464 O (52.9%)<br>Cyt3 N4:Asp463 O (48.2%)<br>Cyt3 O5':Arg466 NH1 (36.4%)<br>Cyt3 O2':Tyr422 OH (14.4%)<br>Cyt3 O2:Tyr422 OH (10.3%)

Ade4	Arg393 (-0.2, -0.1)<br>Glu420 (-0.1, -0.2)<br>Tyr422 (-0.0, -1.1)<br>Lys433 (-0.2, -0.2)<br>Arg466 (-18.7#, -1.5)<br>Glu467 (-0.3, -1.5)	
Gua5	Glu420 (0.0, 0.0)<br>Lys433 (-0.1, 0.0)<br>Arg466 (-4.8#, -3.8)<br>Glu467 (0.3, -0.6)	
5′-G-m^{6}A-CAGG-3′ (Entry 15)		
Gua1	Lys427 (-1.7##, -0.7)<br>Asp463 (0.2, -0.3)<br>Arg466 (-3.2##, -2.2)	
m^{6}A2	Arg393 (-0.6, -0.2)<br>Phe395 (-0.5, -5.5)<br>Val424 (-0.1, -0.4)<br>Lys427 (-4.2#, -0.9)<br>Val429 (-0.4, -1.0)<br>Tyr431 (-4.2, -1.4)<br>Met460 (0.3, -1.3)<br>Leu461 (-0.4, -0.8)<br>Ala462 (-2.0, -1.6)<br>Asp463 (-1.7, -1.9)<br>Arg466 (-10.1#, -1.0)	m^{6}A2 N1:Asp463 N (53.7%)<br>m^{6}A2 N3:Tyr431 OH (52.6%)
Cyt3	Arg393 (-6.0#, -0.5)<br>Tyr422 (-4.3, -0.8)<br>Val424 (-0.2, -1.6)<br>Lys427 (-2.1##, -0.4)<br>Tyr431 (0.0, -4.1)<br>Lys433 (-0.8, -0.1)<br>Asp463 (-1.2, -1.2)<br>Ser464 (-1.4, -1.1)<br>Pro465 (-1.2, -1.5)<br>Arg466 (-8.8#, -6.7)	Cyt3 N4:Asp463 O (51.1%)<br>Cyt3 N4:Ser464 O (48.5%)<br>Cyt3 O2':Tyr422 OH (26.7%)
Ade4	Arg393 (-0.5, -0.2)<br>Glu420 (-3.0, -0.2)<br>Tyr422 (-0.3, -4.2)<br>Lys433 (0.4, -0.7)<br>Arg466 (-11.6#, -2.9)<br>Glu467 (0.4, -0.7)	Ade4 N6:Glu420 OE1 (14.5%)<br>Ade4 N6:Glu420 OE2 (10.8%)
Gua5	Glu420 (-6.7, 0.0)<br>Lys433 (-2.2##, -0.2)<br>Arg466 (-1.0, -1.2)<br>Glu467 (-0.9, -1.3)	Gua5 N1:Glu420 OE1 (13.2%)<br>Gua5 N1:Glu420 OE2 (12.7%)<br>Gua5 N2:Glu420 OE2 (11.2%)<br>Gua5 N2:Glu420 OE1 (8.8%)

5′-GACUGG-3′ (Entry 16)		
Gua1	Lys427 (-1.8[##], -0.2)<br>Lys458 (-1.1, -0.2)<br>Asp463 (-1.6, -0.2)<br>Arg466 (0.1, -1.4)	
Ade2	Phe395 (-0.4, -6.7)<br>Lys427 (-1.7[##], -0.6)<br>Tyr431 (-2.4, -1.3)<br>Met460 (0.4, -1.5)<br>Leu461 (-2.3, -0.6)<br>Ala462 (-2.7, -1.8)<br>Asp463 (-2.2, -3.2)<br>Arg466 (-9.1[#], -1.1)	Ade2 N1:Asp463 N (79.8%)<br>Ade2 N6:Leu461 O (56.3%)<br>Ade2 N3:Tyr431 OH (41.5%)
Cyt3	Arg393 (-1.8[##], -0.2)<br>Tyr422 (-2.1, -0.6)<br>Val424 (-0.3, -1.4)<br>Lys427 (-5.1[#], -0.9)<br>Tyr431 (-0.2, -2.6)<br>Asp463 (-0.8, -0.8)<br>Ser464 (-1.2, -0.5)<br>Arg466 (-4.7[#], -5.9)	Cyt3 N4:Ser464 O (46.0%)<br>Cyt3 N4:Asp463 O (40.4%)<br>Cyt3 O2':Tyr422 OH (33.6%)
Ura4	Glu420 (-0.2, -0.2)<br>Tyr422 (-0.3, -2.2)<br>Lys427 (-3.2[##], -0.2)<br>Tyr431 (0.0, -0.5)<br>Lys433 (-1.5[##], -0.4)<br>Arg466 (-10.6[#], -0.8)	
Gua5	Arg393 (-1.1, -0.2)<br>Glu420 (-4.5, -0.1)<br>Tyr422 (-0.1, -2.0)<br>Lys433 (0.0, -0.4)<br>Arg466 (-4.8[#], -2.9)<br>Glu467 (-0.3, -1.0)	Gua5 O6:Lys433 NZ (33.6%)<br>Gua5 N1:Glu420 OE1 (23.7%)<br>Gua5 N1:Glu420 OE2 (23.5%)<br>Gua5 N2:Glu420 OE1 (21.7%)<br>Gua5 N2:Glu420 OE2 (19.6%)
5′-G-m^{6}A-CUGG-3′ (Entry 17)		
Gua1	Lys427 (-0.8, -0.4)<br>Lys458 (-0.8, -0.2)<br>Asp463 (-1.1, -0.5)<br>Arg466 (-1.4, -1.5)	
m^{6}A2	Phe395 (-0.4, -3.4)<br>Lys427 (-3.8[##], -0.7)<br>Tyr431 (-3.2, -1.0)<br>Met460 (0.2, -0.9)<br>Leu461 (-0.3, -0.6)<br>Ala462 (-1.5, -1.2)<br>Asp463 (-1.7, -1.6)	m^{6}A2 N1:Asp463 N (38.1%)<br>m^{6}A2 N3:Tyr431 OH (37.8%)

	Arg466 (-5.4#, -1.2)	
Cyt3	Arg393 (-0.3, -0.3)<br>Tyr422 (-2.5, -1.1)<br>Val424 (-0.2, -1.4)<br>Lys427 (-2.3##, -0.4)<br>Tyr431 (0.1, -3.4)<br>Asp463 (-0.2, -0.6)<br>Ser464 (-0.4, -0.5)<br>Arg466 (-3.8##, -4.3)	Cyt3 O2':Tyr422 OH (30.8%)<br>Cyt3 N4:Asp463 O (24.5%)<br>Cyt3 N4:Ser464 O (23.0%)
Ura4	Glu420 (0.5, -0.3)<br>Tyr422 (-0.2, -3.4)<br>Lys427 (-0.4, -0.2)<br>Tyr431 (-0.0, -1.2)<br>Lys433 (-3.0##, -0.4)<br>Arg466 (-9.0#, -2.0)	Ura4 O4:Lys433 NZ (35.0%)
Gua5	Arg393 (-0.3, 0.0)<br>Glu420 (-8.4, -0.2)<br>Tyr422 (-0.3, -1.6)<br>Lys433 (-2.9##, -0.4)<br>Arg466 (-8.0#, -0.8)<br>Glu467 (-1.3, -0.8)	Gua5 O6:Lys433 NZ (48.2%)<br>Gua5 N1:Glu420 OE2 (40.5%)<br>Gua5 N1:Glu420 OE1 (40.0%)<br>Gua5 N2:Glu420 OE1 (30.5%)<br>Gua5 N2:Glu420 OE2 (27.4%)

Table S5. Intermolecular polar and nonpolar average interaction free energies (kcal/mol), hydrogen bonds, salt bridges and coulombic interactions, between 5′-GACAGG-3′, 5′-G-m^{6}A-CAGG-3′, 5′-GACUGG-3′, and 5′-G-m^{6}A-CUGG-3′ and RBM45 residue pairs. Nucleotide-residue pairs that have average interaction free energy lower or equal to -1 kcal/mol either polar or nonpolar average interaction free energy components are presented. Nucleotide-residue pairs that have average interaction free energies higher than -1 kcal/mol may be shown if the corresponding unmodified or m^{6}A-modified motif has average interaction free energy below -1 kcal/mol for comparison. Analysis for each nucleotide-residue pair per entry is presented individually, and involves nucleotides 1, 2, 3 4, 5 and 6, in conjunction with the discussion in the main text. **a.** Selected nucleotides of 6-mer oligomer in complex with RBM45-RRM3 interacting with RBM45 residues. **b.** Selected RBM45 residues interacting with 6-mer oligomer and their corresponding average polar and nonpolar average interaction free energy values (kcal/mol) with each nucleotide. Coulombic interactions (<-4 kcal/mol average polar interaction free energy) between nucleotide-residue pairs are noted with ($^{\#}$), whereas weak coulombic interactions (-1.5≤ kcal/mol, ≥-4 kcal/mol average polar interaction free energy) are noted with ($^{\#\#}$). **c.** Hydrogen bonds between nucleotide-residue atom pairs and their corresponding % occupancy for atom pairs with average % occupancy ≳30 %, except in cases where the combined occupancy of multiple atom pairs within the same residue-nucleotide is ≳30%. The asterisk (*) symbol in hydrogen bonds is used to denote that any of the atoms in the charged, carboxyl or amide, side-chain group can participate in the particular hydrogen-bond formation, and the highest percentage occupancy is reported in this case. All values correspond to averages calculated based on sextuplicate (5′-GACAGG-3′, 5′-G-m^{6}A-CAGG-3′) or triplicate (5′-GACUGG-3′, 5′-G-m^{6}A-CUGG-3′) simulation runs.

**Nucleotide** [a]	**RBM45 residues (polar, nonpolar average interaction free energies)** [b]	**Hydrogen bonds (% occupancy)** [c]
5′-GACAGG-3′ (Entry 18)		
Gua1	Lys427 (-1.4, -0.4)<br>Arg466 (-4.7$^{\#}$, -2.7)<br>Asn470 (-1.3, -0.9)<br>Lys471 (-1.6$^{\#\#}$, -0.6)<br>Arg472 (-1.4, -2.7)<br>Gln473 (0.1, -0.1)<br>Arg474 (-0.8, -0.2)	
Ade2	Arg393 (-5.8$^{\#}$, -0.6)<br>Phe395 (-0.3, -5.0)<br>Val424 (-0.1, -0.4)<br>Lys427 (-2.6$^{\#\#}$, -0.5)<br>Val429 (-0.4, -0.8)<br>Tyr431 (-1.1, -1.5)<br>Met460 (0.4, -1.0)<br>Leu461 (-1.3, -0.6)<br>Ala462 (-2.0, -1.4)<br>Asp463 (-0.2, -3.0)<br>Ser464 (-1.0, -0.9)<br>Pro465 (-1.0, -1.3)<br>Arg466 (-13.9$^{\#}$, -4.8)<br>Lys471 (-3.1$^{\#\#}$, -0.3)<br>Arg472 (-4.6$^{\#}$, -0.4)<br>Arg474 (-2.1$^{\#\#}$, -0.3)	Ade2 N1:Asp463 N (47.8%)<br>Ade2 N6:Leu461 O (35.2%)<br>Ade2 N3:Tyr431 OH (27.4%)

Cyt3	Arg393 (-8.3[#], -0.2)<br>Tyr422 (-2.5, -1.1)<br>Val424 (-0.7, -2.1)<br>Lys427 (-3.4[##], -0.7)<br>Tyr431 (-0.9, -2.1)<br>Pro465 (-1.5, -2.1)<br>Arg466 (-12.0[#], -2.5)<br>Lys471 (-3.4[##], -1.2)<br>Arg472 (-4.2[#], -0.7)<br>Arg474 (-2.9[##], -0.7)	Cyt3 O2':Tyr422 OH (21.3%)<br>Cyt3 O2:Tyr422 OH (10.1%)
Ade4	Arg241 (-1.5[##], -0.6)<br>Tyr422 (0.1, -2.1)<br>Val424 (-0.6, -1.6)<br>Ser425 (0.1, -1.8)<br>Lys427 (-5.9[#], -0.1)<br>Arg466 (-1.7[##], -0.6)<br>Lys471 (-3.9[##], -0.6)<br>Arg472 (-1.7[##], -0.7)<br>Arg474 (-13.3[#], -0.9)	
Gua5	Ser206 (-1.1, -0.7)<br>Arg209 (-2.7[##], -4.8)<br>Gln210 (-2.7, -0.6)<br>Arg241 (-1.9[##], -0.9)<br>Gly242 (-0.5, -0.4)<br>Ser425 (0.0, -0.2)<br>Lys471 (-2.6[##], -0.8)<br>Arg472 (-0.7, -1.1)<br>Gln473 (0.6, -1.9)<br>Arg474 (-10.0[#], -5.1)<br>Thr475 (-5.1, -1.9)<br>Tyr476 (-0.7, -2.3)	Gua5 O4':Thr475 N (40.0%)<br>Gua5 N2:Thr475 OG1 (35.5%)<br>Gua5 N3:Thr475 OG1 (34.4%)<br>Gua5 N3:Thr475 N (31.2%)<br>Gua5 N1:Gln210 OE1 (24.0%)<br>Gua5 N2:Gln210 OE1 (16.8%)
Gua6	Glu198 (-5.9, -0.9)<br>Gln202 (-2.5, -3.0)<br>Arg209 (-4.0[##], -1.6)<br>Arg241 (-2.3[##], -1.9)<br>Gly242 (-2.3, -1.1)<br>Gln243 (-0.6, -2.0)<br>Ala245 (-0.2, -0.2)<br>Ile246 (0.0, -0.1)<br>Asp323 (-0.5, -0.1)<br>Thr329 (0.2, -0.4)<br>Asp330 (0.2, -1.1)<br>Arg333 (-5.3[#], -2.5)<br>Ser425 (-1.1, -2.3)<br>Gly426 (0.0, -1.4)<br>Arg472 (-1.6[##], -0.5)	Gua6 O2':Gln202 OE1 (22.9%)<br>Gua6 N2:Glu198 OE2 (14.7%)<br>Gua6 N2:Glu198 OE1 (14.3%)<br>Gua6 N1:Glu198 OE2 (13.6%)<br>Gua6 N1:Glu198 OE1 (10.4%)

5′-G-m^{6}A-CAGG-3′ (Entry 19)		
Gua1	Lys427 (-3.4$^{\#\#}$, -1.0)<br>Arg466 (-6.2$^{\#}$, -1.9)<br>Asn470 (-1.6, -1.5)<br>Lys471 (-1.0, -2.0)<br>Arg472 (-8.2$^{\#}$, -5.6)<br>Gln473 (-1.5, -1.6)<br>Arg474 (-3.3$^{\#\#}$, -1.1)	
m^{6}A2	Arg393 (-1.5$^{\#\#}$, -0.2)<br>Phe395 (-0.5, -4.4)<br>Val424 (-0.2, -0.9)<br>Lys427 (-6.4$^{\#}$, -0.9)<br>Val429 (-0.3, -1.1)<br>Tyr431 (-2.6, -1.4)<br>Met460 (0.2, -1.0)<br>Leu461 (-0.3, -0.6)<br>Ala462 (-1.0, -1.2)<br>Asp463 (-0.2, -1.3)<br>Ser464 (-0.6, -0.7)<br>Pro465 (-0.4, -0.6)<br>Arg466 (-5.5$^{\#}$, -2.4)<br>Lys471 (-1.0, -0.2)<br>Arg472 (-4.1$^{\#}$, -0.7)<br>Arg474 (-0.7, -0.6)	m^{6}A2 N1:Asp463 N (68.3%)<br>m^{6}A2 N3:Tyr431 OH (33.4%)
Cyt3	Arg393 (-3.5$^{\#\#}$, -0.2)<br>Tyr422 (-1.1, -2.2)<br>Val424 (-0.8, -2.1)<br>Lys427 (-1.9$^{\#\#}$, -0.4)<br>Tyr431 (0.5, -1.8)<br>Pro465 (-0.6, -1.5)<br>Arg466 (-7.9$^{\#}$, -2.4)<br>Lys471 (-5.7$^{\#\#}$, -0.6)<br>Arg472 (-10.5$^{\#}$, -2.6)<br>Arg474 (-4.2$^{\#}$, -0.7)	Cyt3 O2':Tyr422 OH (23.2%)
Ade4	Arg241 (-0.3, -0.1)<br>Tyr422 (0.1, -1.7)<br>Val424 (-0.9, -1.5)<br>Ser425 (-1.8, -1.8)<br>Lys427 (-1.2, -0.4)<br>Arg466 (-0.7, -0.1)<br>Lys471 (-4.0$^{\#\#}$, -0.2)<br>Arg472 (-9.8$^{\#}$, -1.0)<br>Arg474 (-21.0$^{\#}$, -2.3)	
Gua5	Ser206 (-0.9, -0.7)<br>Arg209 (-1.2, -4.4)<br>Gln210 (-1.4, -1.0)	Gua5 O4':Thr475 N (87.2%)<br>Gua5 N3:Thr475 OG1 (64.0%)<br>Gua5 N3:Thr475 N (51.6%)

	Arg241 (-1.5[##], -0.9)<br>Gly242 (-1.4, -0.7)<br>Ser425 (-3.9, -0.8)<br>Lys471 (-0.2, -0.1)<br>Arg472 (-2.1[##], -0.5)<br>Gln473 (0.2, -3.1)<br>Arg474 (-15.5[#], -9.5)<br>Thr475 (-8.3, -3.6)<br>Tyr476 (0.2, -2.8)	Gua5 N7:Arg474 NH1 (45.5%)<br>Gua5 N1:Gln210 OE1 (41.5%)<br>Gua5 N2:Thr475 OG1 (40.7%)<br>Gua5 N2:Gln210 OE1 (30.9%)
Gua6	Glu198 (-12.4, -0.7)<br>Gln202 (-4.4, -3.5)<br>Arg209 (-2.3[##], -0.4)<br>Arg241 (-0.2, -0.4)<br>Gly242 (-1.4, -1.1)<br>Gln243 (-4.0, -4.2)<br>Ala245 (-1.2, -0.3)<br>Ile246 (-1.0, -0.4)<br>Asp323 (-2.1, -0.2)<br>Thr329 (-1.0, -1.1)<br>Asp330 (0.8, -1.0)<br>Arg333 (-3.4[##], -4.0)<br>Ser425 (-2.2, -2.1)<br>Gly426 (-0.2, -1.5)<br>Arg472 (-0.1, 0.0)	Gua6 O2':Gln202 OE1 (78.9%)<br>Gua6 N1:Glu198 OE1 (54.6%)<br>Gua6 O2P:Gln243 N (48.4%)<br>Gua6 N2:Glu198 OE1 (48.2%)<br>Gua6 N2:Glu198 OE2 (47.6%)<br>Gua6 N1:Glu198 OE2 (36.6%)<br>Gua6 O3':Ser425 OG (31.8%)<br>Gua6 O1P:Gln243 N (30.9%)<br>Gua6 O2':Ser425 OG (11.8%)
5′-GACUGG-3′ (Entry 20)		
Gua1	Asp203 (0.1, 0.0)<br>Phe395 (-0.2, -2.2)<br>Lys427 (-1.1, -0.6)<br>Asn428 (0.0, -0.2)<br>Asp463 (-1.7, -0.5)<br>Arg466 (-1.1, -1.5)<br>Asn470 (-1.8, -0.8)<br>Arg472 (-1.1, -2.5)	Gua1 O6:Asp463 N (28.6%)<br>Gua1 N1:Asn470 O (16.3%)<br>Gua1 N2:Asn470 O (12.8%)
Ade2	Arg393 (-1.0, -0.4)<br>Phe395 (-0.1, -5.2)<br>Val424 (-0.7, -1.5)<br>Lys427 (-5.6[#], -1.2)<br>Val429 (-0.6, -1.3)<br>Tyr431 (-0.9, -1.6)<br>Met460 (0.3, -0.7)<br>Ala462 (-1.6, -1.5)<br>Asp463 (-1.0, -2.6)<br>Pro465 (-0.3, -1.6)<br>Arg466 (-3.8[##], -1.1)<br>Asn470 (-0.4, -1.0)<br>Lys471 (-1.0, -0.3)	Ade2 N1:Asp463 N (53.1%)<br>Ade2 N6:Asp463 N (10.5%)

	Arg472 (-5.9[#], -1.1)<br>Arg474 (-1.3, -0.3)	
Cyt3	Tyr422 (-0.8, -1.4)<br>Val424 (-0.5, -1.8)<br>Lys427 (-1.1, -0.5)<br>Tyr431 (-1.0, -1.2)<br>Pro465 (-0.1, -1.3)<br>Arg466 (-5.8[#], -1.5)<br>Lys471 (-4.0[##], -2.5)<br>Arg472 (-8.8[#], -2.7)<br>Arg474 (-4.7[#], -1.8)	Cyt3 N4:Arg472 O (27.1%)
Ura4	Tyr422 (-0.6, -1.2)<br>Val424 (-0.6, -1.0)<br>Ser425 (-0.8, -1.4)<br>Lys427 (-1.6[##], -0.3)<br>Lys471 (-1.2, -0.7)<br>Arg472 (-5.4[#], -1.7)<br>Arg474 (-14.4[#], -2.3)	
Gua5	Ser206 (-2.0, -1.0)<br>Arg209 (-4.5[#], -2.9)<br>Gln210 (-1.6, -0.7)<br>Arg241 (-1.1, -0.4)<br>Tyr422 (-1.1, -0.2)<br>Ser425 (-2.2, -0.6)<br>Arg472 (-0.2, -0.4)<br>Gln473 (-0.6, -1.6)<br>Arg474 (-13.2[#], -5.4)<br>Thr475 (-3.0, -1.6)<br>Tyr476 (0.7, -1.8)	Gua5 N7:Arg474 NH1 (36.9%)<br>Gua5 O4':Thr475 N (32.8%)<br>Gua5 N3:Thr475 N (31.8%)<br>Gua5 N2:Thr475 OG1 (29.3%)<br>Gua5 N1:Gln210 OE1 (23.3%)<br>Gua5 O6:Ser206 OG (23.0%)<br>Gua5 N7:Ser206 OG (13.8%)<br>Gua5 N2:Gln210 OE1 (11.3%)
Gua6	Glu198 (-7.1, -0.9)<br>Gln202 (-3.8, -3.8)<br>Tyr205 (-0.3, -2.2)<br>Arg209 (-5.8[#], -0.7)<br>Arg241 (-5.0[#], -0.5)<br>Gly242 (-1.1, -0.6)<br>Gln243 (-3.0, -2.9)<br>Asp330 (0.3, -1.1)<br>Arg333 (-3.3[##], -2.6)<br>Lys334 (-0.5, -0.5)<br>Ser425 (-1.1, -1.7)<br>Arg474 (0.0, -0.8)<br>Tyr476 (-0.1, -1.4)	Gua6 O2':Gln202 OE1 (39.8%)<br>Gua6 N2:Glu198 OE2 (29.6%)<br>Gua6 N1:Glu198 OE2 (28.3%)<br>Gua6 N2:Glu198 OE1 (27.9%)<br>Gua6 N3:Gln202 NE2 (21.6%)
5′-G-m^{6}A-CUGG-3′ (Entry 21)		
Gua1	Asp203 (-3.6, -0.1)<br>Phe395 (0.0, -0.4)<br>Lys427 (-5.7[#], -1.6)	Gua1 N2:Asp203 OD1 (12.5%)<br>Gua1 N1:Asp203 OD1 (11.8%)<br>Gua1 N2:Asp203 OD2 (11.3%)

	Asn428 (-1.4, -0.5)<br>Asp463 (-1.6, -0.4)<br>Arg466 (-4.2[#], -1.7)<br>Asn470 (-1.2, -0.7)<br>Arg472 (-1.4, -2.5)	Gua1 N1:Asp203 OD2 (10.3%)
m^{6}A2	Arg393 (-0.3, -0.4)<br>Phe395 (-0.9, -6.6)<br>Val424 (-0.4, -1.8)<br>Lys427 (-8.1[#], -1.6)<br>Val429 (-0.8, -1.9)<br>Tyr431 (-0.8, -1.3)<br>Met460 (0.3, -1.3)<br>Ala462 (-2.1, -1.7)<br>Asp463 (-2.5, -1.8)<br>Pro465 (-0.5, -0.7)<br>Arg466 (-3.2[##], -2.4)<br>Asn470 (0.0, -0.2)<br>Lys471 (-1.9[##], -0.4)<br>Arg472 (-5.6[#], -1.2)<br>Arg474 (-1.4, -0.2)	m^{6}A2 N1:Asp463 N (88.2%)
Cyt3	Tyr422 (-2.6, -1.9)<br>Val424 (-1.1, -2.5)<br>Lys427 (-1.1, -0.5)<br>Tyr431 (-1.7, -1.8)<br>Pro465 (-0.2, -1.1)<br>Arg466 (-5.6[#], -0.9)<br>Lys471 (-5.3[#], -2.2)<br>Arg472 (-7.9[#], -1.7)<br>Arg474 (-6.0[#], -1.8)	Cyt3 O4':Tyr431 OH (32.1%)<br>Cyt3 O2':Tyr422 OH (30.9%)
Ura4	Tyr422 (-0.9, -1.4)<br>Val424 (-1.0, -1.1)<br>Ser425 (-2.7, -1.3)<br>Lys427 (-0.4, -0.2)<br>Lys471 (-2.4[##], -0.7)<br>Arg472 (-4.3[#], -1.2)<br>Arg474 (-19.5[#], -2.2)	
Gua5	Ser206 (-1.9, -2.2)<br>Arg209 (-5.4[#], -6.1)<br>Gln210 (-2.7, -2.0)<br>Arg241 (-3.5[##], -0.6)<br>Tyr422 (-0.1, 0.0)<br>Ser425 (0.1, -0.2)<br>Arg472 (-2.3[##], -1.2)<br>Gln473 (-0.4, -2.8)<br>Arg474 (-20.9[#], -10.5)<br>Thr475 (-9.1, -3.9)<br>Tyr476 (-2.0, -2.5)	Gua5 O4':Thr475 N (80.0%)<br>Gua5 N3:Thr475 N (63.1%)<br>Gua5 N2:Thr475 OG1 (62.4%)<br>Gua5 N3:Thr475 OG1 (62.2%)<br>Gua5 O1P:Tyr476 OH (43.9%)<br>Gua5 N7:Arg474 NH1 (36.4%)<br>Gua5 O5':Arg474 NH2 (32.8%)<br>Gua5 O5':Arg474 NE (31.6%)<br>Gua5 N1:Gln210 OE1 (29.6%)<br>Gua5 O6:Gln210 NE2 (19.3%)<br>Gua5 N2:Gln210 OE1 (17.6%)

Gua6	Glu198 (-9.6, -0.9)<br>Gln202 (-4.4, -3.8)<br>Tyr205 (-0.5, -1.3)<br>Arg209 (-2.5[##], -0.9)<br>Arg241 (-1.8[##], -0.9)<br>Gly242 (-1.6, -1.2)<br>Gln243 (-3.6, -2.6)<br>Asp330 (-1.5, -0.3)<br>Arg333 (-0.7, -5.1)<br>Lys334 (-0.5, -1.2)<br>Ser425 (-1.5, -1.7)<br>Arg474 (-0.1, -1.0)<br>Tyr476 (-0.2, -0.4)	Gua6 O2':Gln202 OE1 (65.4%)<br>Gua6 N2:Glu198 OE2 (36.9%)<br>Gua6 N2:Glu198 OE1 (35.4%)<br>Gua6 N1:Glu198 OE1 (30.8%)<br>Gua6 O3':Ser425 OG (30.6%)<br>Gua6 O2P:Gln243 N (29.9%)<br>Gua6 N1:Glu198 OE2 (28.3%)<br>Gua6 O1P:Gln243 N (22.8%)

Figure S1. Residue-nucleotide pairwise ΔG interaction free energy (kcal/mol) plot for nucleotide (x-axis) and protein residue (y-axis) pairs calculated for 5′-GACGGGACGC-3′ in complex with RRM1 of RBM45. The plots represent favorable (< 0 kcal/mol) average polar and non-polar ΔG interaction free energies. The ΔG interaction free energy (kcal/mol)-color correspondence is shown on the palette on the right of each plot.

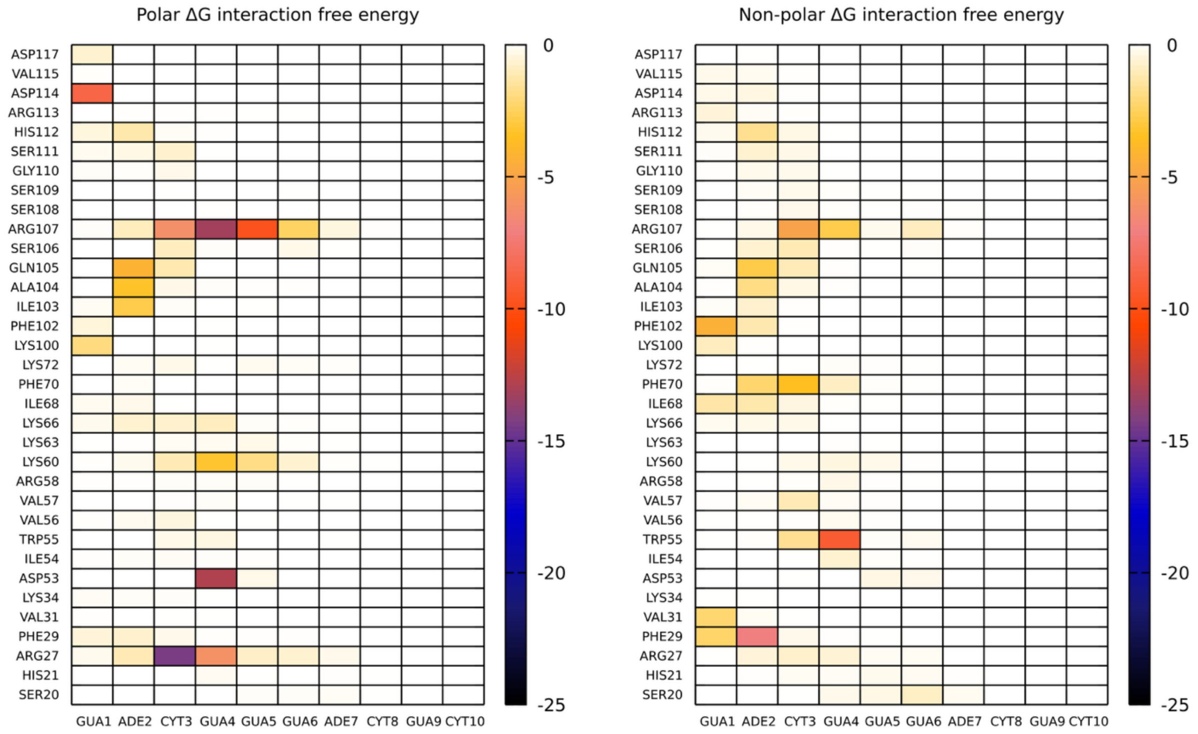

Figure S2. Residue-nucleotide pairwise ΔG interaction free energy (kcal/mol) plot for nucleotide (x-axis) and protein residue (y-axis) pairs calculated for 5′-GGACGGGACG-3′ in complex with RRM2 of RBM45. The plots represent favorable (< 0 kcal/mol) average polar and non-polar ΔG interaction free energies. The ΔG interaction free energy (kcal/mol)-color correspondence is shown on the palette on the right of each plot.

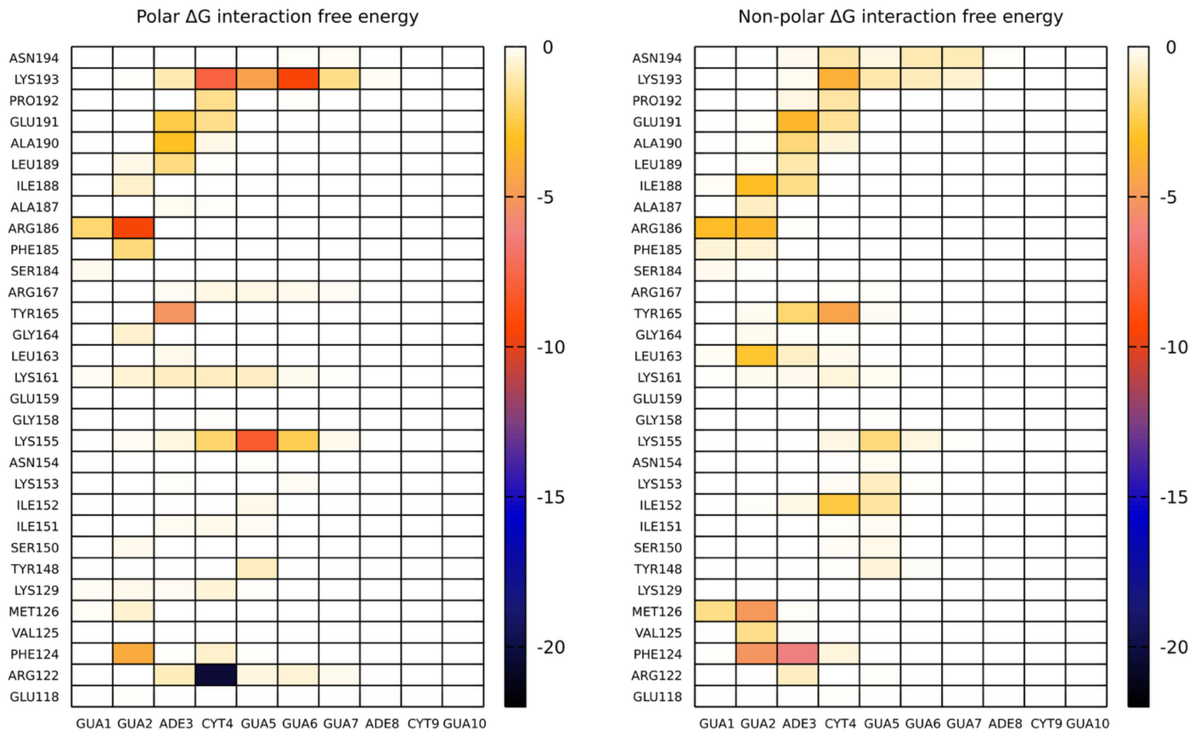

Figure S3. Residue-nucleotide pairwise ΔG interaction free energy (kcal/mol) plot for nucleotide (x-axis) and protein residue (y-axis) pairs calculated for 5′-GACGGG-3′ in complex with RRM3 of RBM45. The plots represent favorable (< 0 kcal/mol) average polar and non-polar ΔG interaction free energies. The ΔG interaction free energy (kcal/mol)-color correspondence is shown on the palette on the right of each plot.

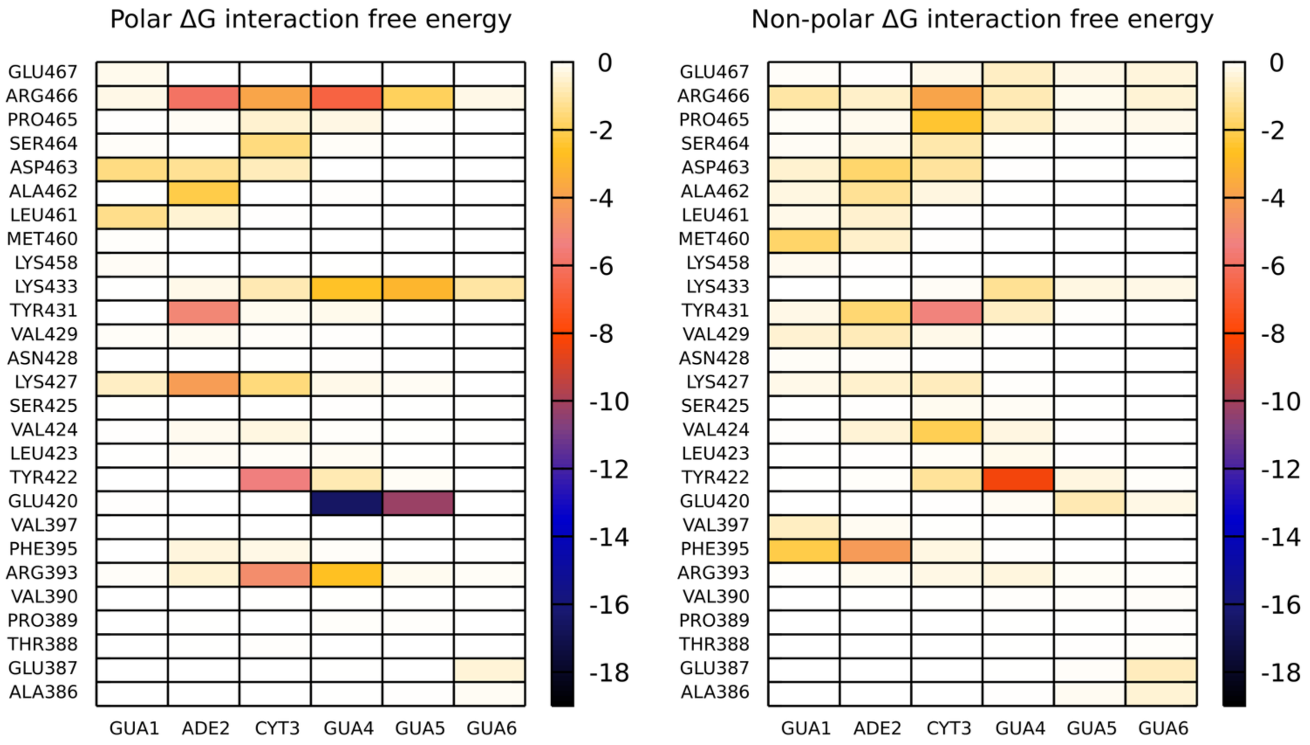

Figure S4. A. Residue-nucleotide pairwise ΔG interaction free energy (kcal/mol) plot for nucleotide (x-axis) and protein residue (y-axis) pairs calculated for 5′-G-m^{6}A-CGGGACGC-3′ in complex with RRM1 of RBM45. The plots represent favorable (< 0 kcal/mol) average polar and non-polar ΔG interaction free energies. **B.** Residue-nucleotide pairwise ΔΔG interaction free energy (kcal/mol) plot for nucleotide (x-axis) and protein residue (y-axis) pairs for 5′-G-m^{6}A-CGGGACGC-3′ compared to 5′-GACGGGACGC-3′ in complex with RRM1 of RBM45. The plots represent ΔΔG interaction free energy of both favorable (< 0 kcal/mol) and unfavorable (>0 kcal/mol) average polar and non-polar ΔG interaction free energies for 5′-G-m^{6}A-CGGGACGC-3′ – 5′-GACGGGACGC-3′. The ΔG and ΔΔG interaction free energy (kcal/mol)-color correspondence is shown on the palette on the right of each plot.

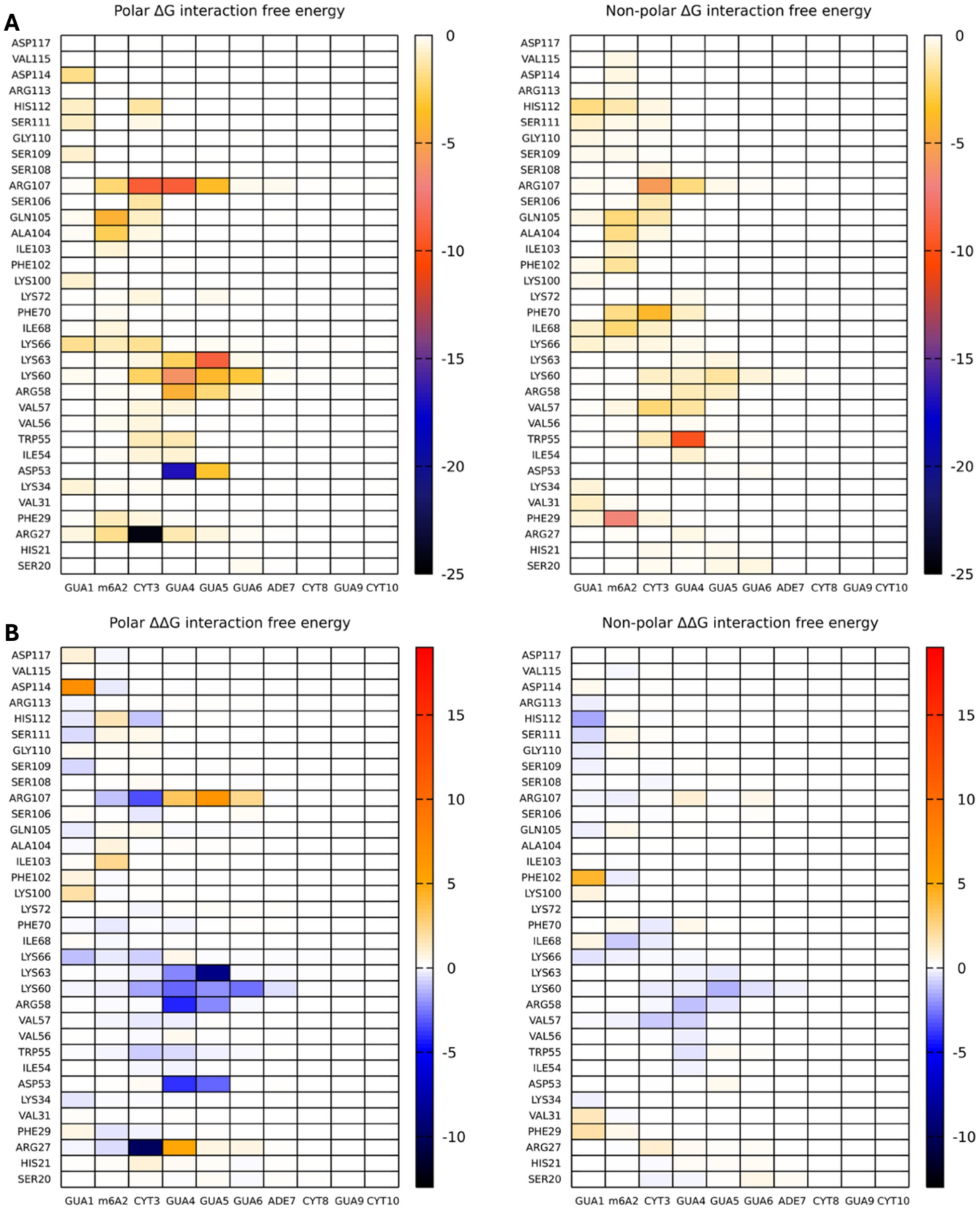

Figure S5. A. Residue-nucleotide pairwise ΔG interaction free energy (kcal/mol) plot for nucleotide (x-axis) and protein residue (y-axis) pairs calculated for 5′-GG-m[6]A-CGGGACG-3′ in complex with RRM2 of RBM45. The plots represent favorable (< 0 kcal/mol) average polar and non-polar ΔG interaction free energies. **B.** Residue-nucleotide pairwise ΔΔG interaction free energy (kcal/mol) plot for nucleotide (x-axis) and protein residue (y-axis) pairs for 5′-GG-m[6]A-CGGGACG-3′ compared to 5′-GGACGGGACG-3′ in complex with RRM2 of RBM45. The plots represent ΔΔG interaction free energy of both favorable (< 0 kcal/mol) and unfavorable (>0 kcal/mol) average polar and non-polar ΔG interaction free energies for 5′-GG-m[6]A-CGGGACG-3′ – 5′-GGACGGGACG-3′. The ΔG and ΔΔG interaction free energy (kcal/mol)-color correspondence is shown on the palette on the right of each plot.

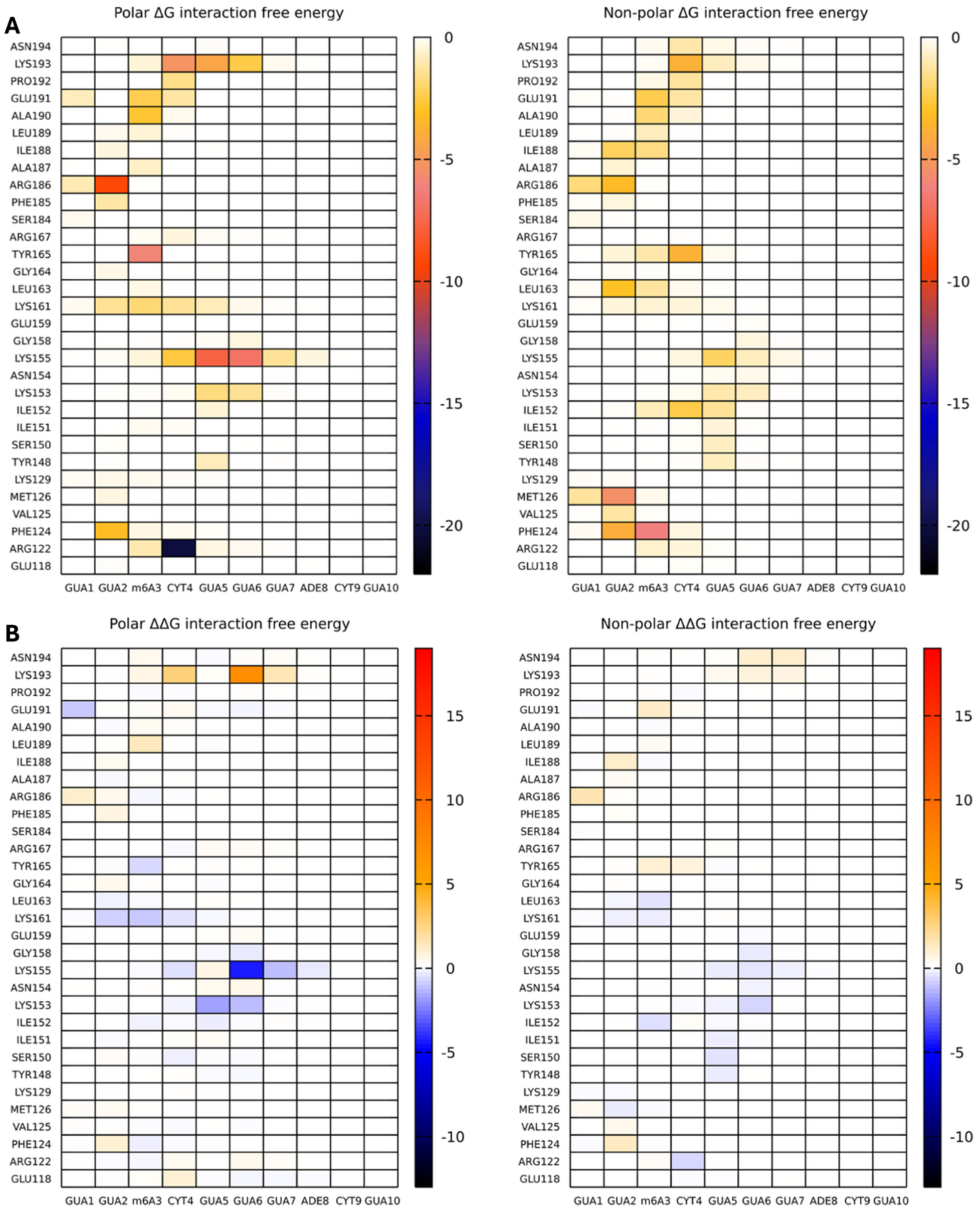

Figure S6. A. Residue-nucleotide pairwise ΔG interaction free energy (kcal/mol) plot for nucleotide (x-axis) and protein residue (y-axis) pairs calculated for 5′-G-m^{6}A-CGGG-3′ in complex with RRM3 of RBM45. The plots represent favorable (< 0 kcal/mol) average polar and non-polar ΔG interaction free energies. **B.** Residue-nucleotide pairwise ΔΔG interaction free energy (kcal/mol) plot for nucleotide (x-axis) and protein residue (y-axis) pairs for 5′-G-m^{6}A-CGGG-3′ compared to 5′-GACGGG-3′ in complex with RRM3 of RBM45. The plots represent ΔΔG interaction free energy of both favorable (< 0 kcal/mol) and unfavorable (>0 kcal/mol) average polar and non-polar ΔG interaction free energies for 5′-G-m^{6}A-CGGG-3′ – 5′-GACGGG-3′. The ΔG and ΔΔG interaction free energy (kcal/mol)-color correspondence is shown on the palette on the right of each plot.

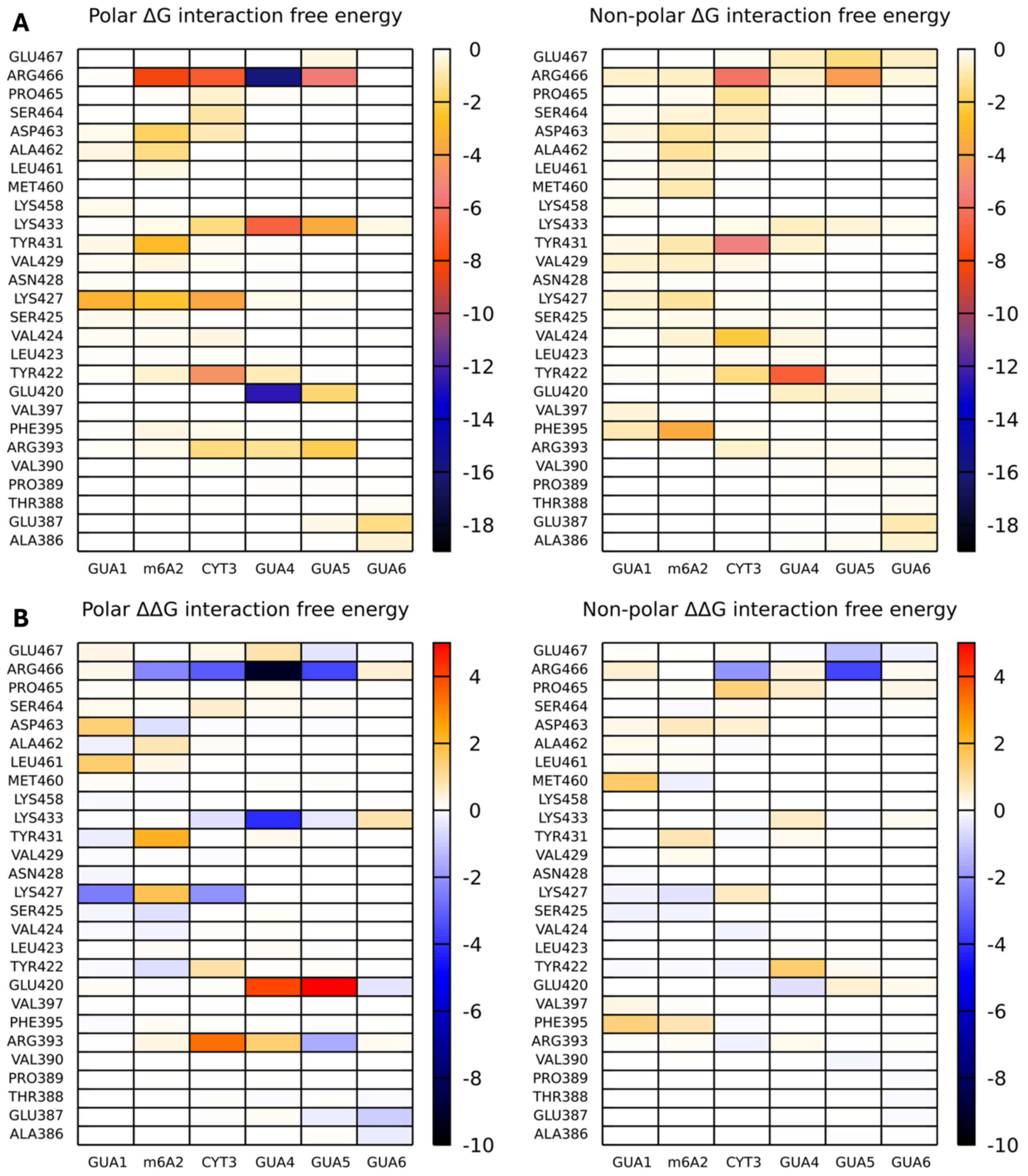

Figure S7. Residue-nucleotide pairwise ΔG interaction free energy (kcal/mol) plot for nucleotide (x-axis) and protein residue (y-axis) pairs calculated for 5′-GACAGGACGC-3′ in complex with RRM1 of RBM45. The plots represent favorable (< 0 kcal/mol) average polar and non-polar ΔG interaction free energies. The ΔG interaction free energy (kcal/mol)-color correspondence is shown on the palette on the right of each plot.

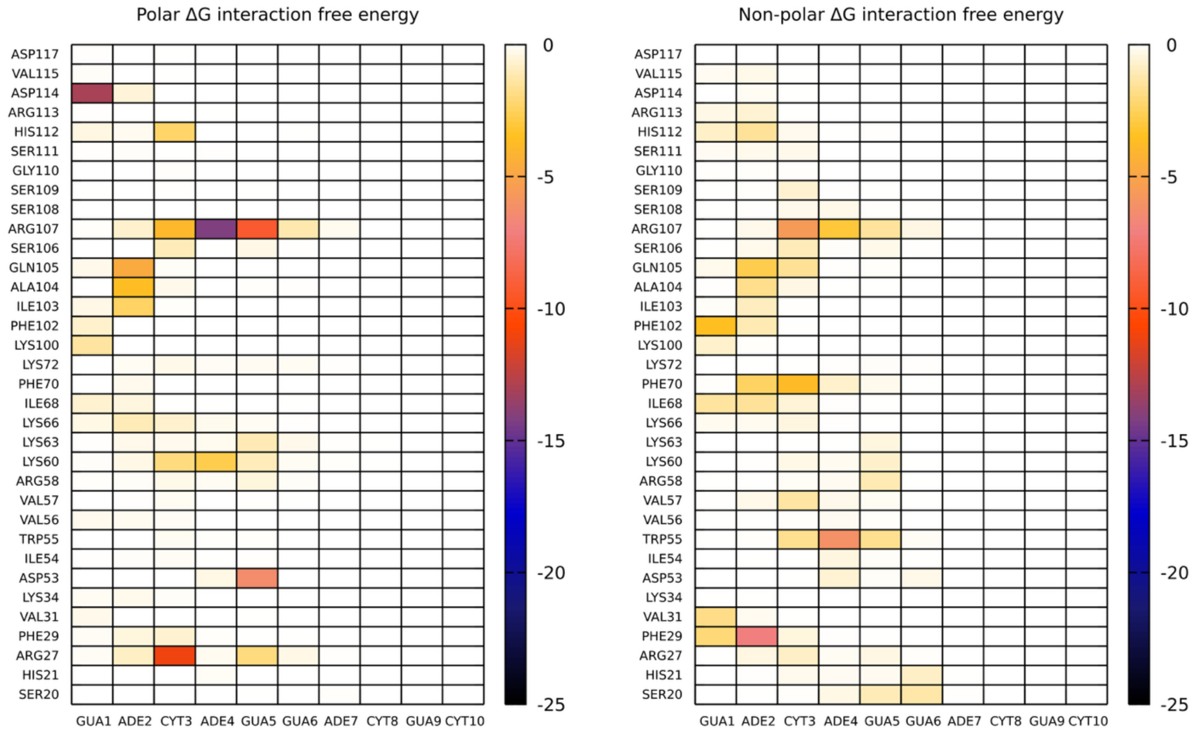

Figure S8A. Residue-nucleotide pairwise ΔG interaction free energy (kcal/mol) plot for nucleotide (x-axis) and protein residue (y-axis) pairs calculated for 5′-G-m^{6}A-CAGGACGC-3′ in complex with RRM1 of RBM45. The plots represent favorable (< 0 kcal/mol) average polar and non-polar ΔG interaction free energies. **B.** Residue-nucleotide pairwise ΔΔG interaction free energy (kcal/mol) plot for nucleotide (x-axis) and protein residue (y-axis) pairs for 5′-G-m^{6}A-CAGGACGC-3′ compared to 5′-GACAGGACGC-3′ in complex with RRM1 of RBM45. The plots represent ΔΔG interaction free energy of both favorable (< 0 kcal/mol) and unfavorable (>0 kcal/mol) average polar and non-polar ΔG interaction free energies for 5′-G-m^{6}A-CAGGACGC-3′ – 5′-GACAGGACGC-3′. The ΔG and ΔΔG interaction free energy (kcal/mol)-color correspondence is shown on the palette on the right of each plot.

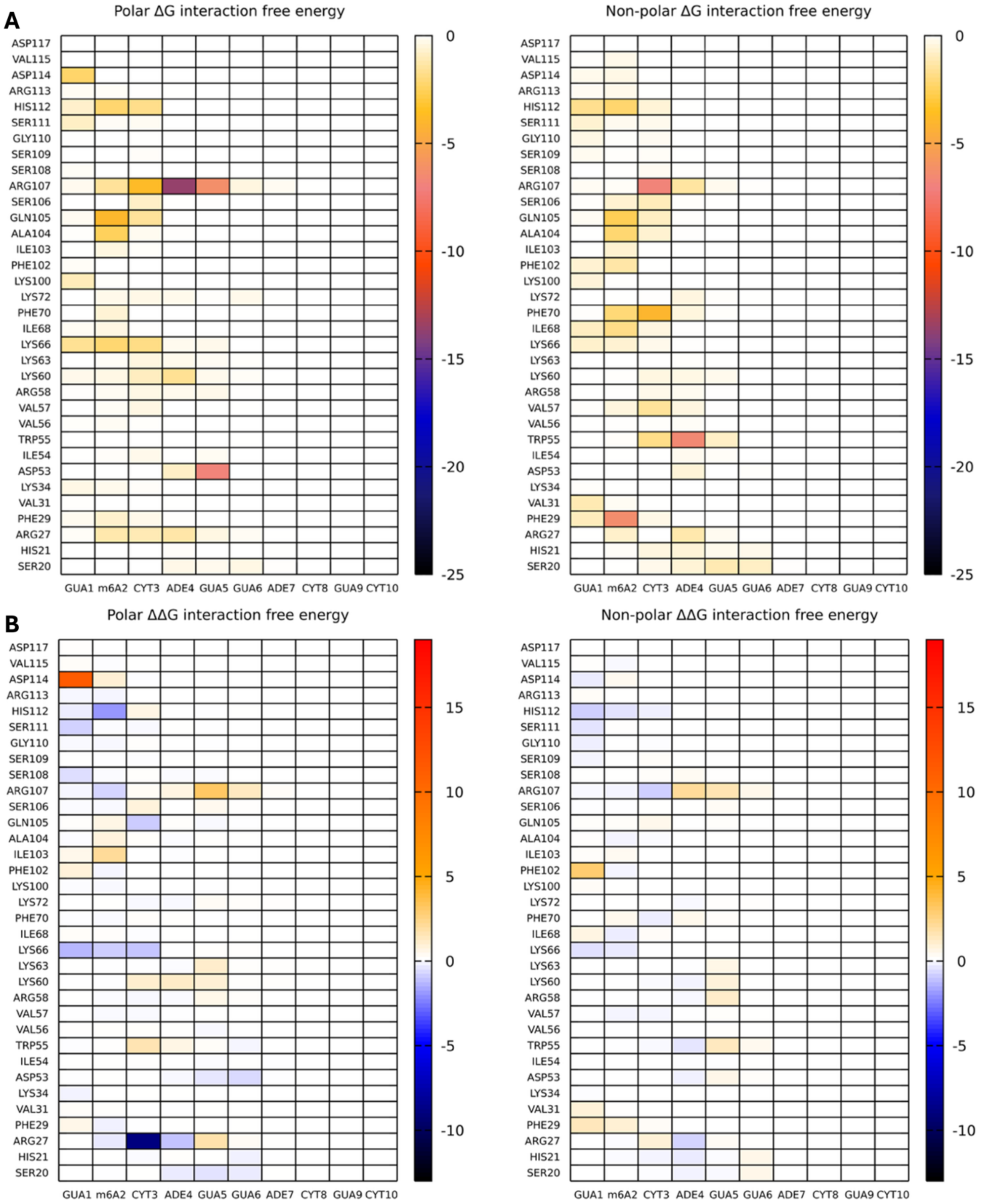

Figure S9. Residue-nucleotide pairwise ΔG interaction free energy (kcal/mol) plot for nucleotide (x-axis) and protein residue (y-axis) pairs calculated for 5′-GACUGGACGC-3′ in complex with RRM1 of RBM45. The plots represent favorable (< 0 kcal/mol) average polar and non-polar ΔG interaction free energies. The ΔG interaction free energy (kcal/mol)-color correspondence is shown on the palette on the right of each plot.

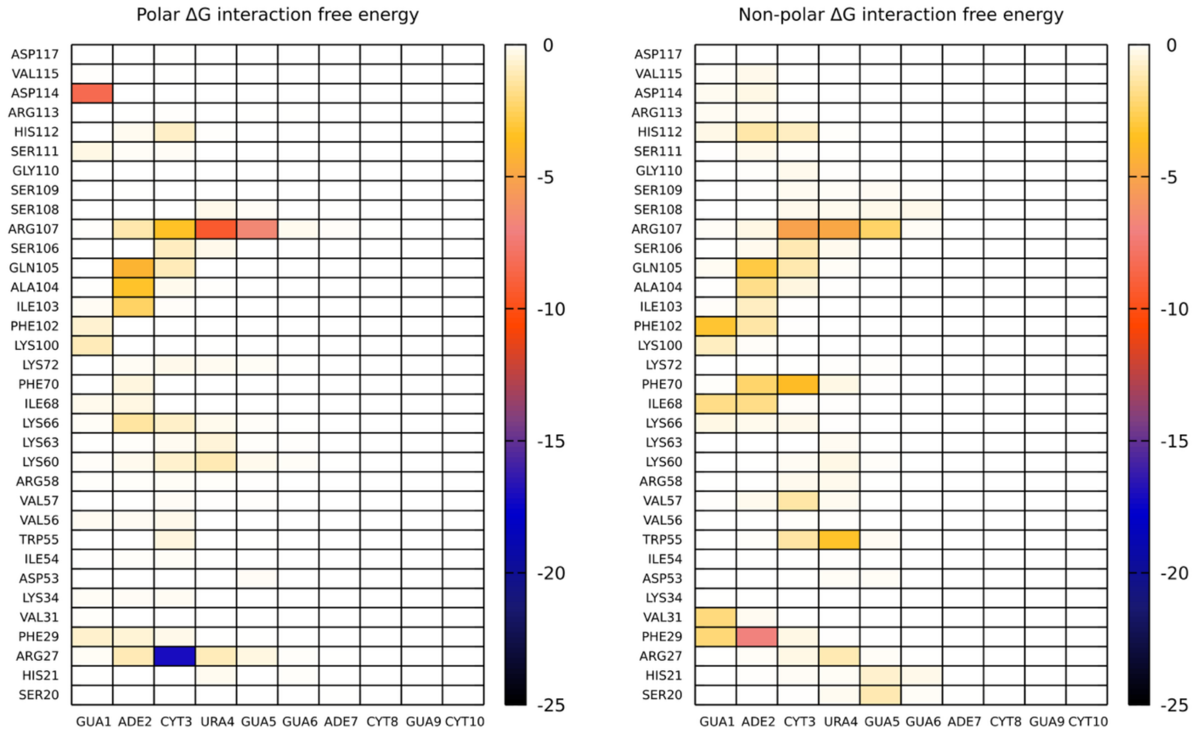

Figure S10. ΔG interaction free energy (kcal/mol) (y-axis) per nucleotide plot calculated for four RNA sequences (x-axis) in complex with RRM1 of RBM45. Purple bars represent polar ΔG interaction free energy (kcal/mol) per nucleotide, and green bars represent non-polar ΔG interaction free energy (kcal/mol) per nucleotide. **A.** 5′-GACAGGACGC-3′ (Entry 3) in complex with RRM1 of RBM45. **B.** 5′-GACUGGACGC-3′ (Entry 5) in complex with RRM1 of RBM45. **C.** 5′-G-m^{6}A-CAGGACGC-3′ (Entry 4) in complex with RRM1 of RBM45. **D.** 5′-G-m^{6}A-CUGGACGC-3′ (Entry 6) in complex with RRM1 of RBM45.

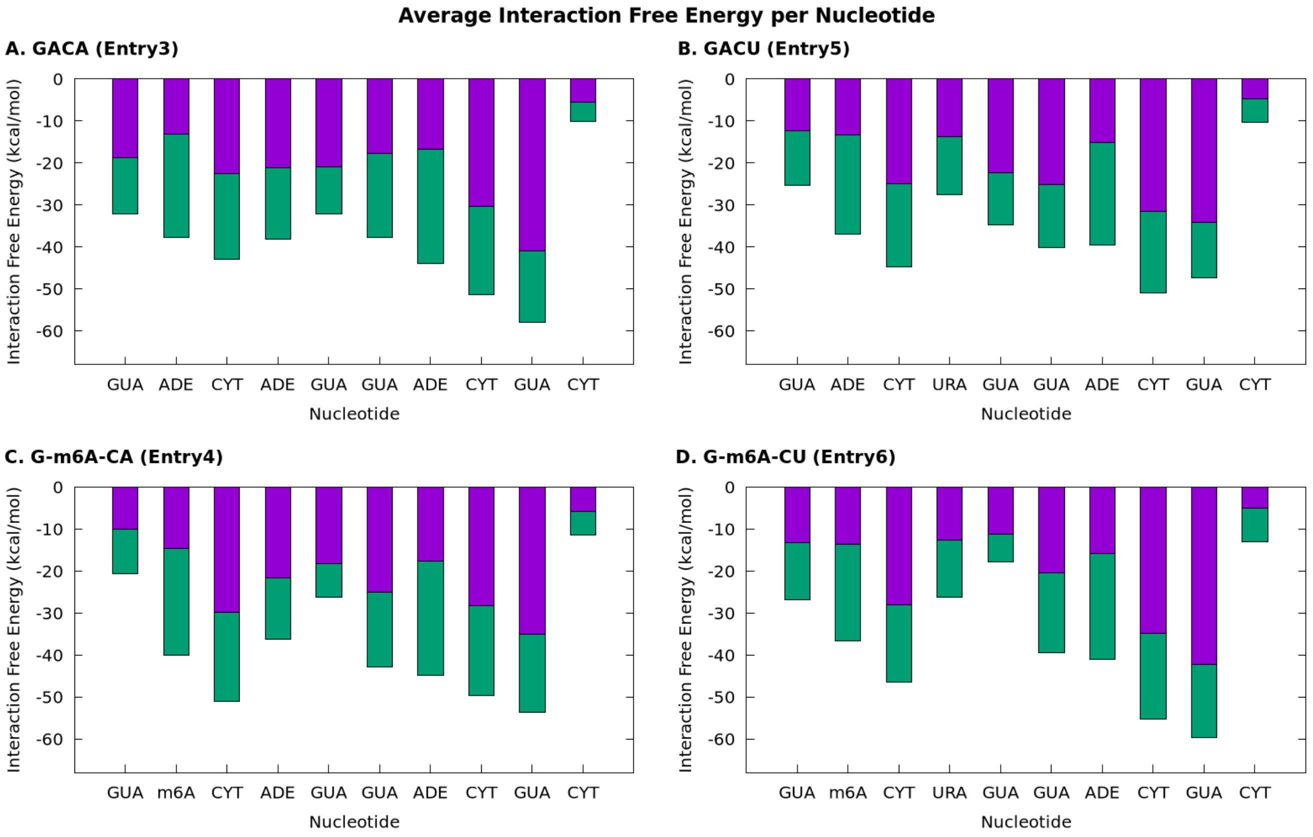

Figure S11A. Residue-nucleotide pairwise ΔG interaction free energy (kcal/mol) plot for nucleotide (x-axis) and protein residue (y-axis) pairs calculated for 5′-G-m[6]A-CUGGACGC-3′ in complex with RRM1 of RBM45. The plots represent favorable (< 0 kcal/mol) average polar and non-polar ΔG interaction free energies. **B.** Residue-nucleotide pairwise ΔΔG interaction free energy (kcal/mol) plot for nucleotide (x-axis) and protein residue (y-axis) pairs for 5′-G-m[6]A-CUGGACGC-3′ compared to 5′-GACUGGACGC-3′ in complex with RRM1 of RBM45. The plots represent ΔΔG interaction free energy of both favorable (< 0 kcal/mol) and unfavorable (>0 kcal/mol) average polar and non-polar ΔG interaction free energies for 5′-G-m[6]A-CUGGACGC-3′ – 5′-GACUGGACGC-3′. The ΔG and ΔΔG interaction free energy (kcal/mol)-color correspondence is shown on the palette on the right of each plot.

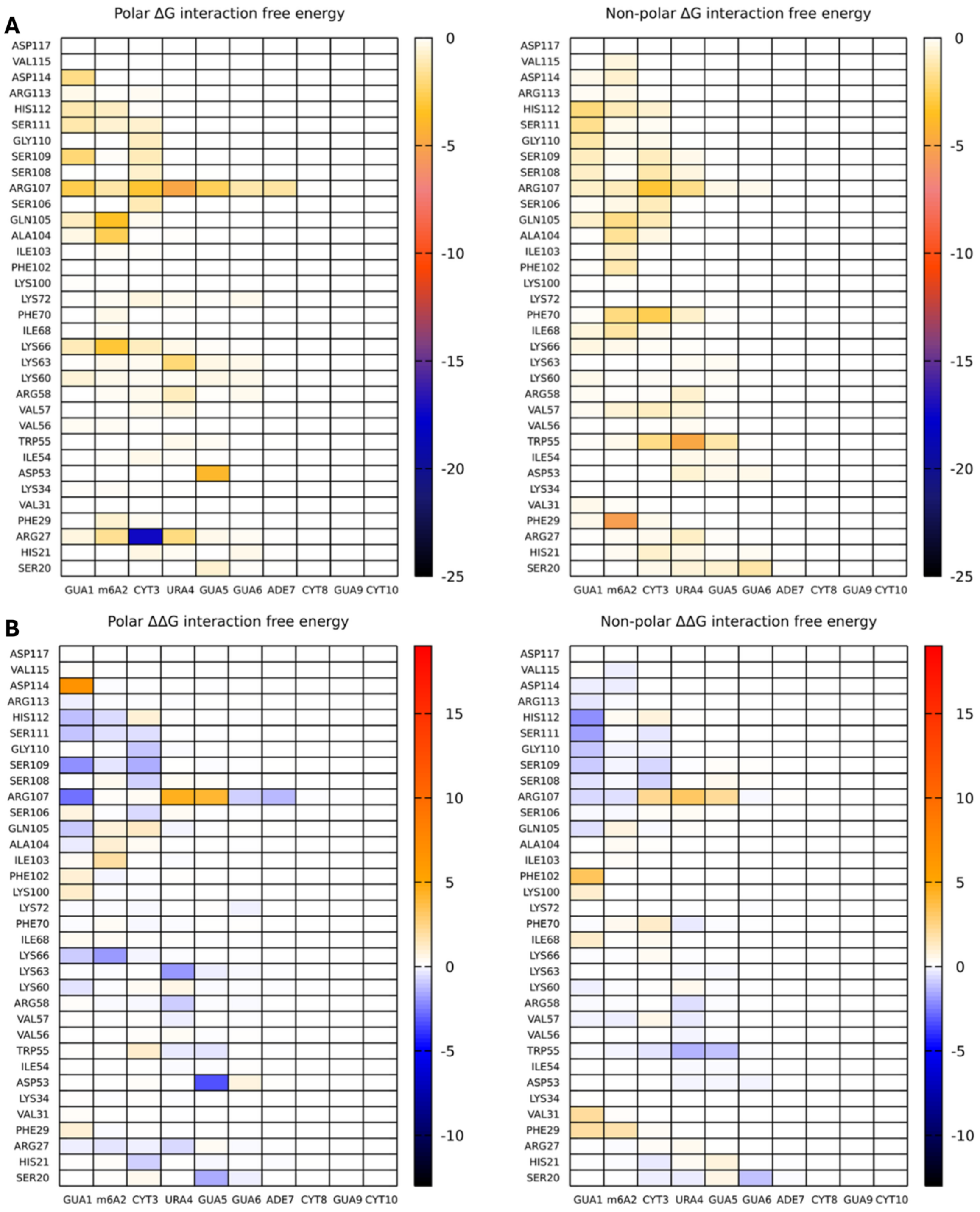

Figure S12. Residue-nucleotide pairwise ΔG interaction free energy (kcal/mol) plot for nucleotide (x-axis) and protein residue (y-axis) pairs calculated for 5′-GGACAGGACG-3′ in complex with RRM2 of RBM45. The plots represent favorable (< 0 kcal/mol) average polar and non-polar ΔG interaction free energies. The ΔG interaction free energy (kcal/mol)-color correspondence is shown on the palette on the right of each plot.

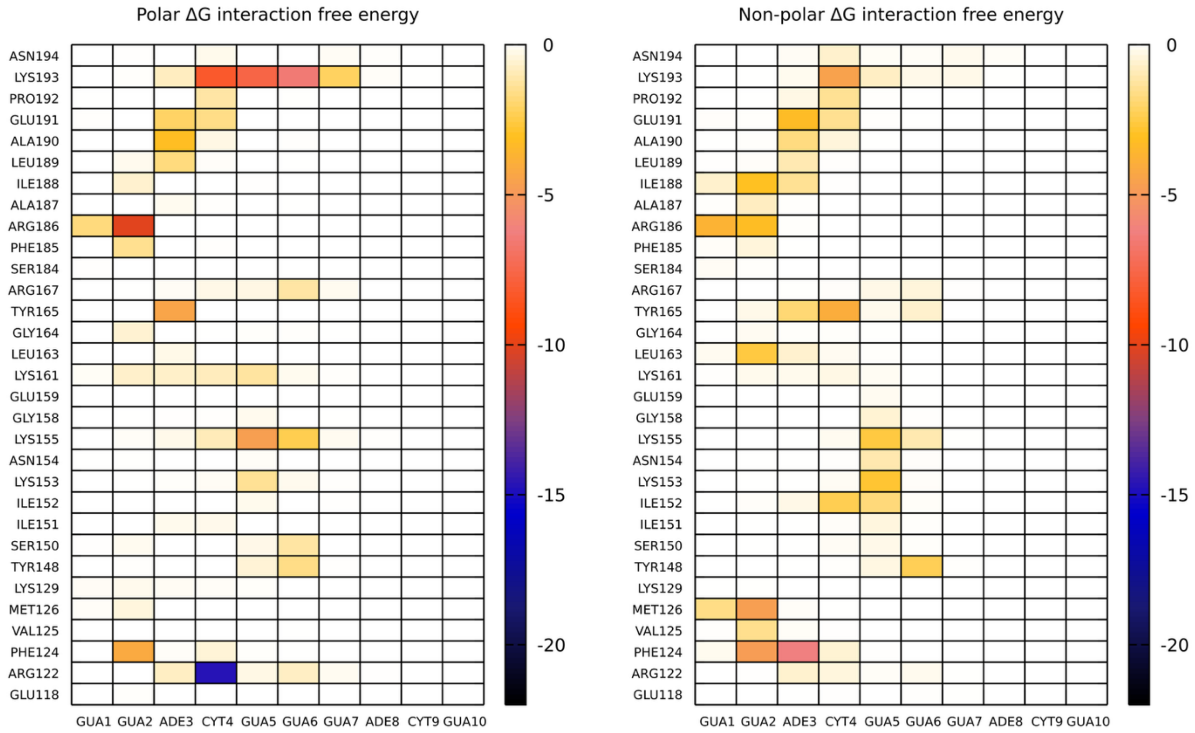

Figure S13. A. Residue-nucleotide pairwise ΔG interaction free energy (kcal/mol) plot for nucleotide (x-axis) and protein residue (y-axis) pairs calculated for 5′-GG-m^{6}A-CAGGACG-3′ in complex with RRM2 of RBM45. The plots represent favorable (< 0 kcal/mol) average polar and non-polar ΔG interaction free energies. **B.** Residue-nucleotide pairwise ΔΔG interaction free energy (kcal/mol) plot for nucleotide (x-axis) and protein residue (y-axis) pairs for 5′-GG-m^{6}A-CAGGACG-3′ compared to 5′-GGACAGGACG-3′ in complex with RRM2 of RBM45. The plots represent ΔΔG interaction free energy of both favorable (< 0 kcal/mol) and unfavorable (>0 kcal/mol) average polar and non-polar ΔG interaction free energies for 5′-GG-m^{6}A-CAGGACG-3′ – 5′-GGACAGGACG-3′. The ΔG and ΔΔG interaction free energy (kcal/mol)-color correspondence is shown on the palette on the right of each plot.

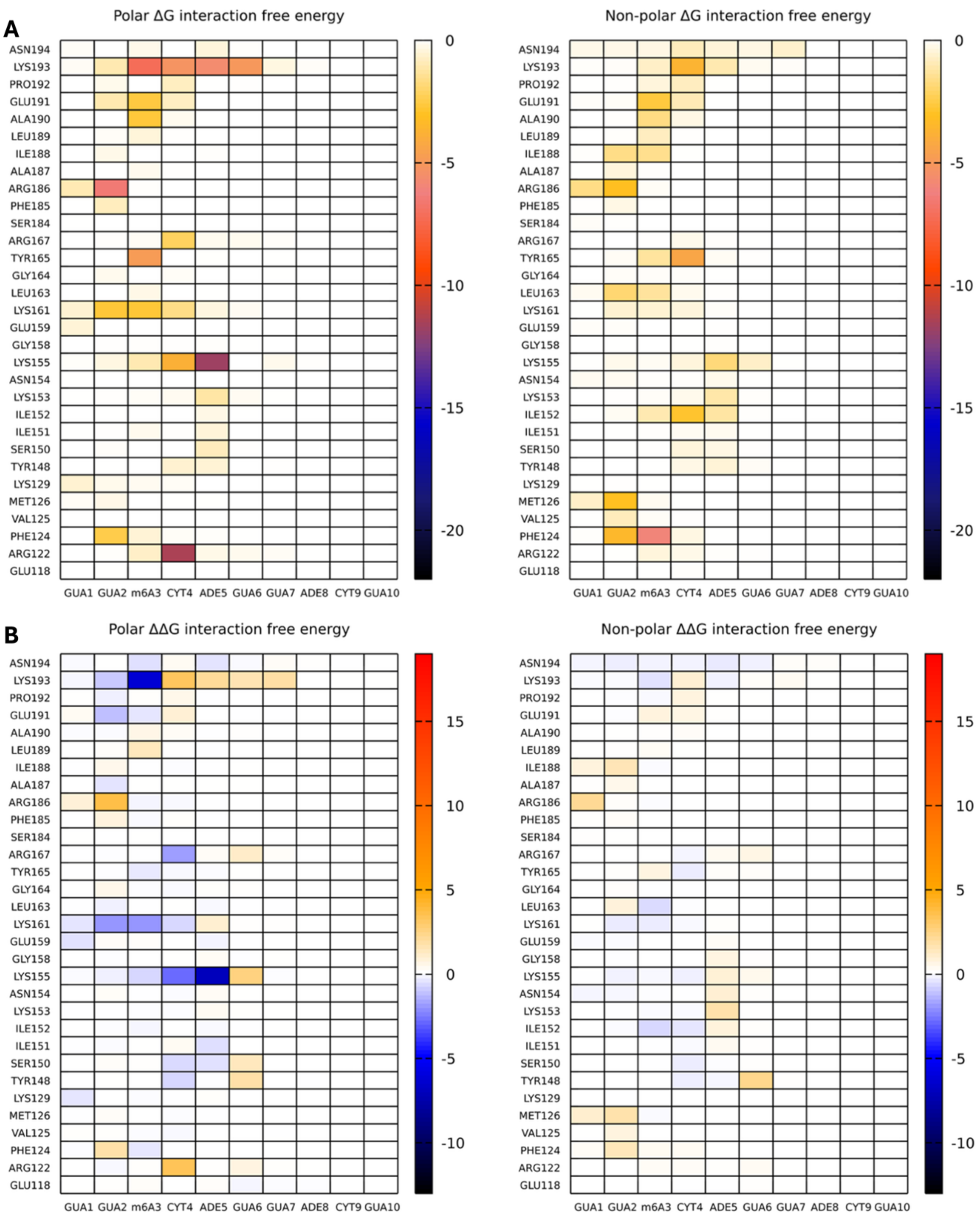

Figure S14. Residue-nucleotide pairwise ΔG interaction free energy (kcal/mol) plot for nucleotide (x-axis) and protein residue (y-axis) pairs calculated for 5′-GGACUGGACG-3′ in complex with RRM2 of RBM45. The plots represent favorable (< 0 kcal/mol) average polar and non-polar ΔG interaction free energies. The ΔG interaction free energy (kcal/mol)-color correspondence is shown on the palette on the right of each plot.

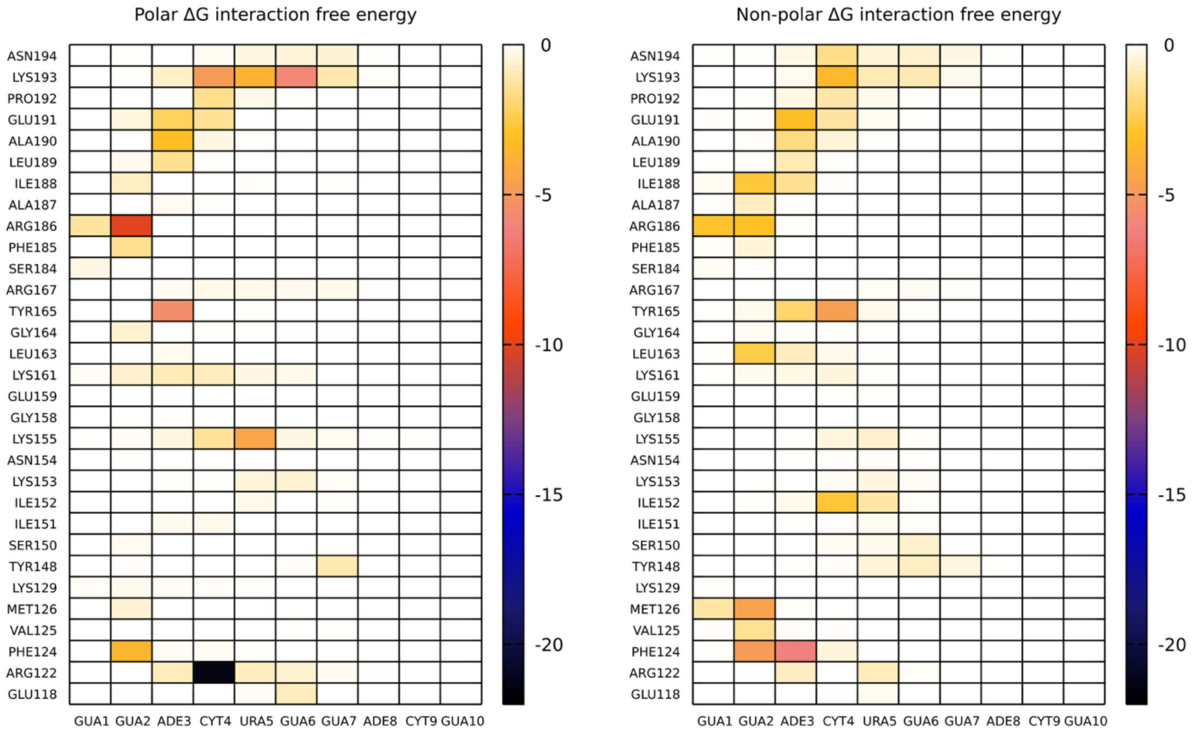

Figure S15. ΔG interaction free energy (kcal/mol) (y-axis) per nucleotide plot calculated for four RNA sequences (x-axis) in complex with RRM2 of RBM45. Purple bars represent polar ΔG interaction free energy (kcal/mol) per nucleotide, and green bars represent non-polar ΔG interaction free energy (kcal/mol) per nucleotide. **A.** 5′-GGACAGGACG-3′ (Entry 8) in complex with RRM2 of RBM45. **B.** 5′-GGACUGGACG-3′ (Entry 10) in complex with RRM2 of RBM45. **C.** 5′-GG-m^{6}A-CAGGACG-3′ (Entry 9) in complex with RRM2 of RBM45. **D.** 5′-GG-m^{6}A-CUGGACG-3′ (Entry 11) in complex with RRM2 of RBM45.

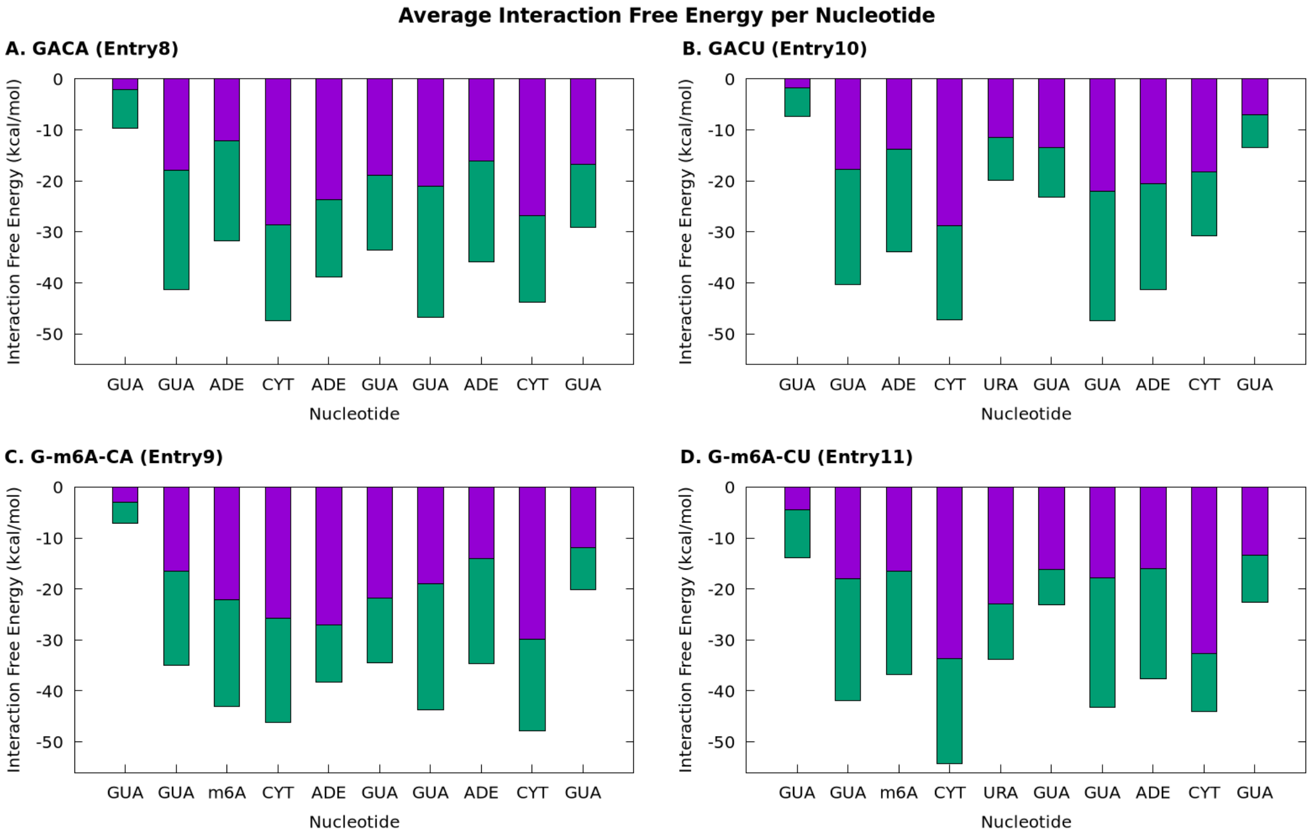

Figure S16. A. Residue-nucleotide pairwise ΔG interaction free energy (kcal/mol) plot for nucleotide (x-axis) and protein residue (y-axis) pairs calculated for 5′-GG-m^{6}A-CUGGACG-3′ in complex with RRM2 of RBM45. The plots represent favorable (< 0 kcal/mol) average polar and non-polar ΔG interaction free energies. **B.** Residue-nucleotide pairwise ΔΔG interaction free energy (kcal/mol) plot for nucleotide (x-axis) and protein residue (y-axis) pairs for 5′-GG-m^{6}A-CUGGACG-3′ compared to 5′-GGACUGGACG-3′ in complex with RRM2 of RBM45. The plots represent ΔΔG interaction free energy of both favorable (< 0 kcal/mol) and unfavorable (>0 kcal/mol) average polar and non-polar ΔG interaction free energies for 5′-GG-m^{6}A-CUGGACG-3′ – 5′-GGACUGGACG-3′. The ΔG and ΔΔG interaction free energy (kcal/mol)-color correspondence is shown on the palette on the right of each plot.

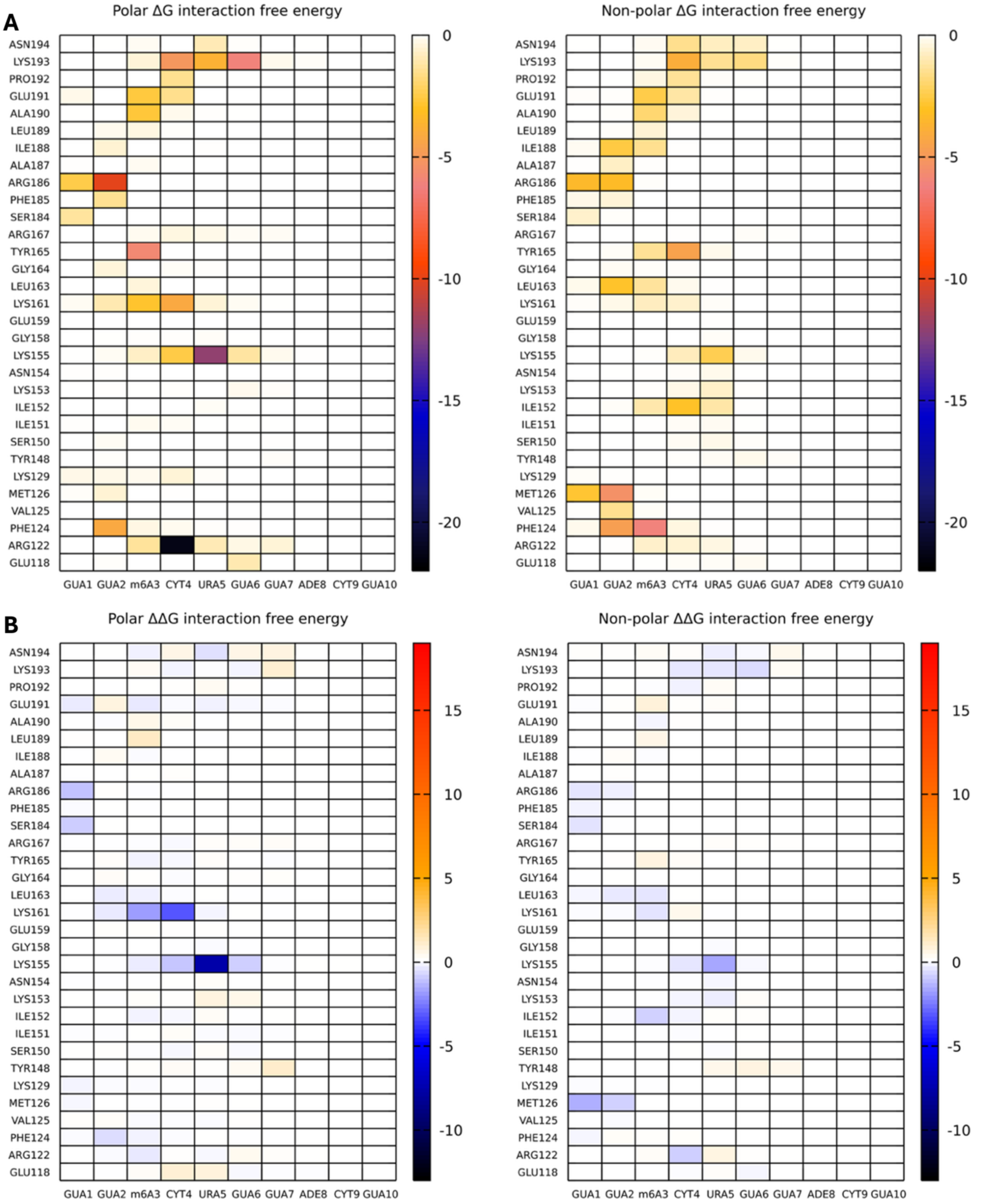

Figure S17. ΔG interaction free energy (kcal/mol) (y-axis) per nucleotide plot calculated for six RNA sequences (x-axis) in complex with RRM3 of RBM45. Purple bars represent polar ΔG interaction free energy (kcal/mol) per nucleotide, and green bars represent non-polar ΔG interaction free energy (kcal/mol) per nucleotide. **A.** 5′-GACAGG-3′ (Entry 14) in complex with RRM3 of RBM45. **B.** 5′-GACUGG-3′ (Entry 16) in complex with RRM3 of RBM45. **C.** 5′-G-m^{6}A-CAGG-3′ (Entry 15) in complex with RRM3 of RBM45. **D.** 5′-G-m^{6}A-CUGG-3′ (Entry 17) in complex with RRM3 of RBM45.

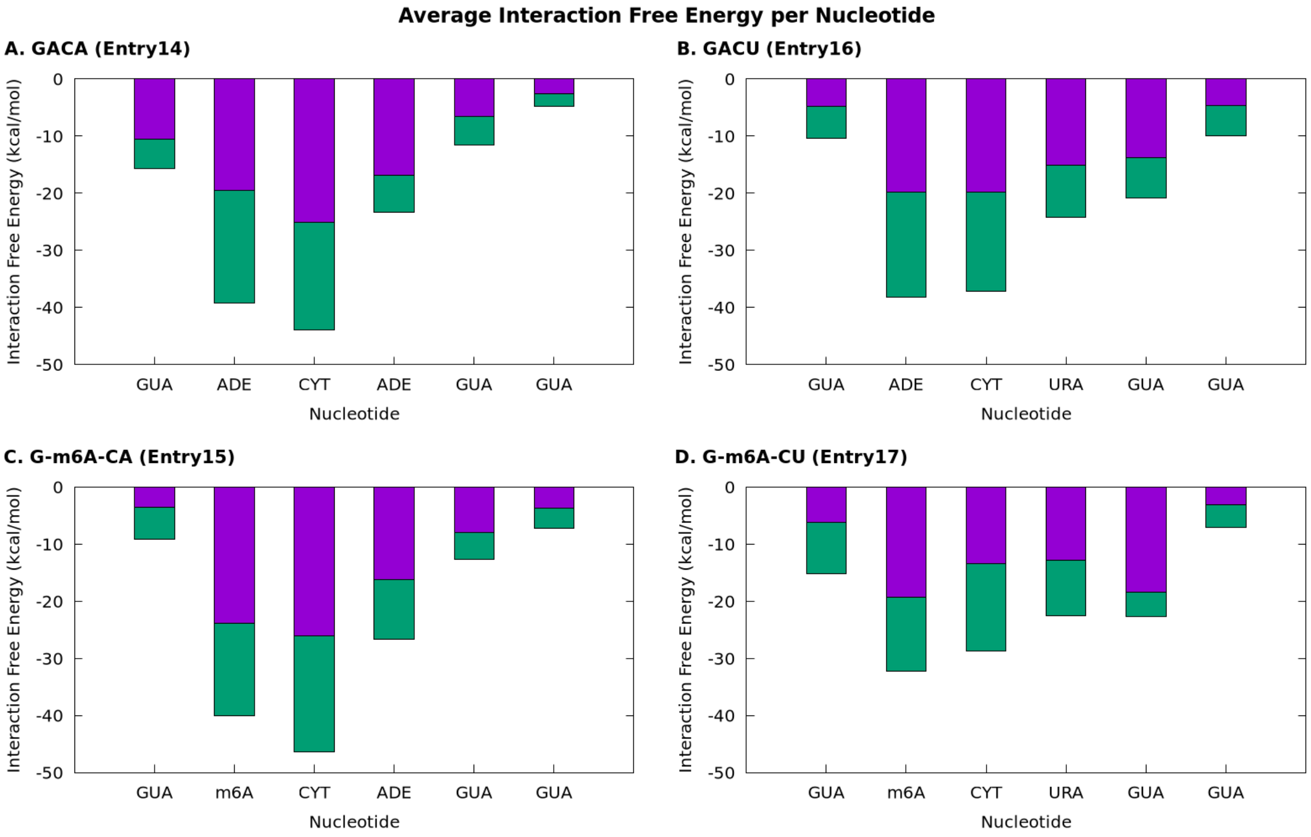

Figure S18. A. Residue-nucleotide pairwise ΔG interaction free energy (kcal/mol) plot for nucleotide (x-axis) and protein residue (y-axis) pairs calculated for 5′-GACAGG-3′ in complex with RRM3 of RBM45. The plots represent favorable (< 0 kcal/mol) average polar and non-polar ΔG interaction free energies. The ΔG interaction free energy (kcal/mol)-color correspondence is shown on the palette on the right of each plot. **B.** Residue-nucleotide pairwise ΔG interaction free energy (kcal/mol) plot for nucleotide (x-axis) and protein residue (y-axis) pairs calculated for 5′-GACUGG-3′ in complex with RRM3 of RBM45. The plots represent favorable (< 0 kcal/mol) average polar and non-polar ΔG interaction free energies. The ΔG interaction free energy (kcal/mol)-color correspondence is shown on the palette on the right of each plot.

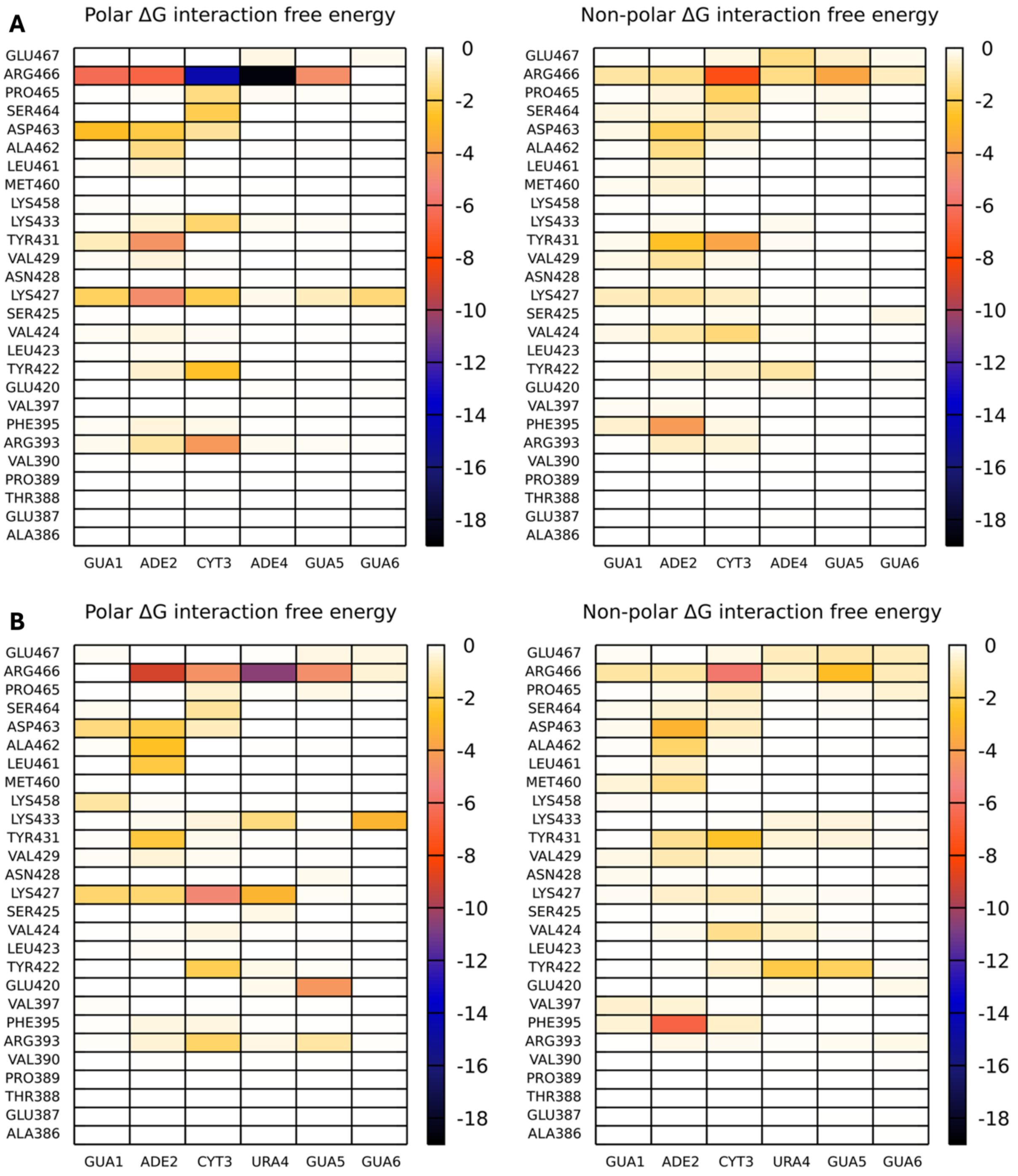

Figure S19. A. Residue-nucleotide pairwise ΔG interaction free energy (kcal/mol) plot for nucleotide (x-axis) and protein residue (y-axis) pairs calculated for 5′-G-m^{6}A-CAGG-3′ in complex with RRM3 of RBM45. The plots represent favorable (< 0 kcal/mol) average polar and non-polar ΔG interaction free energies. **B.** Residue-nucleotide pairwise ΔΔG interaction free energy (kcal/mol) plot for nucleotide (x-axis) and protein residue (y-axis) pairs for 5′-G-m^{6}A-CAGG-3′ compared to 5′-GACAGG-3′ in complex with RRM3 of RBM45. The plots represent ΔΔG interaction free energy of both favorable (< 0 kcal/mol) and unfavorable (>0 kcal/mol) average polar and non-polar ΔG interaction free energies for 5′-G-m^{6}A-CAGG-3′ – 5′-GACAGG-3′. The ΔG and ΔΔG interaction free energy (kcal/mol)-color correspondence is shown on the palette on the right of each plot.

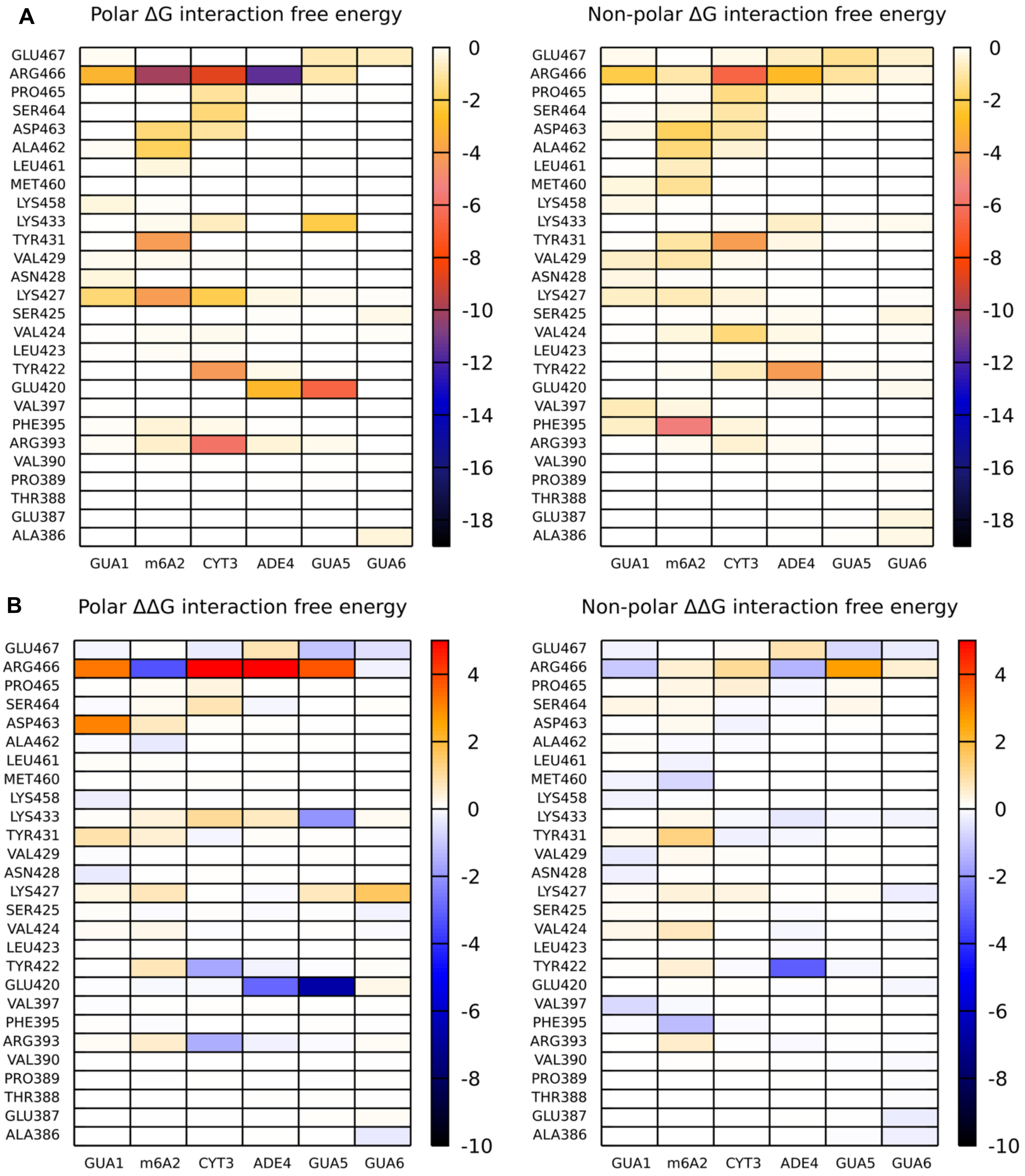

Figure S20. A. Residue-nucleotide pairwise ΔG interaction free energy (kcal/mol) plot for nucleotide (x-axis) and protein residue (y-axis) pairs calculated for 5′-G-m^{6}A-CUGG-3′ in complex with RRM3 of RBM45. The plots represent favorable (< 0 kcal/mol) average polar and non-polar ΔG interaction free energies. **B.** Residue-nucleotide pairwise ΔΔG interaction free energy (kcal/mol) plot for nucleotide (x-axis) and protein residue (y-axis) pairs for 5′-G-m^{6}A-CUGG-3′ compared to 5′-GACUGG-3′ in complex with RRM3 of RBM45. The plots represent ΔΔG interaction free energy of both favorable (< 0 kcal/mol) and unfavorable (>0 kcal/mol) average polar and non-polar ΔG interaction free energies for 5′-G-m^{6}A-CUGG-3′ – 5′-GACUGG-3′. The ΔG and ΔΔG interaction free energy (kcal/mol)-color correspondence is shown on the palette on the right of each plot.

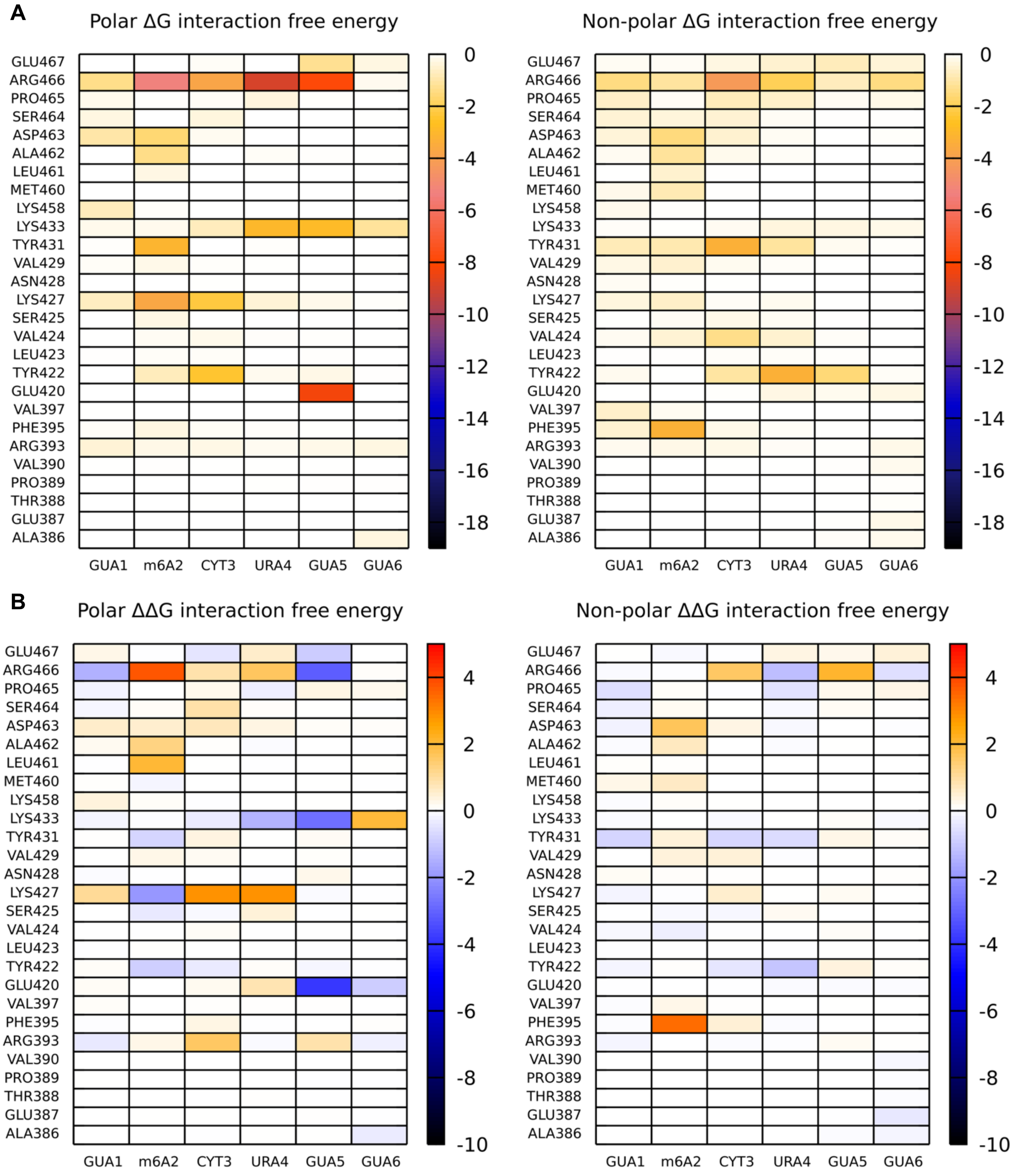

Figure S21. A. Clustering analysis. The percentage of each binding conformation adopted by the RNA strand in complex with the RRM3 domain is shown. Cluster 0 consists of structures not assigned to any specific cluster. **B**. For each cluster, the RMSD of the structure representing the cluster center was calculated with respect to the backbone atoms of the reference conformation, the minimum binding free energy snapshot of 5′-GACGGA-3′ RNA in complex with RRM3. Prior to the RMSD calculation, the trajectories were structurally aligned to the backbone atoms of the protein and RNA. The reference RNA conformation of 5′-GACGGA-3′ is shown in both panels **C** and **D,** in white tube for comparison. **C**. Cluster centers for clusters 1, 2, 5, 6, 7, 8, and 9 are presented; these clusters represent conformations in which the RNA strand is considered bound to the protein. The unmodified and m⁶A-modified 5′-GACGGG-3′ in complex with the RRM3 domain primarily populated these clusters. **D**. Cluster centers for clusters 3, 4, 10, and 11 are shown; these clusters reflect conformations in which the RNA strand is considered either loosely bound or associated with an alternative binding pocket. The unmodified and m⁶A-modified 5′-GACAGG-3′ and 5′-GACUGG-3′ in complex with RRM3 primarily populated these clusters.

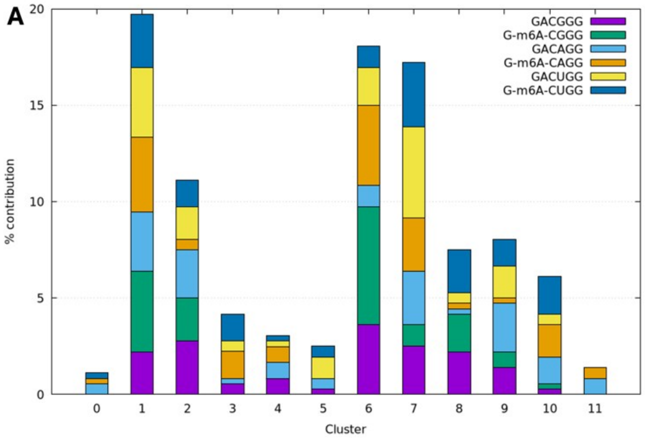

B

Bound conformation	
**Cluster**	**RMSD (Å)**
1	3.00
2	2.69
5	2.56
6	2.50
7	3.40
8	3.83
9	2.04
**Loosely bound/alternative binding pocket**	
**Cluster**	**RMSD (Å)**
3	5.17
4	11.27
10	5.49
11	19.43

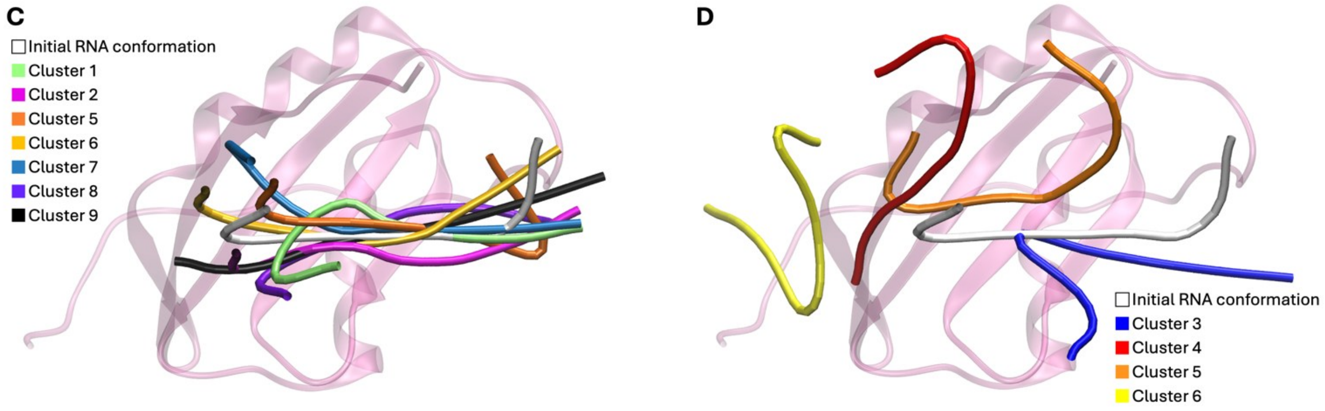

Figure S22. Root mean square deviation (RMSD) for RRM3:full-length RBM45:RNA complexes performed for the entire 1-300 ns time range. Trajectories were aligned to backbone atoms of the entire protein and the bound RNA. RMSD values (Å) were calculated with respect to the refined structure which was used as a common starting point conformation for the four GACA, G-m^{6}A-CA, GACU, and G-m^{6}A-CU systems investigated. Average and standard deviation values across different runs are depicted in dark and light blue, respectively.

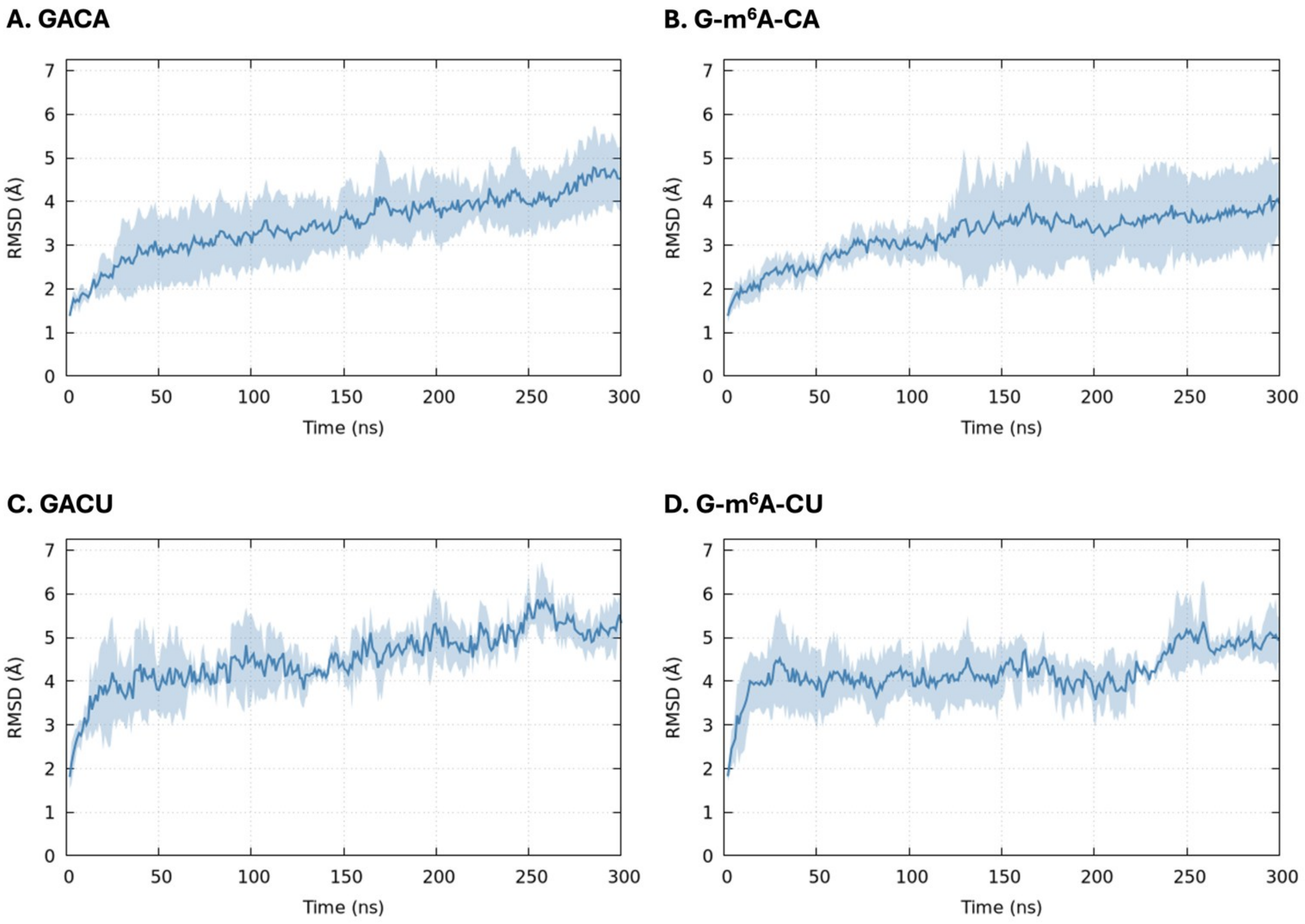

Figure S23. A. Residue-nucleotide pairwise ΔG interaction free energy (kcal/mol) plot for nucleotide (x-axis) and protein residue (y-axis) pairs calculated for 5′-G-m[6]A-CAGG-3′ in complex with RRM3 domain in the context of full-length RBM45 protein. The plots represent favorable (< 0 kcal/mol) average polar and non-polar ΔG interaction free energies. **B.** Residue-nucleotide pairwise ΔG interaction free energy (kcal/mol) plot for nucleotide (x-axis) and protein residue (y-axis) pairs calculated for 5′-GACAGG-3′ in complex with RRM3 domain in the context of full-length RBM45. The plots represent favorable (< 0 kcal/mol) average polar and non-polar ΔG interaction free energies. The ΔG interaction free energy (kcal/mol)-color correspondence is shown on the palette on the right of each plot.

A

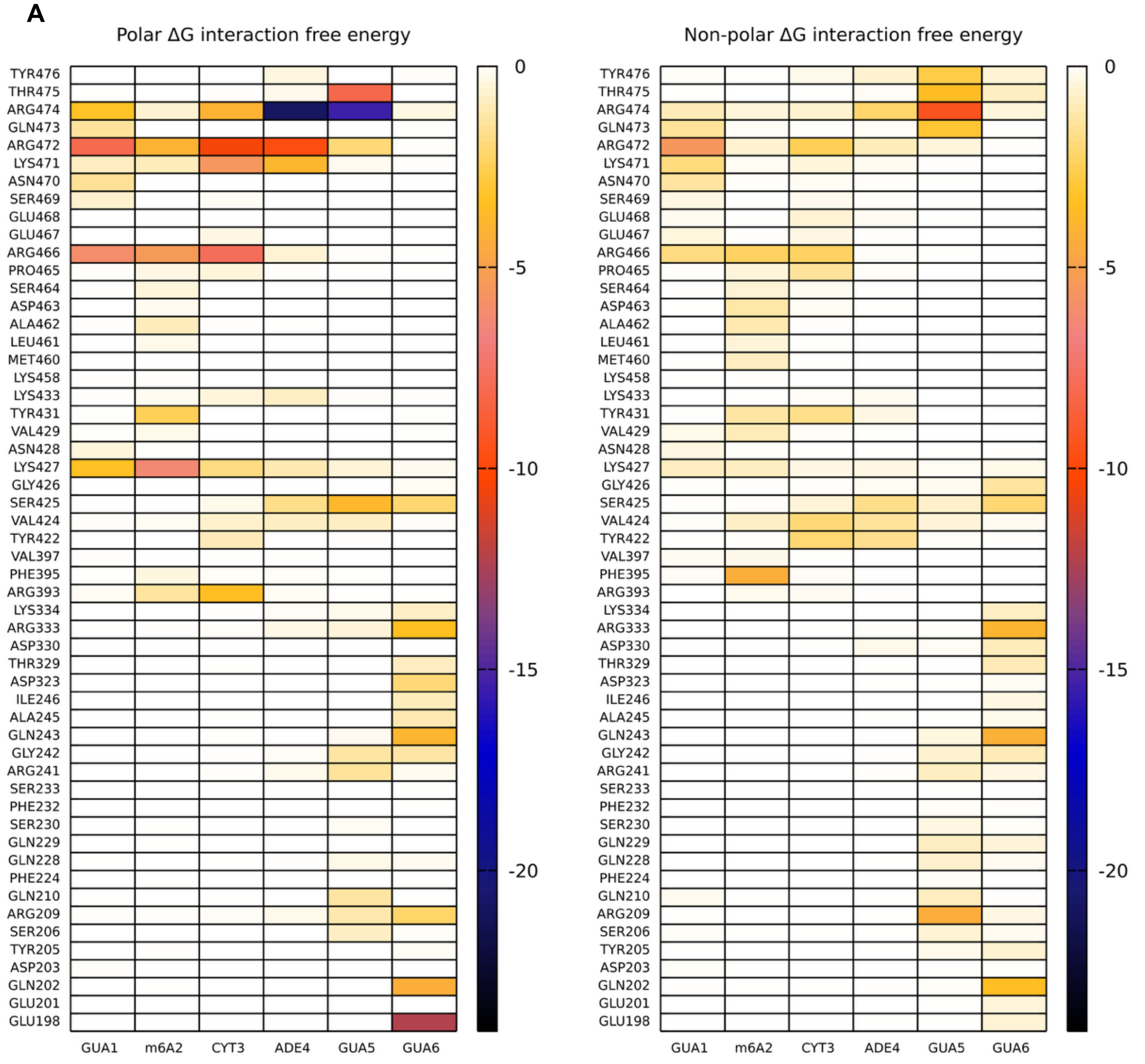

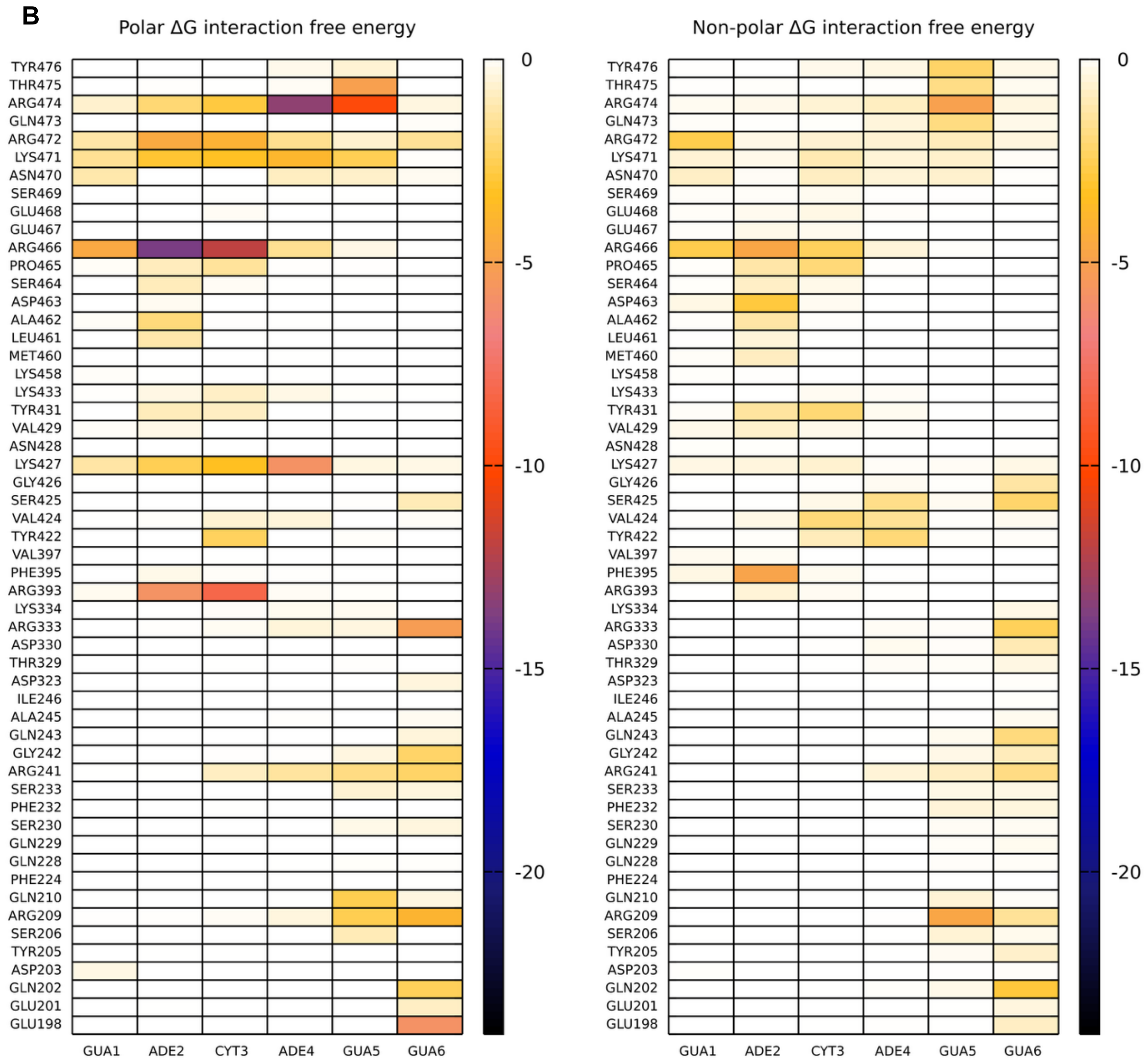
B
Polar ΔG interaction free energy
Non-polar ΔG interaction free energy
TYR476
THR475
ARG474
GLN473
ARG472
LYS471
ASN470
SER469
GLU468
GLU467
ARG466
PRO465
SER464
ASP463
ALA462
LEU461
MET460
LYS458
LYS433
TYR431
VAL429
ASN428
LYS427
GLY426
SER425
VAL424
TYR422
VAL397
PHE395
ARG393
LYS334
ARG333
ASP330
THR329
ASP323
ILE246
ALA245
GLN243
GLY242
ARG241
SER233
PHE232
SER230
GLN229
GLN228
PHE224
GLN210
ARG209
SER206
TYR205
ASP203
GLN202
GLU201
GLU198
GUA1
ADE2
CYT3
ADE4
GUA5
GUA6
0
-5
-10
-15
-20

Figure S24. A-B. Time evolution of the distance (Å) between the negatively charged Y476 in the C-terminal domain and the positively charged R241 in the linker domain between RRM2-HOA domain during MD simulations of RNA-protein complexes in the context of the full-length RBM45 protein. The plots show the distance between the indicated atom pairs measured across six independent trajectories (200 ns each). In each panel, the blue line corresponds to the distances for the protein residue and protein residue pair, Y476-R241. **A.** 5′-G-m^{6}A-CAGG-3′ in complex with RRM3 in the context of the full-length RBM45 protein. **B.** 5′-GACAGG-3′ in complex with RRM3 in the context of the full-length RBM45 protein.

A. G-m^{6}A-CAGG

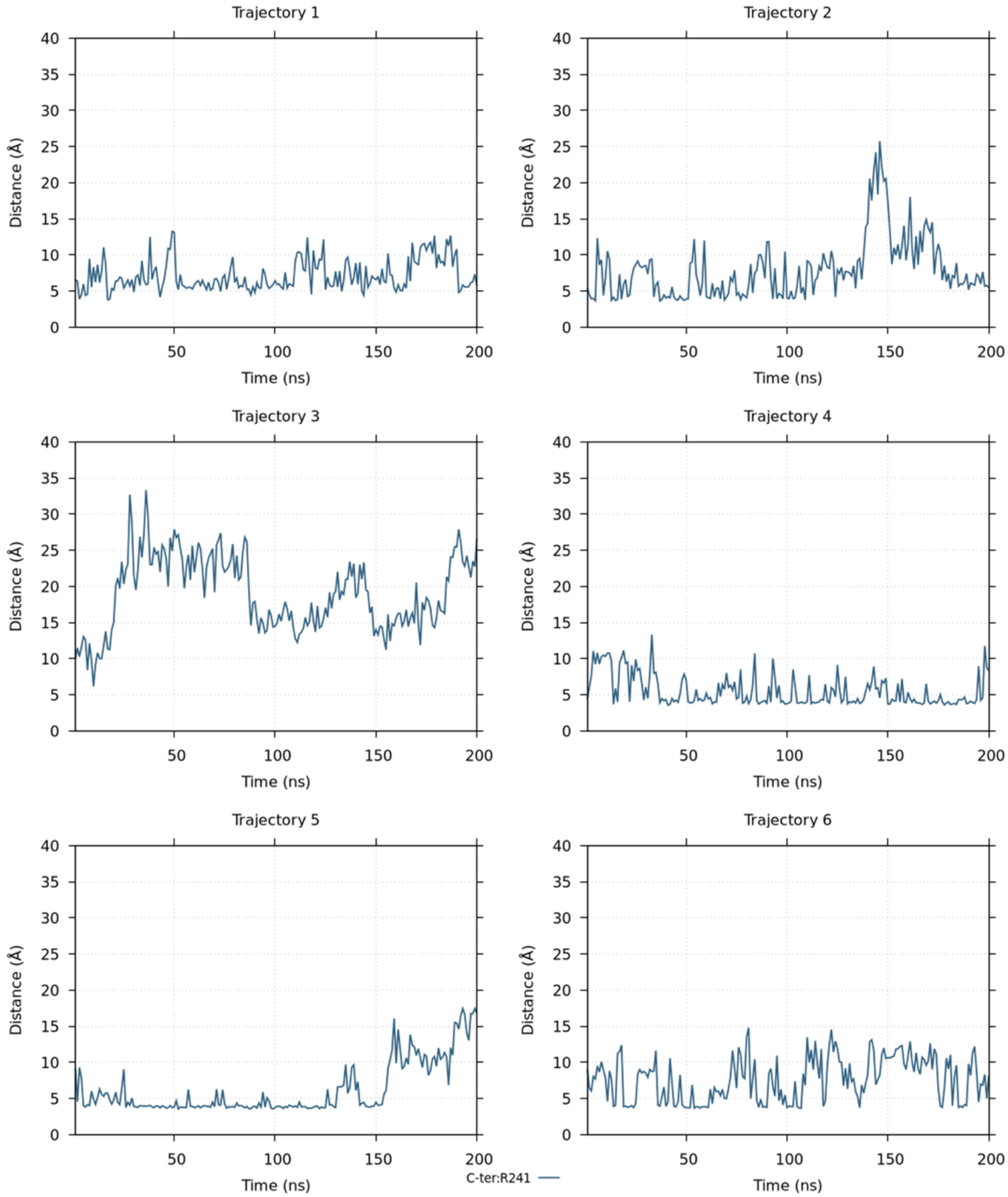

B. GACAGG

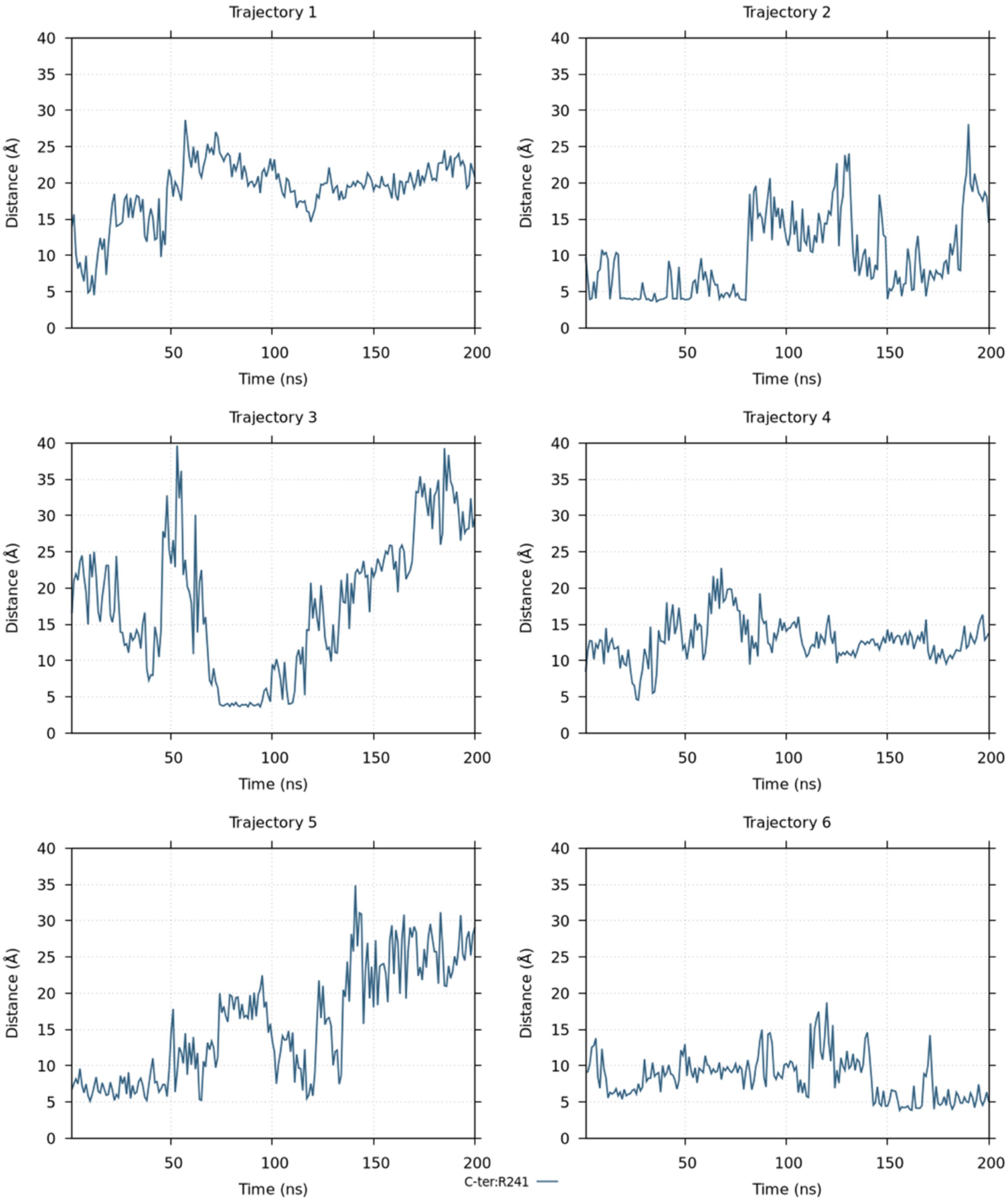
Trajectory 1
Trajectory 2
Trajectory 3
Trajectory 4
Trajectory 5
Trajectory 6
Distance (Å)
Time (ns)
0
5
10
15
20
25
30
35
40
50
100
150
200
C-ter:R241

Figure S25. Residue-nucleotide pairwise ΔΔG interaction free energy (kcal/mol) plot for nucleotide (x-axis) and protein residue (y-axis) pairs for 5′-G-m⁶A-CAGG-3′ compared to 5′-GACAGG-3′ in complex with RRM3 in the context of full-length RBM45 protein. The plots represent ΔΔG interaction free energy of both favorable (< 0 kcal/mol) and unfavorable (>0 kcal/mol) average polar and non-polar ΔG interaction free energies for 5′-G-m⁶A-CAGG-3′ – 5′-GACAGG-3′. The ΔG and ΔΔG interaction free energy (kcal/mol)-color correspondence is shown on the palette on the right of each plot.

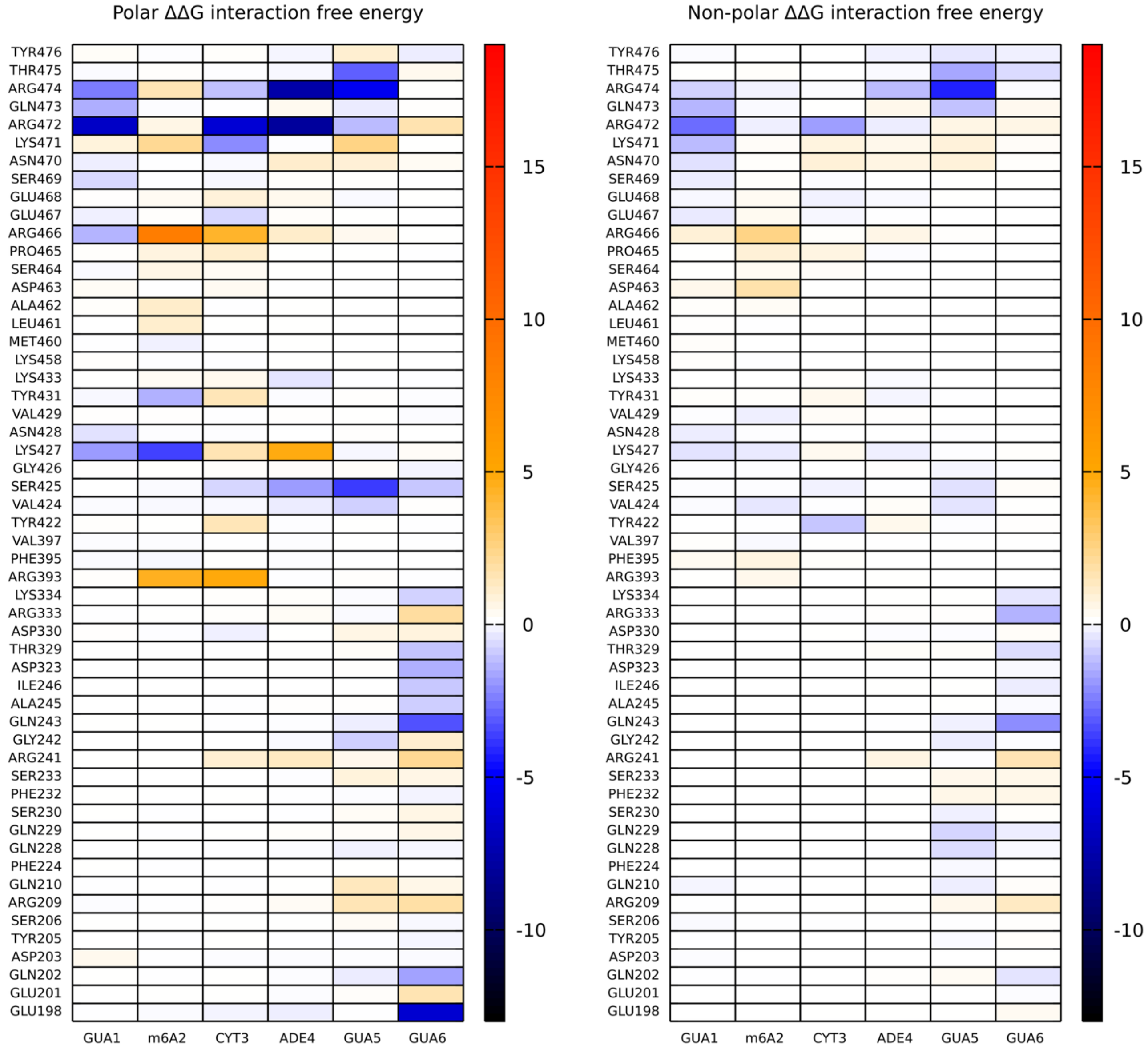

Figure S26. ΔG interaction free energy (kcal/mol) (y-axis) per nucleotide plot calculated for six RNA sequences (x-axis) in complex with RRM3 in the context of full-length RBM45 protein. Purple bars represent polar ΔG interaction free energy (kcal/mol) per nucleotide, and green bars represent non-polar ΔG interaction free energy (kcal/mol) per nucleotide. **A.** 5′-G-m^6A-CAGG-3′ (Entry 19) in complex with RRM3 in the context of full-length RBM45 protein. **B.** 5′-GACAGG-3′ (Entry 18) in complex with RRM3 in the context of full-length RBM45 protein. **C.** 5′-G-m^6A-CUGG-3′ (Entry 21) in complex with RRM3 in the context of full-length RBM45 protein. **D.** 5′-GACUGG-3′ (Entry 20) in complex with RRM3 in the context of full-length RBM45 protein.

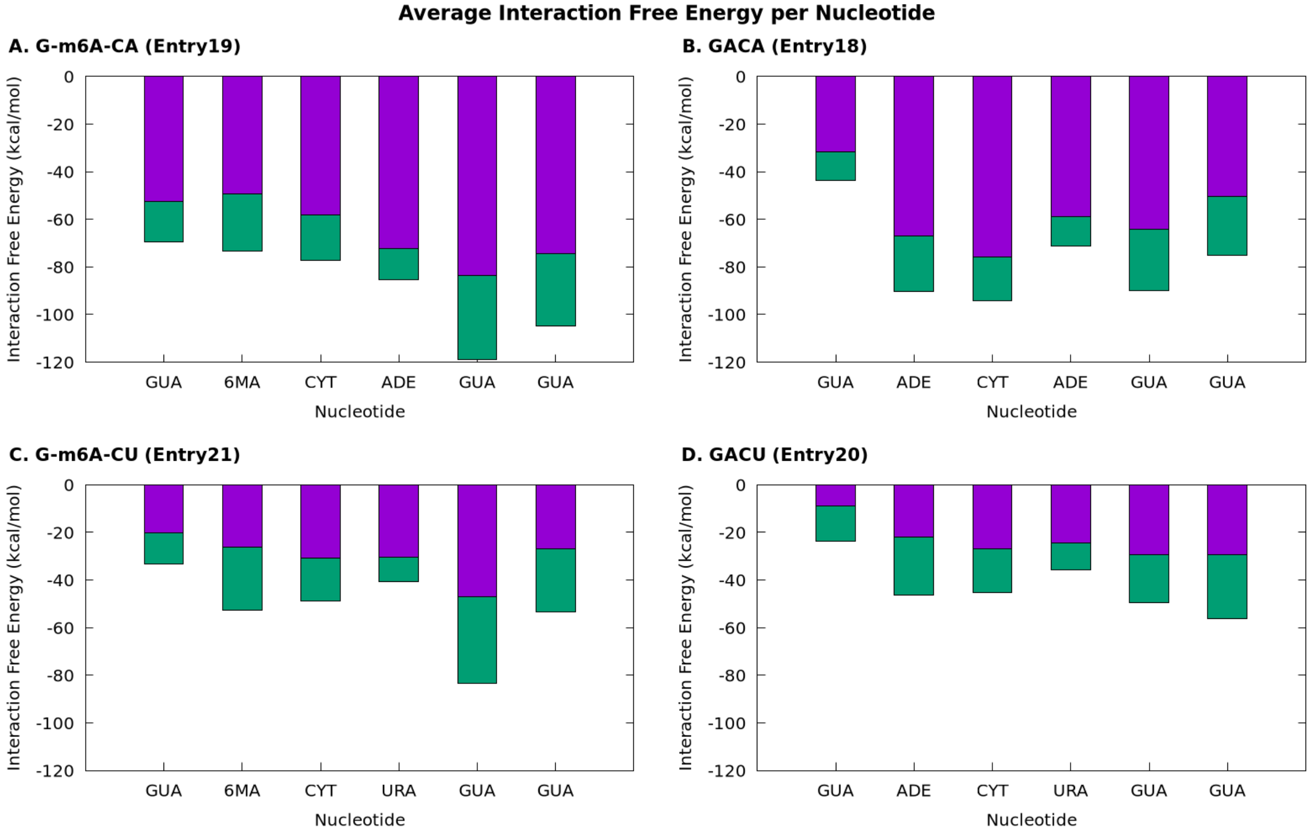

Figure S27. Residue-nucleotide pairwise ΔG interaction free energy (kcal/mol) plot for nucleotide (x-axis) and protein residue (y-axis) pairs calculated for 5′-GACUGG-3′ in complex with RRM3 in the context of full-length RBM45. The plots represent favorable (< 0 kcal/mol) average polar and non-polar ΔG interaction free energies. The ΔG interaction free energy (kcal/mol)-color correspondence is shown on the palette on the right of each plot.

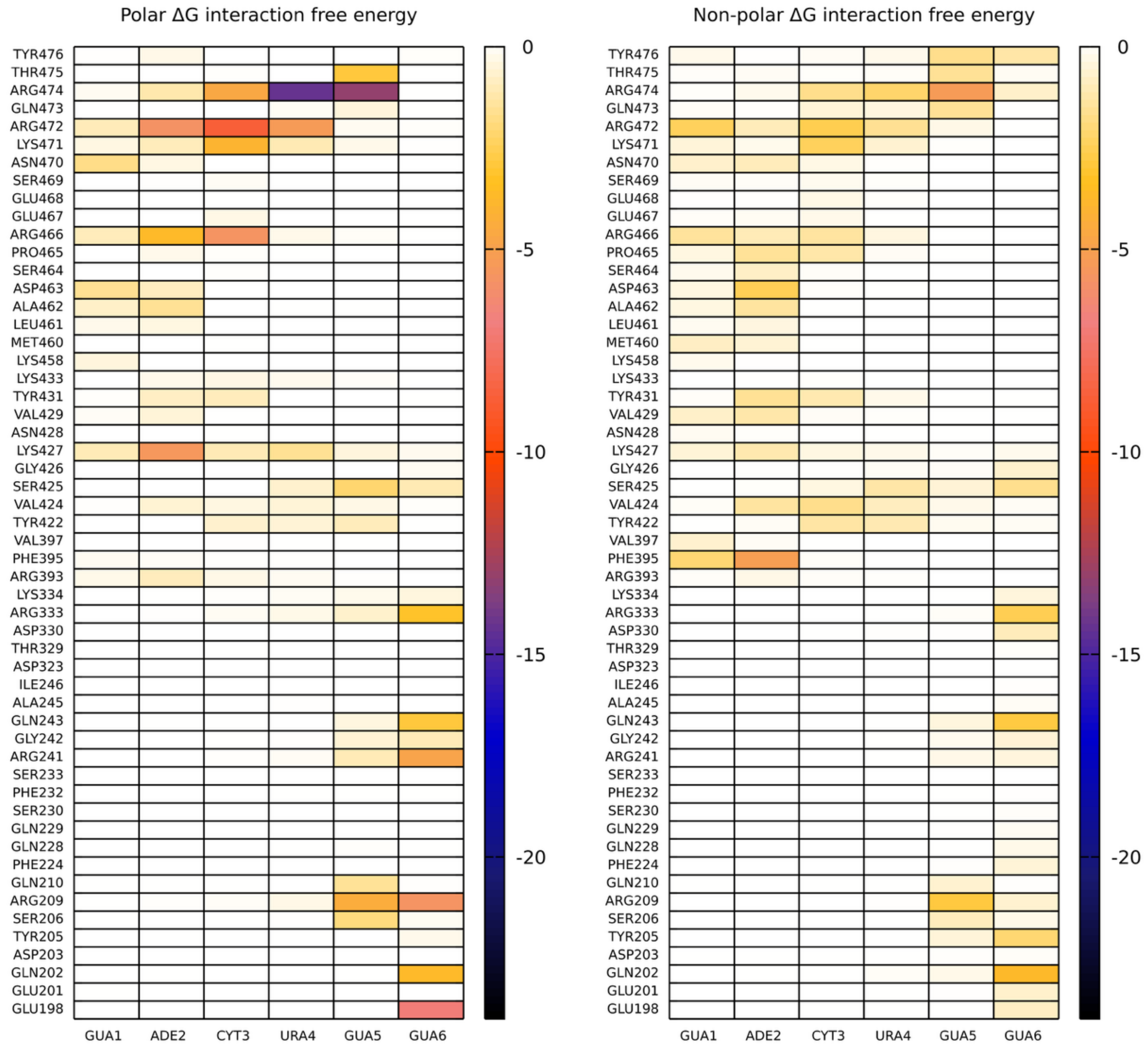

Figure S28. Residue-nucleotide pairwise ΔG interaction free energy (kcal/mol) plot for nucleotide (x-axis) and protein residue (y-axis) pairs calculated for 5′-G-m^{6}A-CUGA-3′ in complex with RRM3 in the context of full-length RBM45 protein. The plots represent favorable (< 0 kcal/mol) average polar and non-polar ΔG interaction free energies.

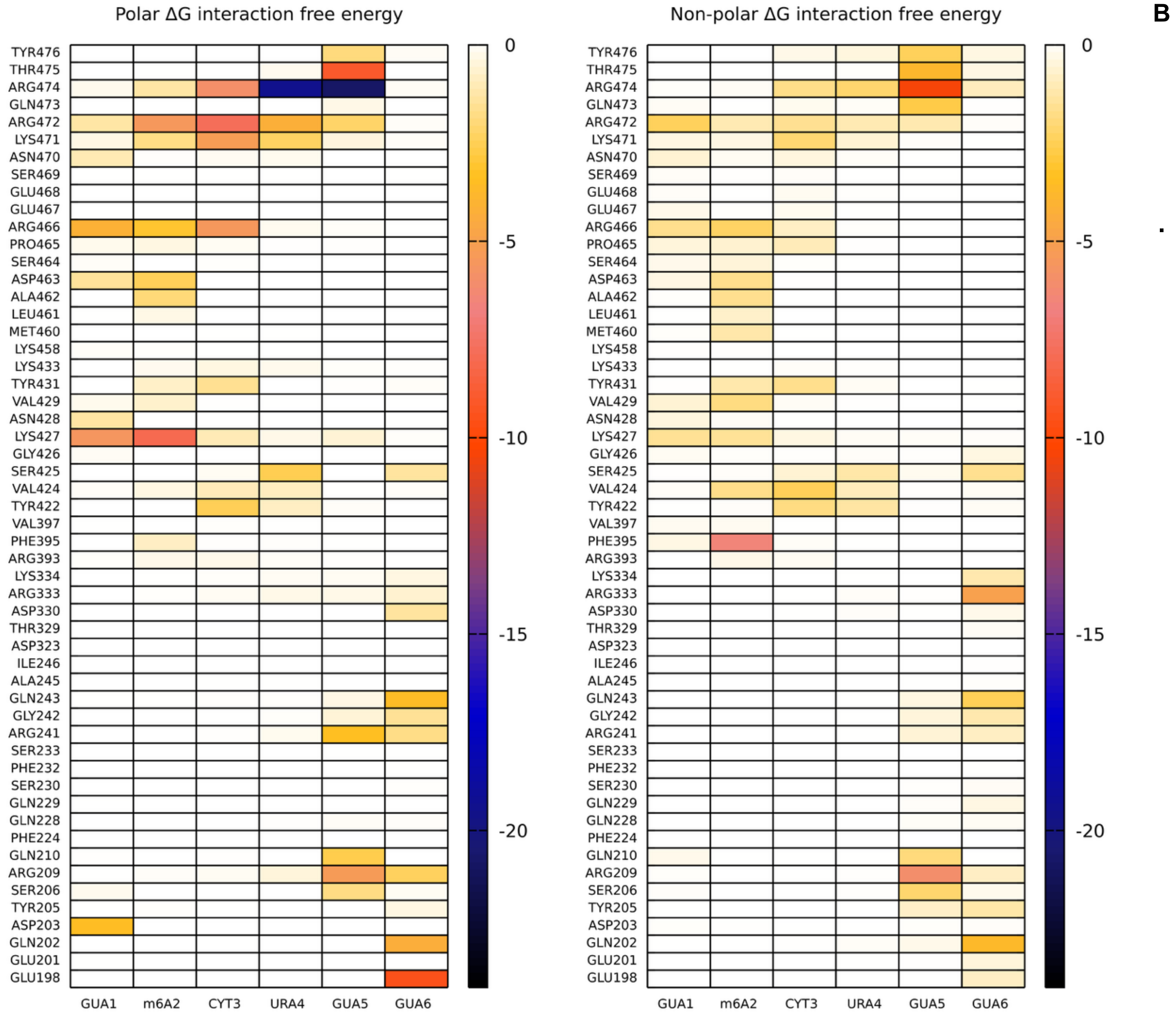

Figure S29. Residue-nucleotide pairwise ΔΔG interaction free energy (kcal/mol) plot for nucleotide (x-axis) and protein residue (y-axis) pairs for 5′-G-m⁶A-CUGA-3′ compared to 5′-GACUGA-3′ in complex with RRM3 in the context of RBM45. The plots represent ΔΔG interaction free energy of both favorable (< 0 kcal/mol) and unfavorable (>0 kcal/mol) average polar and non-polar ΔG interaction free energies for 5′-G-m⁶A-CUGA-3′ – 5′-GACUGA-3′. The ΔG and ΔΔG interaction free energy (kcal/mol)-color correspondence is shown on the palette on the right of each plot.

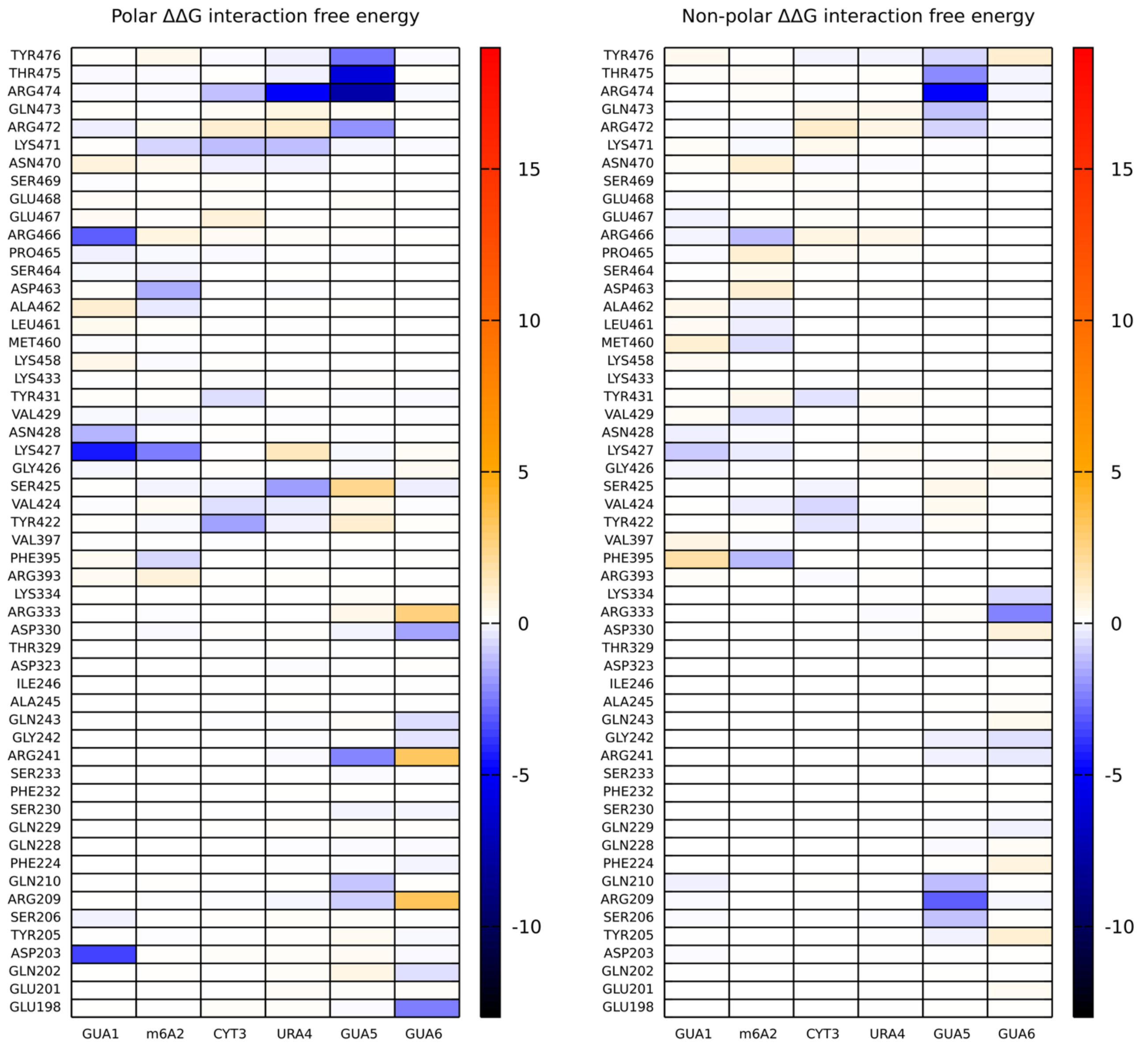

Figure S30. A-B. Time evolution of the distance (Å) between the negatively charged Y476 in the C-terminal domain and the positively charged R241 in the linker domain between RRM2-HOA domain during MD simulations of RNA-protein complexes in the context of the full-length RBM45 protein. The plots show the distance between the indicated atom pairs measured across three independent trajectories (200 ns each). In each panel, the blue line corresponds to the distances for the protein residue and protein residue pair, Y476-R241. **A.** 5′-G-m^6A-CUGG-3′ in complex with RRM3 in the context of the full-length RBM45 protein. **B.** 5′-GACUGG-3′ in complex with RRM3 in the context of the full-length RBM45 protein.

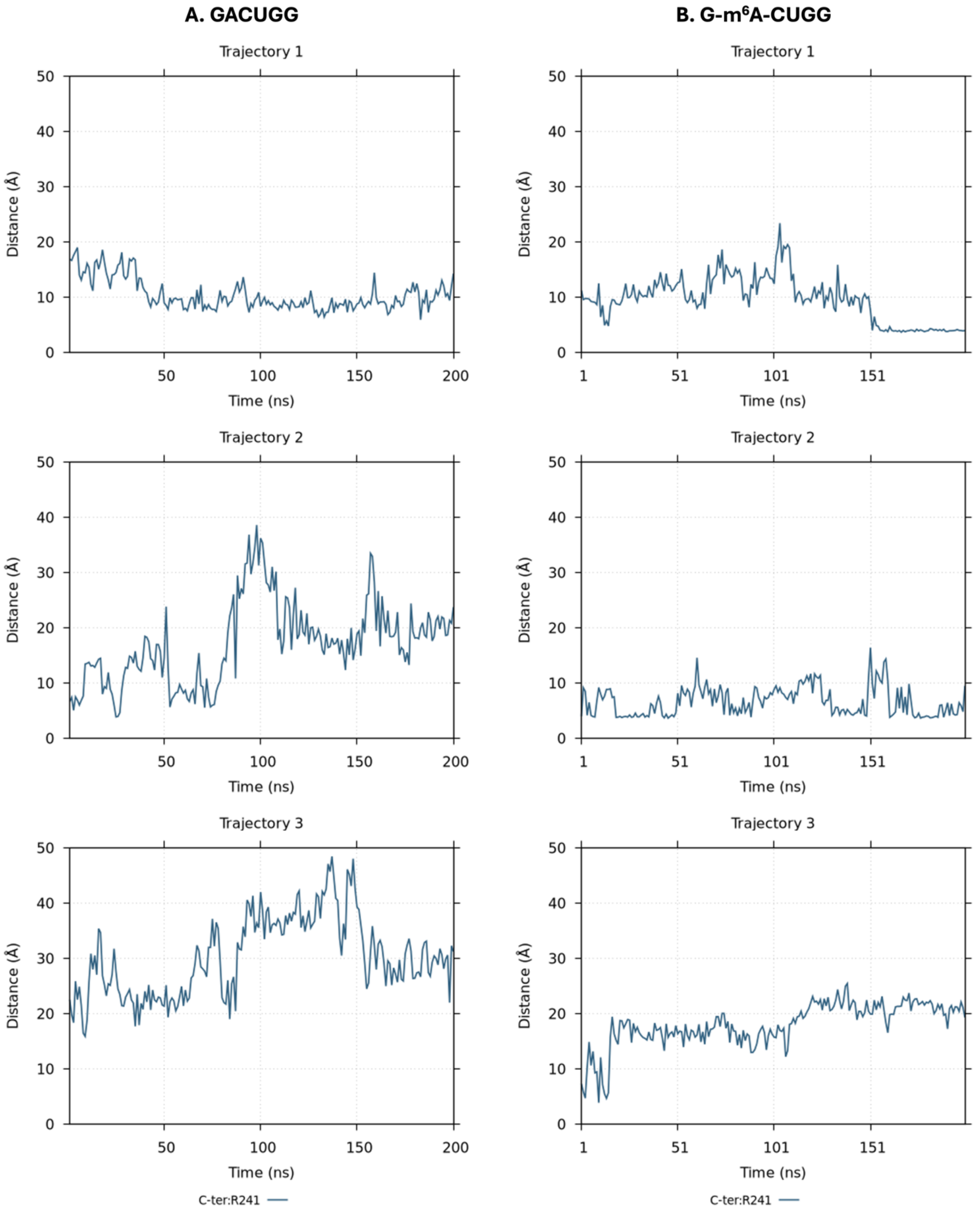

Figure S31. Time evolution of the distance (Å) between M460:SD-m⁶A:CM6 and F395:CG-m⁶A:CM6 during MD simulation of 5′-G-m⁶A-CAGG-3′ in complex with RRM3 domain in the context of full-length RBM45 protein. The plots show the distance between the indicated atom pairs measured across six independent trajectories (200 ns each). In each panel, the purple and blue line correspond to the distances for the two-protein residues and m⁶A pairs, M460-m⁶A and F395-m⁶A, respectively.

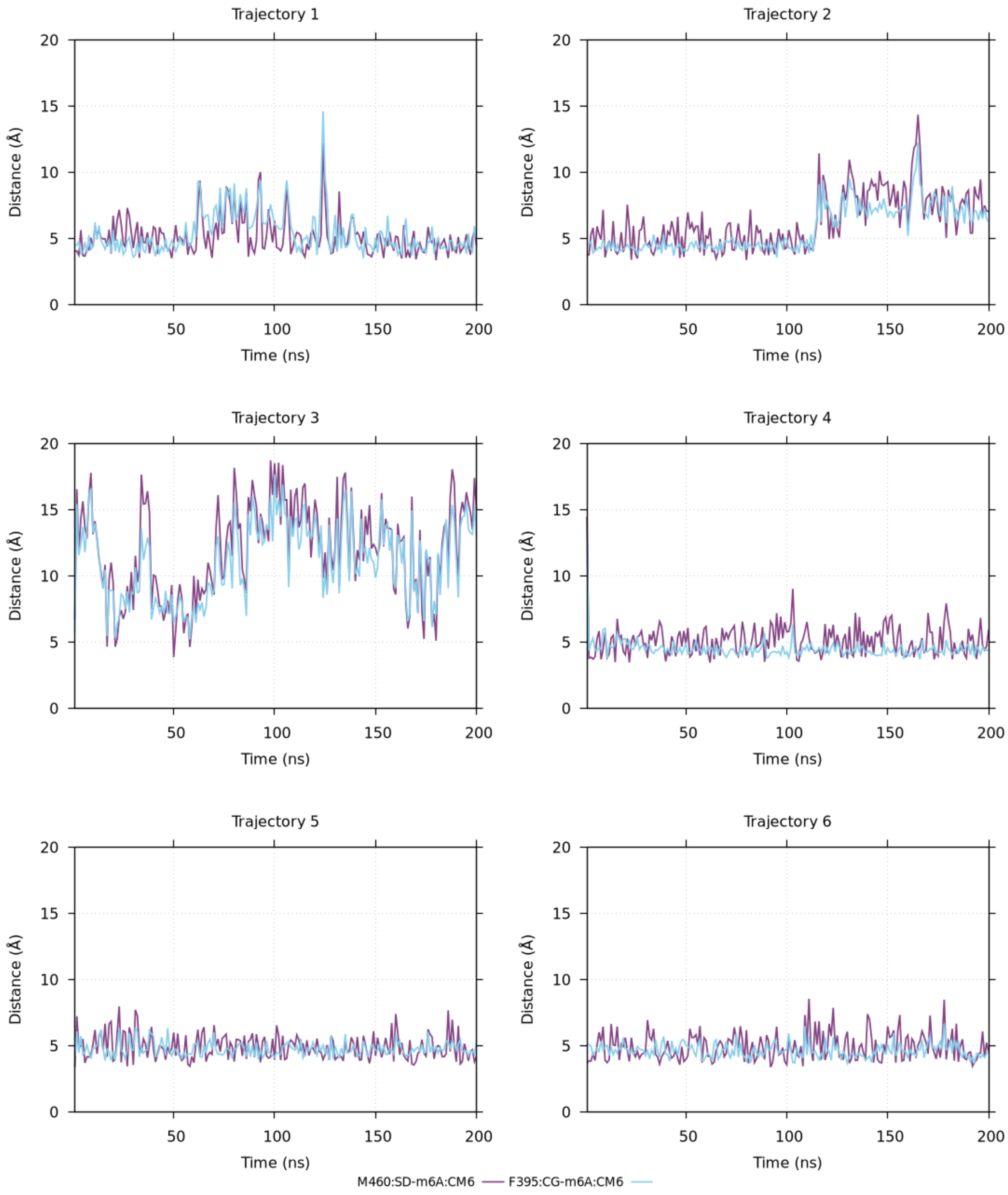

Figure S32. Time evolution of the distance (Å) between M460:SD-m^{6}A:CM6 and F395:CG-m^{6}A:CM6 during MD simulation of 5′-G-m^{6}A-CUGG-3′ in complex with RRM3 domain in the context of full-length RBM45 protein. The plots show the distance between the indicated atom pairs measured across three independent trajectories (200 ns each). In each panel, the purple and blue line correspond to the distances for the two-protein residues and m^{6}A pairs, M460-m^{6}A and F395-m^{6}A, respectively.

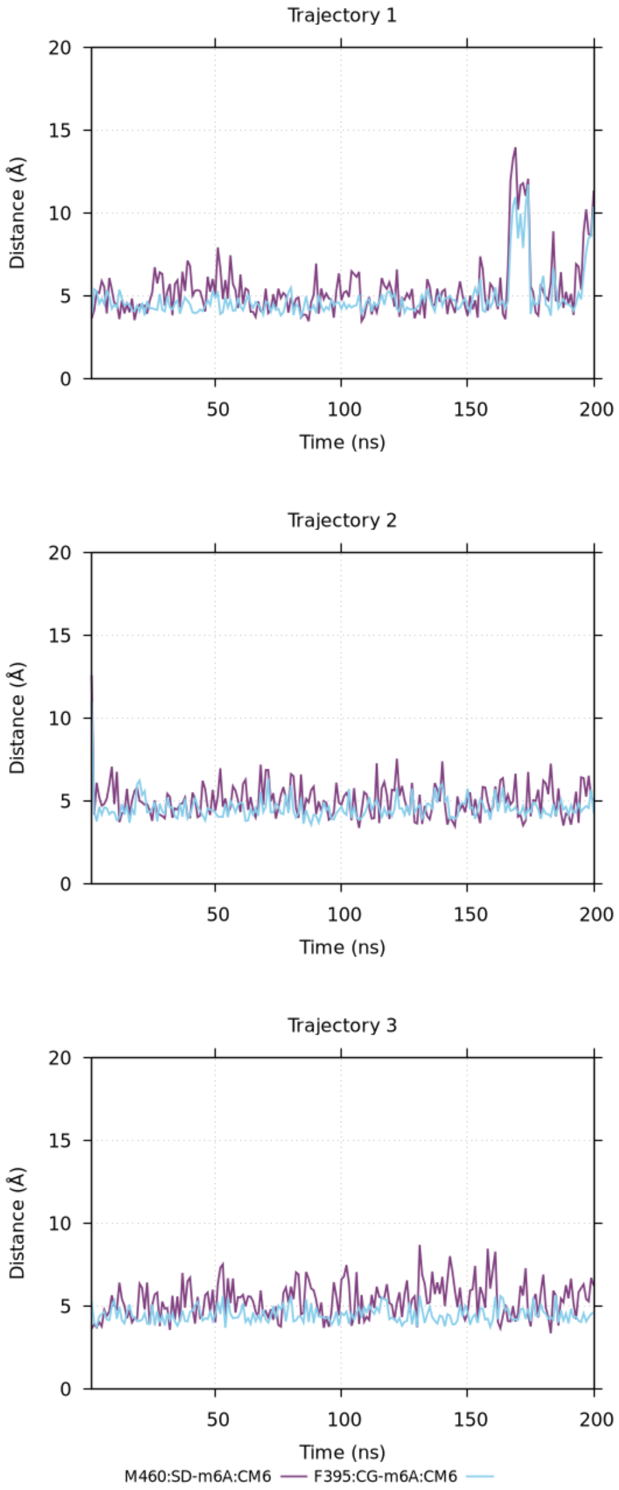

Supplement: Supplementary file 1 [file jp6c01856_si_001.pdf]
